# Synergistic ionic modification strategy enhances the stability of naphthalene diimide zwitterions for cost-effective aqueous organic redox flow batteries

**DOI:** 10.1093/nsr/nwaf123

**Published:** 2025-04-07

**Authors:** Heng Zhang, Chenjing Liu, Zengrong Wang, Xu Liu, Zhikang Han, Xuri Zhang, Yawen Li, Qing Zhao, Gang He

**Affiliations:** Frontier Institute of Science and Technology, Interdisciplinary Research Center of Frontier Science and Technology, State Key Laboratory for Strength and Vibration of Mechanical Structures, Xi'an Jiaotong University, Xi'an 710054, China; Frontier Institute of Science and Technology, Interdisciplinary Research Center of Frontier Science and Technology, State Key Laboratory for Strength and Vibration of Mechanical Structures, Xi'an Jiaotong University, Xi'an 710054, China; Frontier Institute of Science and Technology, Interdisciplinary Research Center of Frontier Science and Technology, State Key Laboratory for Strength and Vibration of Mechanical Structures, Xi'an Jiaotong University, Xi'an 710054, China; Frontier Institute of Science and Technology, Interdisciplinary Research Center of Frontier Science and Technology, State Key Laboratory for Strength and Vibration of Mechanical Structures, Xi'an Jiaotong University, Xi'an 710054, China; Frontier Institute of Science and Technology, Interdisciplinary Research Center of Frontier Science and Technology, State Key Laboratory for Strength and Vibration of Mechanical Structures, Xi'an Jiaotong University, Xi'an 710054, China; Frontier Institute of Science and Technology, Interdisciplinary Research Center of Frontier Science and Technology, State Key Laboratory for Strength and Vibration of Mechanical Structures, Xi'an Jiaotong University, Xi'an 710054, China; Frontier Institute of Science and Technology, Interdisciplinary Research Center of Frontier Science and Technology, State Key Laboratory for Strength and Vibration of Mechanical Structures, Xi'an Jiaotong University, Xi'an 710054, China; Key Laboratory of Advanced Energy Materials Chemistry (Ministry of Education), Nankai University, Tianjin 300071, China; Frontier Institute of Science and Technology, Interdisciplinary Research Center of Frontier Science and Technology, State Key Laboratory for Strength and Vibration of Mechanical Structures, Xi'an Jiaotong University, Xi'an 710054, China; Shaanxi Key Laboratory of New Conceptual Sensors and Molecular Materials, Engineering Research Center of Key Materials for Efficient Utilization of Clean Energy of Shaanxi Province, Xi'an Key Laboratory of Electronic Devices and Material Chemistry, Xi'an Jiaotong University, Xi'an 710054, China

**Keywords:** naphthalene diimide derivatives, synergistic effect of zwitterions, π-π interaction, stable configuration, decomposition resistance

## Abstract

Aqueous organic redox flow batteries (AORFBs) hold significant promise for energy storage due to their unique advantages and characteristics. However, their development is hindered by the lack of decomposition resistance and cycle stability over long periods. In this study, we synthesized naphthalene diimide (NDI) derivatives with zwitterions in their side chains via the atmospheric pressure method, namely **(CBu)_2_NDI** and **(SPr)_2_NDI**. The electrostatic repulsion between **(CBu)_2_NDI** precisely regulates π-π stacking into a parallel-staggered pattern. The synergistic zwitterions strategy effectively mitigates the positive charge of N^+^ in **(CBu)_2_NDI** compared with (NPr)_2_NDI and dex-NDI; this not only enhances the aromaticity of the naphthalene diimide core but also inhibits the side chain decomposition caused by the S_N_2 nucleophilic attack of hydroxyl ions (OH^−^) on the C=O. The calculation of the single point energy proves that during the charging processes of **(CBu)_2_NDI**, the K^+^ will be close to the naphthalene core to form dimers or monomers with lower energy configurations under electrostatic attraction. **(CBu)_2_NDI** achieved a water solubility up to 1.49 M, which can be paired with K_4_Fe(CN)_6_ under two-electron transfer with total electrolyte costs as low as $6.58 Ah^−1^. The 0.1 M battery maintains full capacity after 5070 cycles. Furthermore, the battery delivers an impressive 100% capacity retention under 2 M e^−^ during 220 cycles.

## Teaser

This study presents an innovative ionic synergistic strategy for synthesizing highly stable naphthalene diimide zwitterionic derivatives, enabling cost-effective AORFB that exhibits long-term cycling stability at a high electron concentration of 2 M e- without significant attenuation.

## INTRODUCTION

In recent years, the global energy issue has become increasingly prominent and the development of new energy has become a vital way to address the energy crisis [1]. Although new energy has significant advantages of sustainability and autonomy, its indirectness, randomness, and uneven geographical distribution cannot be ignored. Therefore, there is an urgent need to develop large-scale energy storage equipment with high safety, flexible expansion, long lifespan, and low cost to better address the challenges in the application of new energy and promote sustainable development [2]. Due to its high energy density, long lifespan, and excellent safety performance, the flow battery is considered a promising candidate among large-scale energy storage devices [3–6]. It can seamlessly integrate with renewable energy generation systems to achieve stable power output and efficient utilization of electricity [7–9]. Notably, neutral aqueous organic redox flow batteries (AORFBs) demonstrate even greater potential in the field of flow batteries, thanks to their modifiable molecular structure [5,10], superior reaction kinetics and reversibility [11], low cost, high safety, and environmental friendliness [12–14]. Numerous molecules with excellent redox properties, such as viologens [15–20], anthraquinones [21–23], phenazines [24–27], fluorenone [28], and imides [29–31], have been extensively studied and utilized as anolytes. Among them, the two-electron energy storage materials have significant advantages in improving the energy density and power density of batteries [32]. Therefore, how to improve the cycle stability and life of two-electron materials has become an important research direction of AORFBs.

Naphthalene diimide–based materials with a naphthalene ring as the cornerstone establish a stable π-π–conjugated system [33–37]. This system allows π-electrons to freely flow within the naphthalene ring, significantly enhancing the electronic transport capacity of the molecules [38–40]. Recently, with their ultra-stable two-electron transfer characteristics, they have demonstrated broad application prospects in the field of neutral AORFBs. More recently, naphthalene diimide materials were proposed for application in neutral AORFBs [41]. In 2020, Byon and coworkers reported on water-soluble naphthalene diimide derivatives (K_2_BNDI, Na_2_BNDI) utilizing carboxylic acid groups [30]. But these molecules modified only with anions show low solubility and poor cycle stability. In 2022, the He group successfully synthesized a series of molecules (NDI, PDI, TPDI), which further revealed the significance of the planar rigid conjugated structure of imide molecules in maintaining the stability of highly reduced species [29]. By 2024, this group successfully regulated the synergistic effect between π-π stacking and hydrogen bonding, resulting in a solubility of up to 1.85 M for dex-NDI. Furthermore, the battery demonstrated exceptional capacity retention over 100 cycles at a high electron concentration of 2.4 M [42]. However, despite the promising research achievements of naphthalene diimide materials in the field of flow batteries, there are still several challenges that need to be addressed. First, an increase in pH can lead to irreversible decomposition of the naphthalene diimide side chain or diimide rings during the battery cycling process, subsequently causing a decrease in the solubility of molecules and even affecting the redox performance [31]. Moreover, the π-π interaction force enhances the intermolecular aggregation ability in the highly reactive radical state, resulting in a sharp increase in viscosity, thereby damaging the performance and lifespan of the battery. Therefore, it is imperative to develop novel, high-performance naphthalene diimide anolytes to address decomposition, severe accumulation issues during cycling and cost concerns.

Based on these considerations, this work has successfully synthesized a series of zwitterionic naphthalene diimide derivatives with both high performance and cost-effectiveness by employing the atmospheric pressure method [42,43]. The design strategy increases the charged centers of the molecule, leading to electrostatic interactions that effectively modulate the π-π interactions between molecules. The π-π stacking pattern among the molecules was verified through single-crystal diffraction analysis and Materials Studio techniques. Utilizing precise single-point energy calculations, the structural evolution of the molecules has been analyzed under different states during the charging process [31]. By comparing the changes in Nuclear Independent Chemical Shift (1) [NICS(1)], nuclear magnetic resonance (NMR) titration, the compressed Fukui function and Hirshfeld charge analyses (f^−^ and f^+^), the pivotal role of cation-anion synergy in enhancing the cycle stability and inhibiting the decomposition of molecules were uncovered. Notably, the constructed battery system exhibited no significant capacity degradation during extended cycling at an electron concentration of 2 M. This redox flow battery boasts ultra-high capacity and long lifespan, providing crucial insights and ideas for the application of naphthalene diimide in flow batteries.

## RESULTS AND DISCUSSION

### Synthesis of (SPr)_2_NDI, (SPrOH)_2_NDI and (CBu)_2_NDI

**Figure 1.**
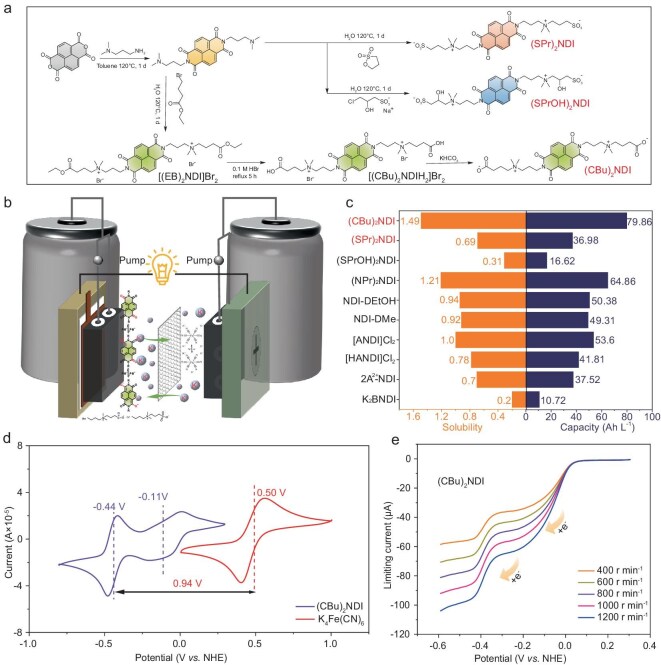
(a) Synthesis route of **(SPr)_2_NDI, (SPrOH)_2_NDI**, and **(CBu)_2_NDI**. (b) Schematic configuration of the AORFBs and reversible redox reaction pathways of **(SPr)_2_NDI** and **(CBu)_2_NDI** anolytes with **K_4_Fe(CN)_6_** catholyte during the charge-discharge process. (c) The solubility in water (orange) and capacity (blue) at room temperature. (d) CV of **(CBu)_2_NDI** with **K_4_Fe(CN)_6_**. Condition: 4 mM, 0.5 M KCl solution, 0.1 V s^−1^. (e) LSV of **(CBu)_2_NDI** at different rotation rates. Condition: 1 mM, 0.5 M KCl solution. 5 mV s^−1^.

In this paper, we have adopted the atmospheric pressure heating method to replace the previous hydrothermal method. This improvement avoids the uneven reaction phenomenon caused by the inability to stir in the hydrothermal method, thereby enhancing the quality and uniformity of material preparation. The atmospheric pressure heating method is not only simple to operate but also facilitates real-time monitoring, enabling better reaction control [43]. In the first step, 1,4,5,8-naphthalenetetracarboxylic dianhydride (NTCDA) with 3-(Dimethylamino)-1-propylamine were mixed in a sealed tube, a yellow crystal was obtained successfully through a condensation reaction, namely **NDI-N** [42]. In the second step, **(SPr)_2_NDI, (SPrOH)_2_NDI** and **[(EB)_2_NDI]Br_2_** were obtained through alkylation reactions. Subsequently, [**(CBu)_2_NDIH_2_]Br_2_** can be gained by hydrolysis of **[(EB)_2_NDI]Br_2_**. After deprotonation of **[(CBu)_2_NDIH_2_]Br_2_, (CBu)_2_NDI** is given (Fig. 1a). It is worth noting that during the deprotonation process, weakly alkaline salts were used such as KHCO_3_ or NaHCO_3_ to avoid decomposition and ring-opening of the diimide core in an alkaline environment (Fig. S1). Finally, we verified the structural correctness of the obtained products through nuclear magnetic resonance hydrogen spectroscopy, carbon spectroscopy, and high-resolution mass spectrometry.

### Solubility of (SPr)_2_NDI, (SPrOH)_2_NDI and (CBu)_2_NDI

Detailed measurements of the solubility of these four molecules were conducted using UV/Vis absorption spectroscopy (Fig. S2). For **(SPr)_2_NDI**, its solubility in water is 0.69 M. In 1 M NaCl, its solubility decreases significantly to only 0.33 M (Figs S3 and S4). The solubility of **(SPrOH)_2_NDI** in water is only 0.31 M (Fig. S3) [43], and the solubility of **[(CBu)_2_NDIH_2_]Br_2_** is relatively low, achieving only 0.13 M in water. When **[(CBu)_2_NDIH_2_]Br_2_** is deprotonated, the resulting **(CBu)_2_NDI** exhibits a significant increase in water solubility reaching 1.49 M (Fig. S3), relative to 79.86 Ah L^−1^. Compared to **[(CBu)_2_NDIH_2_]Br_2_**, the increased solubility can be attributed to two main reasons: first, the presence of more charges in the **(CBu)_2_NDI** molecule results in greater polarity, which favors its dissolution in water [44]. Second, the existence of Br^−^ in the **[(CBu)_2_NDIH_2_]Br_2_** molecule impacts its solubility [45]. In 1 M NaCl solution, its solubility slightly decreases to 1.32 M (Fig. S4). Comparison of solubility of some naphthalene diimide molecules is shown in Fig. 1c, including NDI-DEtOH [46], NDI-DMe [46], [HANDI]Cl_2_ [47], [ANDI]Cl_2_ [47], 2A^2+^-NDI [48], K_2_BNDI [30].

### Electrochemical characterization of (CBu)_2_NDI, (SPr)_2_NDI and (SPrOH)_2_NDI

Comprehensive electrochemical performance tests on the obtained products were measured using various methods. Figure S5 clearly showed the three molecules exhibited two sets of reversible redox peaks in the CV tests. The half-wave potential (*E*_1/2_) was taken as the average of the oxidation peak potential (*E*_pa_) and the reduction peak potential (*E*_pc_) [49]. Specifically, the redox peak potentials of **(SPr)_2_NDI** are *E*_1_ = −0.11 V and *E*_2_ = −0.43 V; for **(SPrOH)_2_NDI**, they are *E*_1_ = −0.11 V and *E*_2_ = −0.44 V; and for **(CBu)_2_NDI**, the potentials are *E*_1_ = −0.11 V and *E*_2_ = −0.44 V. Furthermore, consistent results were observed in the DPV tests of all substances (Fig. S6). When **(CBu)_2_NDI** is used as anolyte and **K_4_Fe(CN)_6_** is used as catholyte to form a neutral AORFB, the battery voltage window is up to 0.94 V (Fig. 1b and d). It is noteworthy that **(CBu)_2_NDI** under different pH was tested at a scan rate of 0.1 V s^−1^, and the positions and trends of its redox potentials are clearly demonstrated in Fig. S7a. As the pH gradually increased, the proton concentration decreased, and the second redox peak shifted to a more negative position. This trend was particularly evident in the range of pH = 1 to 3. This process may be related to coupling under high proton concentration [41,50]. However, as the pH value further increased to neutral and weakly alkaline regions, the redox potentials of **(CBu)_2_NDI** remained almost unchanged. This indicates that there are two independent one-electron processes, specifically within pH = 4 to 11 (Fig. S7a and b). Nevertheless, when the pH value continued to rise into the strongly alkaline range, a significant change occurred in that the two redox peaks of the molecule completely disappeared (Fig. S7c). This phenomenon is attributed to the attack of OH^−^ on the imide group when the concentration of OH^−^ is excessively high, leading to the ring-opening reaction of the diimide core and consequently loss of redox capability [51]. This process occurs within the pH range of 12 to 14. To gain a more profound understanding of the electrochemical properties of **(CBu)_2_NDI, (SPr)_2_NDI, (SPrOH)_2_NDI**, linear sweep voltammetry (LSV) testing was analyzed (Fig. 1e and Figs S8–S10). A summary is shown in Table S1. The diffusion coefficient *D* and electron transfer rate constant *k_0_* of **(CBu)_2_NDI** for the first process are 3.54 × 10^−6^ cm^2^ s^−1^ and 1.03 × 10^−2^ cm s^−1^_._ For the second process they are 1.35 × 10^−6^ cm^2^ s^−1^ and 3.58 × 10^−2^ cm s^−1^_._

### X-ray single crystal structure of (CBu)_2_NDI

**Figure 2.**
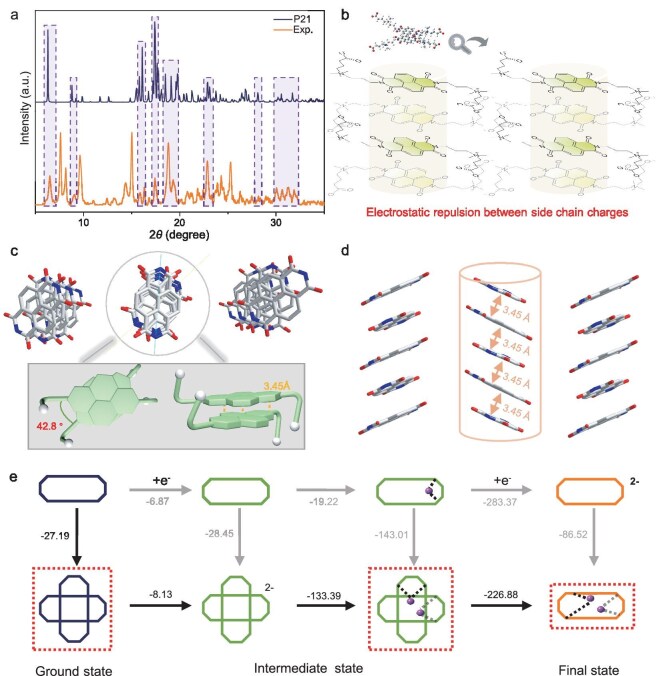
(a) The XRD patterns of **(CBu)_2_NDI**, experimental data (bottom) and simulation (top). The purple background represents the main peak. (b) Stacked structures of the **(CBu)_2_NDI** simulated by Material Studio. (c) Single-crystal XRD structure of **(CBu)_2_NDI**. (d) The π-π interactions are depicted as orange lines. Color code: N, blue; C, gray; O, red. Solvent molecules, side chain atoms and hydrogen atoms have been omitted for clarity. (e) The possible stable structures of **(CBu)_2_NDI** in the three states.

By gradually diffusing isopropyl ether into a methanol solution containing **(CBu)_2_NDI** at room temperature, a single crystal structure of **(CBu)_2_NDI** was obtained. In order to facilitate clear observation, Fig. 2c and d omitted the visual interference of side chains when presenting the molecular packing mode. Further analysis has revealed that **(CBu)_2_NDI** demonstrates a distinct parallel-staggered stacking pattern, characterized by π-π interactions with a face-to-face distance of 3.45 Å and a displacement stacking angle of 42.8°. Figure S11 depicts the single crystal packing in the presence of side chains.

### π-π interaction

Utilizing DFT (Density Functional Theory) computational methods, the stable configurations of **(CBu)_2_NDI** and **(SPr)_2_NDI** can be obtained after optimization. Furthermore, the molecular orbital energies of these two molecules in three different states were calculated (Figs S12, S13 and Table S2) [52]. From the perspective of electrostatic potential, it was evident that the naphthalene diimide core became increasingly negative during the obtaining of electrons (Fig. S14). The electron spin density indicated that the electron spin density of **(CBu)_2_NDI^1-^^·^** and **(SPr)_2_NDI^1-^^·^** is uniformly distributed across the naphthalene diimide core (Fig. S15). The unpaired electrons are delocalized through conjugation, thereby enhancing the stability of the radicals.

The unique planar structure and strong conjugated electronic system of naphthalene diimide materials provide excellent conditions for π-π stacking [53]. This structure enhances intermolecular attraction and benefits the stability of free radicals [31]. Through the analysis of XRD experimental data and Polymorph Predictor module, it well matches with the simulation curve of P_21_ compared to other crystal space groups, there is good agreement between the experimental and simulated diffraction peaks at 2*θ* = 6.54 (010) and 9.6 (002), and the positions of other main peaks are marked with a purple background (Fig. 2a), indicating a consistent crystal structure. This simulation curve clearly demonstrates that the molecules exhibit a parallel-staggered stacking pattern (Fig. 2b). The simulated pattern is the same as the stacking pattern shown earlier for single crystals. The reason for this stacking can be attributed to the mutual repulsion between side chains and charges. This enhanced interaction can effectively inhibit long-distance packing, mild π-π interactions induce the packing and disintegration of molecules, which in turn stabilizes the molecular conformation and maintains a high water solubility in different states [42,54,55]. In addition, the π-π interactions between naphthalene diimide molecules can to a certain extent inhibit the attack of dissolved oxygen on the molecules in the free radical state, thereby enhancing the stability of the molecules when charging and discharging [56].

After single-point energy calculations, the energy of the dimer **(CBu)_2_NDI** is lower than that of its monomer form (−27.2 kcal mol^−1^) in the ground state (Fig. S17). In the radical state, the single point energy of the dimer is also lower than that of the monomer (−28.5 kcal mol^−1^) (Fig. S18). Considering that after the molecule gets electrons, the naphthalene diimide core center will present a negative valence state, which may have electrostatic interaction with K^+^ in the electrolyte. The side and top views of the intermediate **(CBu)_2_NDI^1-^^·^** and final states **(CBu)_2_NDI^2^^−^** binding to K^+^ are shown in Fig. S19. Figure 2e vividly illustrates the most stable configurations that the three states can potentially attain during gaining electrons. It can be seen from the above calculation that **(CBu)_2_NDI** in their ground state tends to form aggregates in order to optimize their stability. Upon entering the intermediate radical state, **(CBu)_2_NDI** acquires a negative charge by capturing an electron. Consequently, K^+^ in the supporting electrolyte tends to move closer to it to restore electrical neutrality due to electrostatic attraction. Detailed energy analysis reveals the following: upon binding with K^+^, the energy of the system significantly decreases by 19.22 kcal mol^−1^, which is higher than dimerization (−28.5 kcal mol^−1^). Therefore, it is easier for the intermediate state to dimerize rather than to bind a K^+^, when this dimer binds with two K^+^, the most substantial energy reduction to 133.39 kcal mol^−1^ is obtained. These data conclusively support that the dimeric state of the intermediate molecule bound to K^+^ is energetically more favorable, making it the most preferred binding form. As for the final state, based on prior research [31], the naphthalene diimide core carrying two negative charges exhibits strong electrostatic interactions, prompting **(CBu)_2_NDI^2^^−^** to stabilize in its monomer form in the final state, accompanied by tight binding with two K^+^. Verified through energy calculations, the energy of the non-ionized monomer can be reduced by 86.52 kcal mol^−1^ upon binding with two K^+^. This result unequivocally indicates that in the final state, driven by the electrostatic balance mechanism, the molecule prefers to bind with two K^+^ to achieve the most stable configuration. The black arrows in the figure clearly delineate the most probable transformation pathway from the ground state to the final state. The numbers along this path indicate the energy changes at each step, visually illustrating the gradual enhancement of the system stability. Figure S20 shows the electrostatic potential of the optimal structure in the intermediate state and the electrostatic force between molecules. It can be seen that the presence of potassium ions can play a role in charge buffering, making the molecular structure more stable.

### Synergistic effect between zwitterions

**Figure 3.**
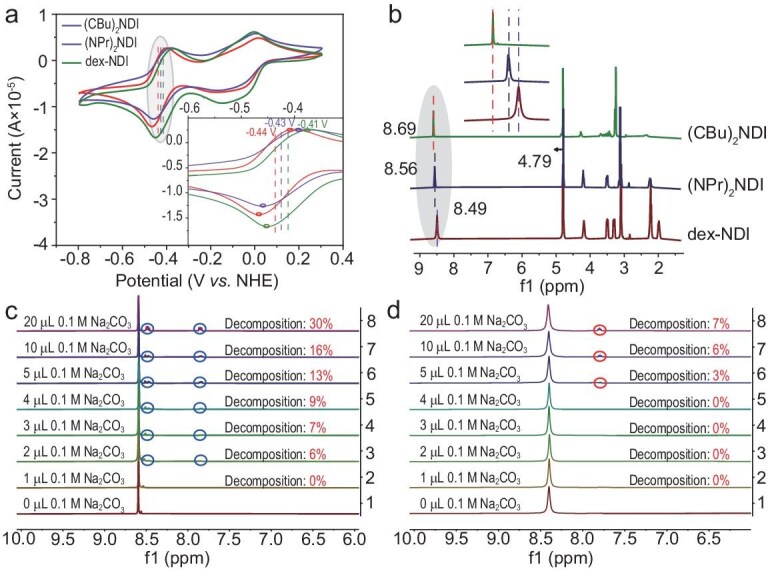
(a) Redox potentials of **(CBu)_2_NDI**, (NPr)_2_NDI, and dex-NDI. Inset: changes in the shift of the second redox peak for the three species. (b) The ^1^H NMR spectra of **(CBu)_2_NDI**, (NPr)_2_NDI, and dex-NDI. Inset: chemical shift of the protons in the diimide core for the three species. The initial ^1^H NMR spectra of 0.01 M (c) dex-NDI and (d) **(CBu)_2_NDI** at different 0.1 M Na_2_CO_3_ amounts.

By measuring the CV curve of three naphthalene diimide derivatives [**(CBu)_2_NDI**,(NPr)_2_NDI, dex-NDI], it is evident that **(CBu)_2_NDI** exhibits the most negative redox potential compared to (NPr)_2_NDI and dex-NDI, indicating **(CBu)_2_NDI** has a higher electron cloud density surrounding the naphthalene diimide core (Fig. 3a). In the ^1^H NMR spectrum, the introduction of the carboxyl group increased the electron density of the naphthalene core compared to (NPr)_2_NDI, affecting its protons and shifting them to a higher field (from 8.56 to 8.49 ppm). Conversely, compared to (NPr)_2_NDI, the introduction of quaternary ammonium salts reduced the electron density of the naphthalene diimide core, causing protons to shift towards a lower field (from 8.56 to 8.69 ppm) (Fig. 3b).

Furthermore, we employed nuclear magnetic resonance (NMR) titration to experimentally verify the decomposition resistance of **(CBu)_2_NDI** under weakly alkaline conditions. In the experiment, we tested the ^1^H NMR changes of 500 μL of 0.01 M dex-NDI and **(CBu)_2_NDI** in D_2_O upon the addition of varying volumes (1 μL, 2 μL, 3 μL, 4 μL, 5 μL, 10 μL, 20 μL) of 0.1 M Na_2_CO_3_ solution. The results indicated that dex-NDI began a ring opening reaction upon the addition of 2 μL Na_2_CO_3_ solution, and the decomposition rate reached 30% when 20 μL Na_2_CO_3_ was added (Fig. 3c). In contrast, the decomposition process of **(CBu)_2_NDI** was relatively slow, with decomposition initiating only upon the addition of 5 μL Na_2_CO_3_ solution, and even when 20 μL Na_2_CO_3_ was added, the ring opening rate was still only 7% (Fig. 3d). As the amount of Na_2_CO_3_ in the solution increases, its pH changes and these are shown in Table S3. When 20 μL of Na_2_CO_3_ were added, the pH value of dex-NDI reached 9.58, while the pH value of **(CBu)_2_NDI** had risen to 10.17, yet the amount of decomposition was less than that of dex-NDI. ^1^H NMR spectroscopy monitored the changes in the components over time after incrementally adding different volumes of Na_2_CO_3_ solution to dex-NDI and **(CBu)_2_NDI** solutions in D_2_O as in Figs S21–S24. The results indicated that when different volumes (1 μL, 2 μL, 3 μL, 4 μL) of 0.1 M Na_2_CO_3_ solution were added dropwise to dex-NDI and **(CBu)_2_NDI** solutions, the signal of decomposed dex-NDI gradually intensified over time. However, during the 10-day monitoring period, no significant changes were observed in the ^1^H NMR spectrum of **(CBu)_2_NDI**. This further proves the decomposition resistance of **(CBu)_2_NDI**.

**Figure 4.**
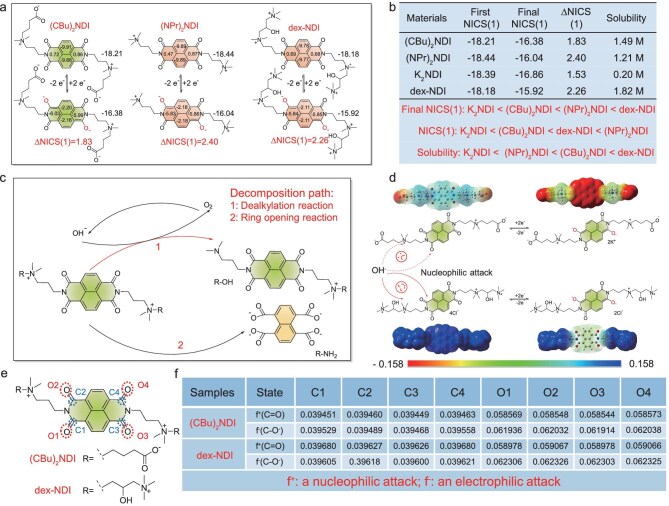
(a) The NICS(1) values of **(CBu)_2_NDI**, (NPr)_2_NDI and dex-NDI. (b) Diagram of NICS(1) and solubility of **(CBu)_2_NDI**, (NPr)_2_NDI, dex-NDI and K_2_NDI. (c) Schematic diagram of decalkylation and ring opening of naphthalene diimide derivatives under alkaline conditions. (d) Electrostatic potential, (e and f) Fukui function and Hirshfeld charge of **(CBu)_2_NDI** and dex-NDI.

The aromaticity of **(CBu)_2_NDI** in their three states was evaluated through Nuclear Independent Chemical Shift (1) [NICS(1)] calculations [57]. As shown in Fig. 4a, for the three molecules in their oxidized state, the naphthalene rings exhibit aromaticity, whereas the diimide rings display antiaromaticity. After gaining two electrons, the aromaticity of diimide rings is enhanced, while the aromaticity of the naphthalene rings has declined, yet they still retain their aromatic character. In this highly reduced state, the introduction of anionic groups (carboxylate) acts as a charge buffer for the N^+^ compared to (NPr)_2_NDI and dex-NDI. This effect promotes a greater concentration of π-electrons at the core, thereby enhancing the electron cloud density and enabling more π-electrons to participate in the conjugated structure of the entire molecular system [46]. So the NICS(1) value of the high reduction state of **(CBu)_2_NDI^2^^−^** (−16.38) is more negative than the quaternary ammonium modified (NPr)_2_NDI (−16.04) and dex-NDI^2+^(−15.92) [29], which theoretically indicates that **(CBu)_2_NDI^2^^−^** is more stable. Comparing the changes of NICS(1) [ΔNICS(1)] of the three NDI derivatives in both the ground state and the final state, it can be found that the ΔNICS(1) value of **(CBu)_2_NDI** (1.83) is smaller than (NPr)_2_NDI (2.40) and dex-NDI (2.26). A slightly higher ΔNICS(1) value compared to K_2_NDI is shown in Fig. S25, however, the solubility of **(CBu)_2_NDI** is seven times higher than that of K_2_NDI. Figure 4b summarizes the NICS(1) values and solubility of **(CBu)_2_NDI**, (NPr)_2_NDI, dex-NDI, and K_2_NDI, where it can be seen that **(CBu)_2_NDI** can simultaneously take into account the aromaticity and solubility of highly reduced species. During the charging process of naphthalene diimide derivatives, the solution gradually becomes weakly alkaline (Fig. S26). The enhancement of alkalinity promotes S_N_2 nucleophilic attack of OH^−^, leading to the dealkylation reaction (Figs S27 and S28) and ring opening. This process reduces solubility and causes irreversible capacity decay. Figure 4c shows the side reactions of naphthalene diimide derivatives. From Fig. 4d, it can be observed that the electrostatic potential of the naphthalene core and the side chain N⁺ atom in **(CBu)_2_NDI** is more negative compared to dex-NDI both in the ground state and the final state. The compressed Fukui function is an indicator used to measure the reactivity of different positions in a molecule towards nucleophilic and electrophilic attacks. By comparing the Fukui functions of the two molecules, we find that both the nucleophilic Fukui function (f^+^) for C=O form and electrophilic Fukui function (f^−^) for C−O^−^ of **(CBu)_2_NDI** are smaller than those of dex-NDI, which implies that **(CBu)_2_NDI** is less susceptible to electrophilic/nucleophilic attack compared to dex-NDI (Fig. 4e) [58], thereby increasing its stability against decomposition. This discovery profoundly reveals that the introduction of carboxylate for synergistic modification after the N^+^ plays a crucial role in maintaining the molecule's potential, solubility, aromatic stability, and decomposition resistance.

### Hydrogen bond interaction

There are oxygen atoms in **(CBu)_2_NDI** which can be used as hydrogen bond donors to form hydrogen bonds with water molecules (Fig. S29). Using MeOD as a deuterated reagent, the shift of the naphthalene diimide core peak (from 8.703 to 8.711 ppm) and the H_2_O peak (from 4.866 to 4.899 ppm) towards the low-field direction can be observed by monitoring the ^1^H NMR displacement changes under different water contents (10, 20, 30, 50, and 100 μL, respectively) (Fig. S30). This phenomenon indicates that **(CBu)_2_NDI** is preferred to form hydrogen bonds with H_2_O molecules in water [42], and this intermolecular interaction enables **(CBu)_2_NDI** to maintain a high water solubility in different states.

### Configuration evolution

A thorough investigation into the reversible conjugation mechanism of **(CBu)_2_NDI** was conducted during the charging and discharging process (Fig. S31) using UV/Vis spectrophotometry. In its initial state, the solution appeared colorless and exhibited two distinct absorption peaks at 361 nm and 380 nm (Fig. S33). As the battery voltage reached 0.59 V, **(CBu)_2_NDI** transformed into the **(CBu)_2_NDI^1-^^·^** radical after capturing its first electron (Fig. S32). At this point, the solution gradually changed to pale yellow, and the absorption peaks began to red shift and converged into a single peak at 448 nm. The **(CBu)_2_NDI^1-^^·^** captured its second electron when the voltage was further increased to 0.90 V, transforming into **(CBu)_2_NDI^2^^−^** and adopting a quinone structure. During this transition, the color of the solution shifted to purple, accompanied by the emergence of three distinct absorption peaks at 405 nm, 528 nm, and 570 nm. Consistent observations were made through chemical reduction (Figs S32 and S33). The radical states (**(CBu)_2_NDI^1-^^·^, (SPr)_2_NDI^1-^^·^**) during chemical reduction were detected by EPR (Fig. S34). After simulating the UV/Vis absorption spectrum of **(CBu)_2_NDI** in water, the simulation results were generally consistent with the experimental results (Fig. S35). Figures S36 and S37 captured the entire charging and discharging process of **(CBu)_2_NDI** and **(SPr)_2_NDI** in real-time through *in-situ* UV/Vis spectroscopy, which was completely reversible. During the 0% to 100% conversion process, a significant high-field shift phenomenon occurred at the H_a_ position, which is likely due to some electrostatic interaction between the side chain and the naphthalene diimide core (Fig. S38a) [42]. Meanwhile, electron paramagnetic resonance (EPR) was tested under different charging states (0%, 25%, 50%, 75%, and 100% SOC) of **(CBu)_2_NDI**. It was observed that the radical signal initially increased and then decreased, corresponding to the transition from monoradical species (**(CBu)_2_NDI^1-·^**) to dianion (**(CBu)_2_NDI^2^^−^**) and the dimers shield the radical signals, thereby affecting the changes in radical concentration (Fig. S38b) [47].

The ^1^H NMR and UV/Vis spectroscopic analyses of **(CBu)_2_NDI** in solution were monitored, the results presented in Fig. S39 indicated that there were no significant changes in the intensity and shift of the ^1^H NMR and UV/Vis absorption peaks within 15 days. These findings further confirm the chemical stability and resistance to decomposition of **(CBu)_2_NDI** in solution state. To further verify the structural stability of **(CBu)₂NDI** during the charge-discharge process, it was subjected to a 7-day ^1^H NMR monitoring, the data revealed that the proton signals of **(CBu)_2_NDI** remained unchanged (Fig. S40). This confirms the reversibility and structural integrity of **(CBu)_2_NDI**.

### Full battery test

**Figure 5.**
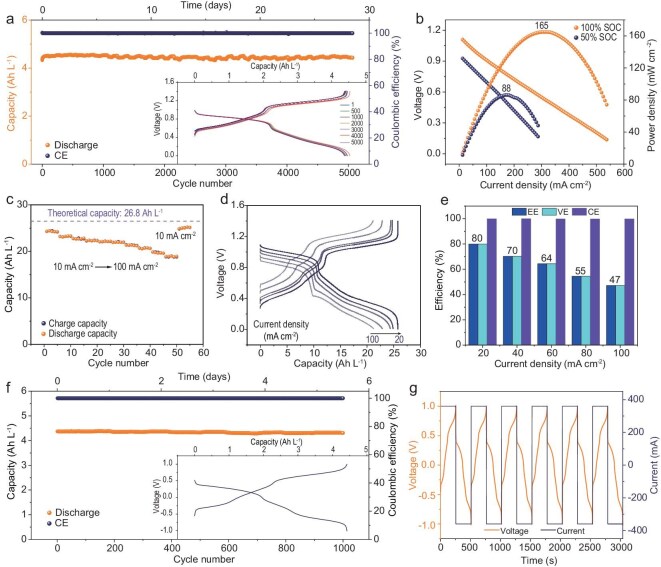
(a) The 0.1 M (0.2 M e^−^) **(CBu)_2_NDI/K_4_Fe(CN)_6_**-based AORFB was galvanostatically cycled at 40 mA cm^−2^ (inset: the charge and discharge curves of 1st, 500th, 1000th, 2000th, 3000th, 4000th, 5000th). (b) Polarization and power density curves of 0.5 M **(CBu)_2_NDI/K_4_Fe(CN)_6_**-based AORFB at 50% and 100% SOC, respectively. (c) Rate performance from 10 mA cm^−2^ to 100 mA cm^−2^. (d) The charge and discharge curves at different current densities. (e) Energy efficiency (EE), voltage efficiency (VE) and coulombic efficiency (CE) at different current densities. (f) Long-term cycling profile of the 0.1 M **(CBu)_2_NDI/(CBu)_2_NDI^2^^−^**-based AORFB (inset: the charge and discharge curves of representative cycle). (g) Example of symmetric battery tests.

The two synthesized materials were utilized as anolytes and combined them with **K_4_Fe(CN)_6_** to construct neutral aqueous organic redox flow batteries capable of two-electron storage. It is noteworthy that the lowest redox peak positions of these two materials stabilize around −0.44 V, while the redox peak position of **K_4_Fe(CN)_6_** is at 0.50 V. During the charging process, **(CBu)_2_NDI** and **(SPr)_2_NDI** as anolytes gradually capture two single electrons, transforming from keto-enamine isomers to quinone structures. Meanwhile, Fe^2+^ in **K_4_Fe(CN)_6_** releases one electron to transform into Fe^3+^ at the cathode side. Nafion 212 was employed as a cation exchange membrane to maintain charge balance, and its pre-treatment procedure is illustrated in Fig. S41. During full-battery testing, a 0.1 M (0.2 M e^−^) **(CBu)_2_NDI/K_4_Fe(CN)_6_**-based AORFB was employed (Fig. 5a). Testing was conducted under constant current charge-discharge conditions of 40 mA cm^−2^ within the voltage testing range of 0 V to 1.4 V. The results revealed an actual capacity of 4.51 Ah L^−1^ (with a theoretical capacity of 5.36 Ah L^−1^), resulting in a high-capacity utilization rate of 84.14%. Remarkably, the battery maintained a capacity retention of 100% after 5070 cycles, with a capacity fading rate of 0% per cycle. The minor fluctuations in the cycling curve after 1000 cycles are primarily caused by factors such as thermal effects, bottle collisions, changes in membrane resistance, or accidental human impacts, which disrupt the original equilibrium state of the battery [31,59]. The inset of Fig. 5a clearly illustrates the charge-discharge curves at various cycle numbers during the charging and discharging process. Figure 5b shows the rate and polarization of 0.5 M **(CBu)_2_NDI/K_4_Fe(CN)_6_**-based AORFB, under fully charged conditions using 5 mA cm^−2^, where the battery exhibited an output power of 165 mW cm^−2^. As the current density was gradually increased from 10 mA cm^−2^ to 100 mA cm^−2^, concentration polarization became increasingly evident. This phenomenon led to incomplete discharge of the materials, resulting in a gradual decrease in the discharge capacity of the battery. At different current densities, the discharge capacities were recorded from 24.43 Ah L^−1^ to 18.84 Ah L^−1^. Correspondingly, the capacity utilization remained high from 91.16% to 70.30%, indicating excellent capacity retention across various current densities (Fig. 5c). Figure 5d presents representative charge-discharge curves at current densities from 20 mA cm^−2^ to 100 mA cm^−2^ with an increment of 20 mA cm^−2^. Figure 5e illustrates the resistance of the battery increases at high current densities, leading to a decrease in both energy efficiency (EE) and voltage efficiency (VE).

A method that explored the stability of symmetric batteries was employed to investigate the stability of the molecules themselves. For the 0.1 M **(CBu)_2_NDI/(CBu)_2_NDI^2^^−^** system, after cycling 1000 times under a current density of 40 mA cm^−2^ and a charge-discharge cutoff voltage range of −1 V to 1 V, the capacity retention rate was 98.6%, with a single-cycle capacity decay rate of 0.0014% (Fig. 5f). For the 0.1 M **(SPr)_2_NDI/(SPr)_2_NDI^2^^−^** system, the same testing conditions as **(CBu)_2_NDI/(CBu)_2_NDI^2^^−^** were maintained but the current density was adjusted to 20 mA cm^−2^. After 1000 cycles, the capacity retention rate was 99.51%, with a capacity fading rate of 0.00049% per cycle (Fig. S42a). These results clearly demonstrate the exceptional cyclic stability exhibited by both substances. The voltage and current variations over time for the two molecules are depicted in Fig. 5g and Fig. S42b, respectively. Figure S43 further illustrates the charge-discharge curves at different cycle numbers during the charging and discharging process.

**Figure 6.**
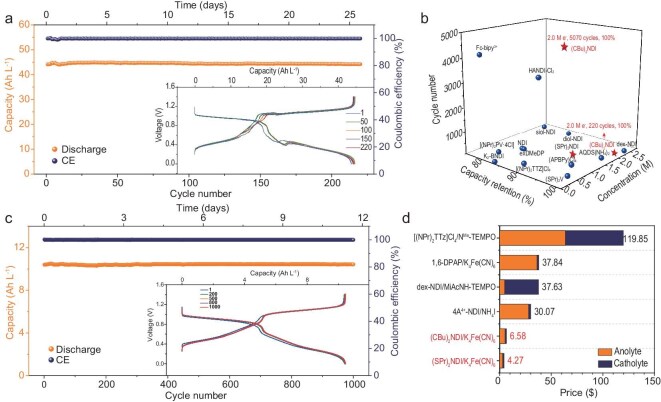
(a) The 1.0 M (2 M e^−^) **(CBu)_2_NDI/K_4_Fe(CN)_6_**-based AORFB was galvanostatically cycled at 20 mA cm^−2^. (b) Comparison of the electron concentrations, cycle numbers, and capacity retention of various anolytes of two-electron storage for neutral AORFBs (Table S5). (c) The 0.25 M (0.5 M e^−^) **(SPr)_2_NDI/K_4_Fe(CN)_6_**-based AORFB was galvanostatically cycled at 20 mA cm^−2^. (d) Total costs of electrolytes for typical two-electron storage for AORFBs for 1 Ah.

To further enhance the storage capacity of the battery, the electron concentration was increased to 2 M (Fig. 6a). Under galvanostatic cycling at 20 mA cm^−2^ between 0 and 1.4 V, the battery maintained a capacity retention of 100% after 220 cycles, with no capacity decay per cycle. The fluctuations in the charge-discharge curves may be attributed to the accumulation of the intermediate product **(CBu)_2_NDI^1-·^**, which increases the viscosity and reduces the reaction rate. Remarkably, the actual capacity reached a high value of 44.13 Ah L^−1^, resulting in a capacity utilization of 82.3%. Next, the cycle stability of **(SPr)_2_NDI** was tested (Fig. 6c), when the material concentration was 0.25 M, effectively doubling the electron concentration to 0.5 M. Under a current density of 20 mA cm^−2^ within 0–1.4 V, the battery exhibited remarkable stability after 1000 cycles with a capacity fading rate of 0% per day. The actual capacity achieved was 10.45 Ah L^−1^ (compared to the theoretical capacity of 13.4 Ah L^−1^). Figure S44a and b depicted the polarization and rate curves based on 0.1 M **(SPr)_2_NDI/K_4_Fe(CN)_6_**. Notably, the battery achieved a peak output power of 119 mW cm^−2^ at full charge. The trend in capacity variation with increasing rates from 10 to 100 mA cm^−2^ was similar to that observed in the **(CBu)_2_NDI/K_4_Fe(CN)_6_**-based battery(Fig. S44b). Figure S44c and d further present representative charge-discharge curves and EE, VE and CE at various current densities, respectively. Currently, the comparison of electron concentrations, cycle numbers, and capacity retention of various two-electron storage anolytes is shown in Fig. 6b (Table S5) [8,15–17,19,29,30,42–45,47,50,60–66]. Compared to other materials, the **(CBu)_2_NDI** has demonstrated remarkable performance, maintaining stable capacity even under ultra-long cycle numbers.

After long-term cycle testing, the battery was analyzed by conductivity, electrochemical impedance spectroscopy (EIS), CV scanning, ^1^H NMR and UV/Vis analysis. It can be observed that as the concentration increases, the conductivity of **(CBu)_2_NDI** gradually decreases (from 58.0 to 39.1 mS cm^−1^). Moreover, the conductivity of the solution after cycling is significantly lower than that of the fresh solution (Fig. S45), this may be related to the increased viscosity of the solution (Fig. S46). Figure S47 showed that the internal resistance of the cycled battery increased compared to a fresh battery. When comparing the CV curve after cycling with the original CV curve, no additional peaks were observed at the anolytes or catholyte, indicating that there was no significant electrolyte leakage during the cycling process (Fig. S48). Cyclic voltammetry was used to scan the recycled solution for 100 cycles after dilution several times, and the substance still maintained a good CV peak, which indicated that **(CBu)_2_NDI** still had a good redox ability (Fig. S49). The ^1^H NMR analysis revealed that a small number of impurity peaks emerged in the low-field region of the ^1^H NMR spectrum after a 20-day cycle. (Fig. S50a). This suggests a slight decomposition of **(CBu)_2_NDI**, but it maintains good structural stability. A 2-week UV/Vis test (Fig. S50b) was conducted on the post-cycling **(CBu)_2_NDI** solution, which showed no significant difference compared to the fresh solution. No additional peaks or peak shifts were observed, and the peak intensities remained unchanged over the long period, confirming the structural stability of **(CBu)_2_NDI** after cycling. To explore the underlying reasons, it is known that the parallel ion channel width of the Nafion 212 membrane is 2.4 nm [17]. Through structural modeling, the dimensions of the **(CBu)_2_NDI** and **(SPr)_2_NDI** molecules were determined to be 3.28 × 0.94 × 0.69 nm and 3.31 × 0.94 × 0.71 nm. When considering vertical passage through the channel, both lengths of the two molecules exceed the channel width, preventing the negative electrolyte from penetrating the Nafion 212 membrane and entering the catholyte. When attempting passage in a parallel direction, the negative charges on the side chains repel the negative charges on the Nafion membrane, further hindering molecular interpenetration (Fig. S51). The electrolyte permeability of Nafion 212 was tested using an H-tube for 16 days, with UV/Vis spectroscopy monitoring the tested solution. The test results indicated that the permeability of **(CBu)_2_NDI** should be lower than 2.6 × 10^−14^ cm^2^ s^−1^ (Fig. S52). Therefore, the capacity decay issues caused by electrolyte interspersion during cycling is significantly reduced.

### Electrolyte cost comparison

Finally, the total costs of the electrolytes were calculated (all drug prices are from Energy Chemical which can be found at https://www.energy-chemical.com/front/index.htm). For **(CBu)_2_NDI** and **(SPr)_2_NDI**, their laboratory costs are as low as $0.376 g^−1^ ($4.27 Ah^−1^) and $0.229 g^−1^ ($2.91 Ah^−1^). A summary of the total costs of anolytes and catholytes for neutral AORFBs has been conducted and the results shown in Table S4. These results show that the cost of batteries based on **(CBu)_2_NDI/K_4_Fe(CN)_6_** and **(SPr)_2_NDI/K_4_Fe(CN)_6_** is as low as $6.58 Ah^−1^ and $4.27 Ah^−1^ in the laboratory, relatively. Compared with other electrolytes used in AORFBs (Fig. 6d) [31,42,61,67], the cost of the total electrolytes is reduced to 1/5 to 1/20. To reduce the waste of electrolyte, we recycled the material by recrystallization, achieving a high recovery yield of 76%. Cyclic performance tests conducted on the recovered **(CBu)_2_NDI** demonstrated that after 1000 cycles of 0.1 M **(CBu)_2_NDI/K_4_Fe(CN)_6_**-based AORFB, the capacity retention could still reach as high as 96.7% (Fig. S53), ^1^H NMR verified the effectiveness of the recovery (Fig. S50a). As a critical component of the total system cost, the electrolyte accounts for more than half of it. Therefore, effectively reducing its cost holds immense significance in advancing the commercial applications of neutral aqueous organic redox flow batteries.

## CONCLUSION

In conclusion, the zwitterions **(CBu)_2_NDI** and **(SPr)_2_NDI** were synthesized through the atmospheric pressure method as anolytes. The intermolecular electrostatic repulsion of **(CBu)_2_NDI** adjusts the π-π stacking pattern into a parallel staggered pattern with a distance of 3.45 Å and an angle of 42.8°. Additionally, the incorporation of carboxylate acts as a buffer for the positive charge on the N^+^, enhancing the aromatic stability and decomposition resistance compared to dex-NDI. Furthermore, more charged centers enable **(CBu)_2_NDI** to maintain excellent water solubility up to 1.49 M. During the charging process, the presence of K^+^ due to electrostatic interaction reduces the energy of molecular monomers or dimers, thereby playing a stabilizing role in the solution. Experimental data showed that the **(CBu)_2_NDI/K_4_Fe(CN)_6_**-based AORFB retained 100% capacity after 5070 and 220 cycles at electron concentrations of 0.2 M and 2 M, respectively. The total electrolyte cost for the full batteries is as low as $6.58 Ah⁻^1^ when combined with **K_4_Fe(CN)_6_** as catholyte. This work innovatively incorporates zwitterions into naphthalene diimide, which not only comprehensively regulates the various properties of the material, but also opens up a new pathway for the application of neutral aqueous organic redox flow batteries in large-scale energy storage.

## METHODS

### Solubility tests

The solubility of four molecules was tested in deionized water by UV/Vis spectroscopy. First, a saturated solution of naphthalene diimide derivatives was prepared. Second, the standard solution was prepared, a certain mass of naphthalene diimide derivatives was accurately weighed, and a quantitative amount of deionized water was added to calculate the concentration of the standard solution. The standard absorption spectrum curve was obtained by dilution of the standard solution step by step under UV/Vis spectrophotometer testing. We then took a small amount of the saturated solution and diluted it with a known magnification. The concentration was measured by UV/Vis spectroscopy. Finally, the concentration was calculated according to a pre-calibrated absorbance-concentration curve of known concentrations of imide derivatives.

### The cyclic voltammogram (CV) studies

All cyclic and differential pulsed voltammetry tests were performed in 0.5 M KCl electrolyte solution. The redox potential was referred to NHE. A glassy carbon electrode (d = 3 mm) was used as the working electrode. A platinum sheet (1 cm^2^) was used for the counter electrode. A silver wire coated with a layer of AgCl was used as a reference electrode, suspended in 3 M KCl electrolyte solution (Ag/AgCl *vs*. NHE).

### The electrochemical kinetics studies

All linear sweep voltammetry (LSV) studies were conducted using a CHI660E and a Pine in a three-electrode configuration. A glassy carbon rotating electrode (5 mm diameter) was used as the working electrode along with a platinum sheet counter electrode and an Ag/AgCl reference electrode (the same as used in LSV studies). Before data collection, the electrolyte was purged using Ar for 20 min to remove the oxygen dissolved in the electrolyte. LSV scans were recorded at a scan rate of 5 mV s^−1^.

### *In situ* UV/Vis spectra

The UV/Vis, computer, Neware instrument, and flow battery were connected in a glove box. Anolyte was stored in a customized cuvette (1 cm × 1 cm × 10 cm) and catholyte was stored in a sample bottle. The concentration of naphthalene diimides and K_4_Fe(CN)_6_ used are 10^−5^ M and 2 × 10^−5^ M for *in situ* UV/Vis spectroscopyand their solution volumes are 8 mL and 20 mL, respectively. The flow rate is 30 mL min^−1^. The current density is 0.5 mA cm^−2^. The data is recorded quickly during charging and discharging.

### Full battery tests

The battery test fixture comprises of two steel plates, two polytetrafluoroethylene insulation plates, two Cu plate collectors, two graphite plates, and two graphite-felts, which are separated by the cation-exchange membrane. All the batteries tested for stability have an active area of 9 cm^2^, while the rate and polarization tests are conducted using 4 cm^2^. The battery fixtures were purchased from Wuhan Zhisheng New Energy Co., Ltd. The electrolyte materials with the same concentration are dissolved in 1 M KCl. The anolyte material contains **(CBu)_2_NDI**, (SPr)_2_NDI, while the catholyte material is composed of K_4_Fe(CN)_6_. All batteries are two-electron storage. The electrolytes are pumped into the cell at a flow rate of 60 mL min^−1^ through a peristaltic pump (BT100 M, Baoding Chuangrui Precision Pump Co., Ltd., Hebei, China). The reservoirs are purged with Ar to displace any O_2_ in the system and then sealed.

### Symmetric battery tests

A total of 0.1 M naphthalene diimide materials (5.0 mL) were fully charged to a highly reduced state (100% SOC) against the excess 0.1 M K_4_Fe(CN)_6_ (12 mL), which was used as a catholyte. And then the K_4_Fe(CN)_6_ side was washed with deionized water and refilled with fresh 0.1 M naphthalene diimide materials (7 mL). The symmetric battery was cycled with galvanostatic charge–discharge (GCD) testing between −1.0 and +1.0 V.

## Supplementary Material

nwaf123_Supplemental_Files
