# Supplementary material for: Synergistic ionic modification strategy enhances the stability of naphthalene diimide zwitterions for cost-effective aqueous organic redox flow batteries
Source: Natl Sci Rev. 2025 Apr 7;12(5):nwaf123. doi: 10.1093/nsr/nwaf123 (PMC12042746; doi:10.1093/nsr/nwaf123)
Supplement: nwaf123_Supplemental_Files [file nwaf123_supplemental_files.zip › Supplementary data.pdf]

Synergistic ionic modification strategy enhances the stability of naphthalene diimide zwitterions for cost-effective aqueous organic redox flow batteries

Heng Zhang, Chenjing Liu, Zengrong Wang, Xu Liu, Zhikang Han, Xuri Zhang, Yawen Li, Qing Zhao, Gang He*

Abstract: Aqueous organic redox flow batteries (AORFBs) hold significant promise for energy storage due to their unique advantages and characteristics. However, their development is hindered the lack of decomposition resistance and cycle stability over long periods. In this study, we synthesized naphthalene diimide (NDI) derivatives with zwitterions lay in their side chains via the atmospheric pressure method, namely $(CBu)_2NDI$ and $(SPr)_2NDI$. The electrostatic repulsion between $(CBu)_2NDI$ precisely regulates π-π stacking into a parallel-staggered pattern. The synergistic zwitterions strategy effectively mitigates the positive charge of N^+ in $(CBu)_2NDI$ compared with $(NPr)_2NDI$ and dex-NDI, this not only enhances the aromaticity of the naphthalene diimide core but also inhibits the side chain decomposition caused by the S_N2 nucleophilic attack of hydroxyl ions (OH^-) on the C=O. The calculation of the single point energy proves that during the charging processes of $(CBu)_2NDI$, the K^+ will be close to the naphthalene core to form dimers or monomers with lower energy configurations under electrostatic attraction. $(CBu)_2NDI$ achieved a water solubility up to 1.49 M, which can be paired with $K_4Fe(CN)_6$ under two-electron transfer with total electrolyte costs as low as \$6.58 Ah^{-1}. The 0.1 M battery maintains full capacity after 5070 cycles. Furthermore, the battery delivers an impressive 100 % capacity retention under 2 M e- during 220 cycles.

Table of Contents

Table of Contents S2
Experimental procedures S3
1. Synthetic procedures S3
2. Failure mechanism of naphthalene diimide derivatives S6
3. UV/Vis spectra S6
4. Solubility tests S7
5. The cyclic voltammogram (CV) studies S9
6. The differential pulse curve (DPV) studies S10
7. The CV curve under different conditions S11
8. The electrochemical kinetics studies S12
9. X-ray single crystal structural analyses S15
10. Density functional theory (DFT) calculation. S17
11. The spin density calculation. S19
12. Materials Studio. S20
13. Single point energy S23
14. Electrostatic potentials and interactions in the presence of K^+ S25
15. Decomposition resistance test of $(CBu)_2NDI$ S25
16. Change in pH during charging S27
17. HRMS of decomposition products S28
18. Hydrogen bond interaction S29
19. Configuration changes S30
20. EPR test S31
21. Computed UV/Vis spectra S32
22. In situ UV/Vis spectra S33
23. ^1H NMR and UV/Vis characterization of $(CBu)_2NDI$ before cycling S35
24. ^1H NMR characterization of $(CBu)_2NDI$ in cycling S35
25. Treatment of ion-exchange membranes S36
26. Symmetric battery S37
27. Full battery tests S39
28. Conductivity, viscosity and ^1H NMR S40
29. Electrochemical Impedance Spectroscopy (EIS) S41
30. The cyclic voltammogram after the cycle of the battery test S42
31. ^1H NMR and UV/Vis characterization of $(CBu)_2NDI$ after cycling S43
32. Simulation of molecule penetration through Nafion membrane. S43
33. Permeability measurement of $(CBu)_2NDI$. S44
34. Electrolyte recovery experiment for $(CBu)_2NDI$. S45
35. ^1H, ^{13}C NMR spectra S46
36. Anolytes and catholytes price comparison S53
37. Comparison of two-electron storage materials for neutral AORFBs. S61
Reference S66

Experimental procedures

1. Synthetic procedures

Synthesis of NDI-N.

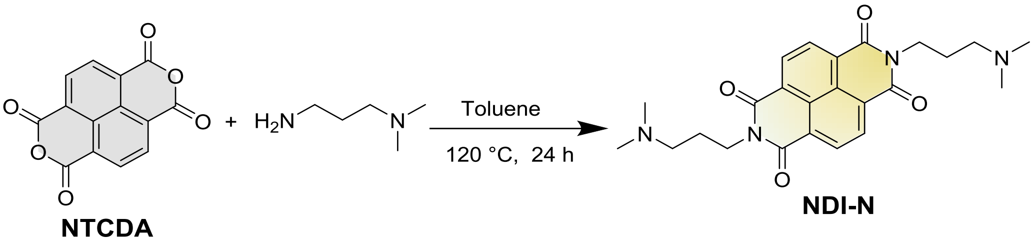

The NDI-N was synthesized and optimized following the literature procedure[1]. In a sealed tube, 1,4,5,8-Naphthalenetetracarboxylic dianhydride (NTCDA) (15 g, 56.0 mmol) was combined with 3-Dimethylaminopropylamine (17.15 g, 168 mmol) in 100 mL toluene. The solution was heated to 120 °C for 24 h under stirring. After the reaction, the product was filtered, washed three times with H_2O, ethanol, and dried overnight in a vacuum drying oven to obtain yellow crystal NDI-N. Yield: 23.19 g (95%). ^{1}H NMR (400 MHz, $CDCl_3$) δ 8.74 (s, 4H), 4.29 – 4.22 (m, 4H), 2.44 (t, J = 7.1 Hz, 4H), 2.23 (s, 12H), 1.96 – 1.87 (m, 4H). ^{13}C NMR (101 MHz, $CDCl_3$) δ 162.84, 130.90, 126.67, 126.62, 57.19, 45.38, 39.35, 25.97.

Synthesis of $(NPr)_2$NDI.

This compound was synthesized according to the reported procedures[1]. ^{1}H NMR (400 MHz, D_2O): δ 8.60 (s, J = 2.4 Hz, 4H), 4.23 (t, J = 6.9 Hz, 4H), 3.55-3.50 (m, 4H), 3.14 (s, 18H), 2.30-2.25 (m, 4H). ^{13}C NMR (100 MHz, D_2O): δ 163.47, 131.00, 125.57, 125.46, 63.95, 52.96, 37.70, 21.38.

Synthesis of $(SPr)_2$NDI.

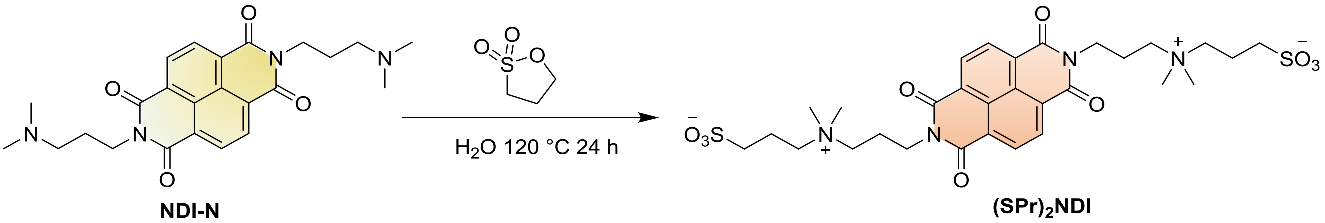

NDI-N (22.0 g, 50.3 mmol) was combined with 1,3-Propanesultone (14.3 g, 150.9 mmol) in 50 mL of H_2O in a sealed tube. The solution was heated to 120 °C for 24 h under stirring. After the reaction, ethanol and acetone were added to the resultant aqueous solution in sequential order in a 1:5 (product:ethanol) volume ratio to precipitate out the pure product. The product was filtered, washed with acetone for 3 times, and dried under vacuum to obtain white powder $(SPr)_2$NDI. Yield: 32.6 g (90%). ^{1}H NMR (400 MHz, Deuterium Oxide) δ 8.53 (s, 4H), 4.22 (s, 4H), 3.55 (s, 8H), 3.16 (s, 12H), 2.98 (s, 4H), 2.26 (t, J = 8.0 Hz, 8H). ^{13}C NMR (101 MHz, D_2O) δ 163.40, 131.00, 125.51, 62.25, 50.71, 47.15, 42.77, 18.16

Synthesis of $(CBu)_2NDI$.

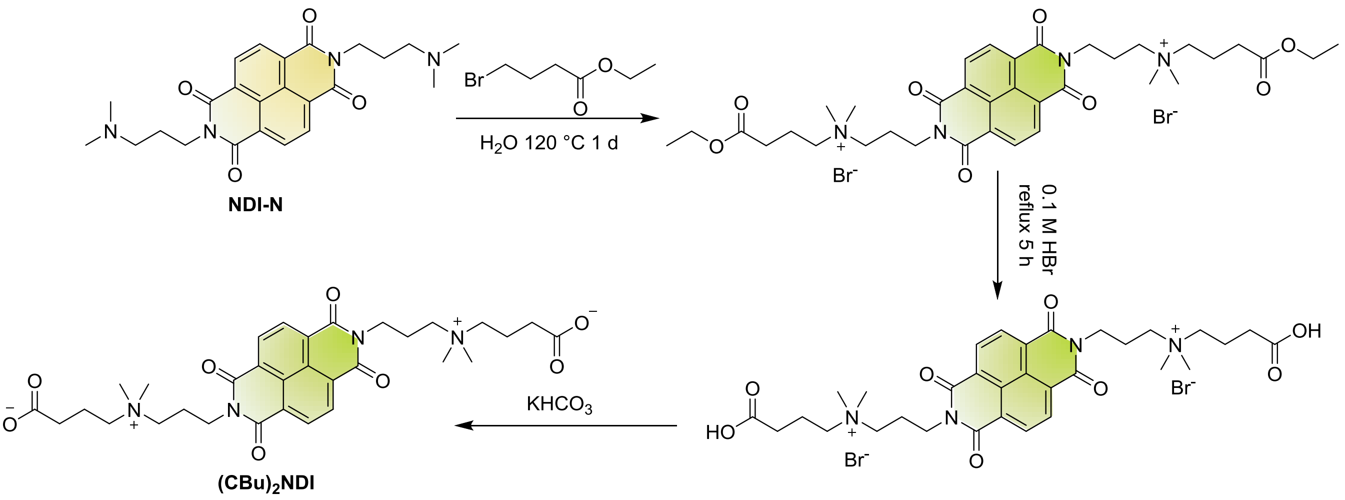

NDI-N (22.0 g, 50.3 mmol) was combined with ethyl 4-bromobutyrate (29.5 g, 150.9 mmol) in 20 mL of H_2O in a sealed tube. The solution was heated to 120 °C for 24 h. After the reaction, ethanol and acetone were added to the resultant aqueous solution in sequential order in a 1:8:2 (product:ethanol: acetone) volume ratio to precipitate out the pure product. The product was filtered, washed with acetone for 3 times, and dried under vacuum to obtain pale yellow powder $[(EB)_2NDI]Br_2$. Yield: 37.5 g (90%). ^1H NMR (400 MHz, Deuterium Oxide) δ 8.66 (s, 4H), 4.24 (s, 4H), 4.12 – 4.03 (m, 4H), 3.57 – 3.47 (m, 4H), 3.33 (d, J = 8.3 Hz, 4H), 3.10 (s, 12H), 2.45 (d, J = 7.4 Hz, 4H), 2.25 (s, 4H), 2.05 (s, 4H), 1.18 (td, J = 7.2, 2.4 Hz, 6H).

$[(EB)_2NDI]Br_2$ (10.9 g, 0.020 mol) was dissolved in 200 ml of 0.1 M HBr aqueous solution (85 times diluted with saturated hydrobromic acid) in a 500 ml round-bottom flask. This solution was heated to 100 °C and then refluxed for 5 hours. Water and excess HBr were removed through the rotary evaporator after reaction. Acetonitrile was added to round-bottom flask for washing and then filtration. Drying in an oven at 70°C overnight gave 9.35 g of a light yellow solid product $[(CBu)_2NDIH_2]Br_2$. Yield: 92%. $[(CBu)_2NDIH_2]Br_2$ (10.0 g, 13 mmol) and $KHCO_3$ (2.6 g,26 mmol) were added to a mixture of 40 mL H_2O: C_2H_5OH =1:8 for alkalinization. The mixture was heated to 60 °C and stirred for 12 h. After cooling to room temperature, a large amount of precipitate was precipitated, and the precipitate was washed with ethanol after filtration. $(CBu)_2NDI$ was dried overnight in an oven at 70 °C to give an off-white product. The yield was 82%(6.48 g). ^1H NMR (400 MHz, Deuterium Oxide) δ 8.57 (s, 4H), 4.21 (t, J = 6.4 Hz, 4H), 3.56 – 3.45 (m, 4H), 3.41 – 3.29 (m, 4H), 3.10 (s, 12H), 2.46 (t, J = 6.7 Hz, 4H), 2.25 (s, 4H), 2.04 (s, 4H). ^{13}C NMR (101 MHz, Deuterium Oxide) δ 180.52, 163.56, 131.01, 125.64, 63.76, 61.53, 50.59, 37.71, 33.40, 20.94, 19.04. HRMS (ESI) m/z: $[M+H]^+$ calcd for $C_{32}H_{40}N_4O_8$ 609.29189; found 609.29217.

Synthesis of dex-NDI.

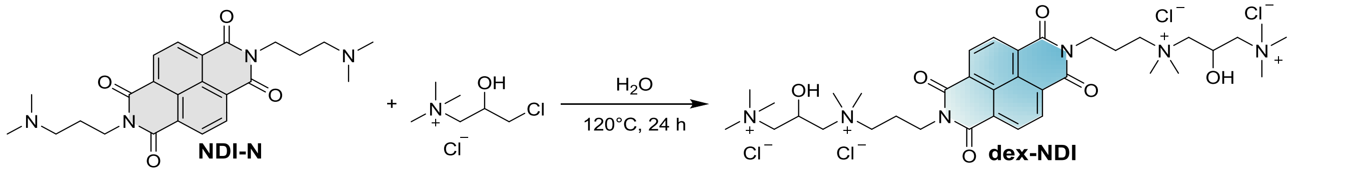

NDI-N (22.0 g, 50.3 mmol) was combined with 3-Chloro-2-hydroxypropyltrimethylammoniumchloride (28.5 g, 150.9 mmol) in 50 mL of H_2O in a sealed tube. The solution was heated to 120 °C for 24 h under stirring. After the reaction, ethanol and acetone were added to the resultant aqueous solution in sequential order in a 1:5 (product:ethanol) volume ratio to precipitate out the pure product. The product was filtered, washed with acetone for 3 times, and dried under vacuum to obtain white powder dex-NDI. Yield: 38.7 g (95%).^1H NMR (400 MHz, D_2O) δ 8.57 (s, 4H), 4.84 (t, J = 7.3 Hz, 2H), 4.29 – 4.17 (m, 4H), 3.68 – 3.36 (m, 12H), 3.22 (d, J = 8.2 Hz, 12H), 3.20 (s, 18H), 2.37 – 2.18 (m, 4H). ^{13}C NMR (101 MHz, D2O) δ 163.93, 131.03, 125.94, 125.86, 67.50, 65.20, 63.37, 61.76, 54.34, 52.07, 37.55, 21.08.

2. Failure mechanism of naphthalene diimide derivatives

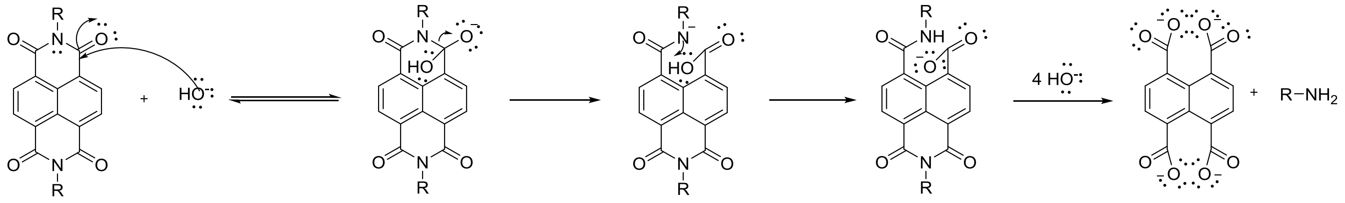

Figure S1. Decomposition mechanism of naphthalene diimide derivatives.

3. UV/Vis spectra

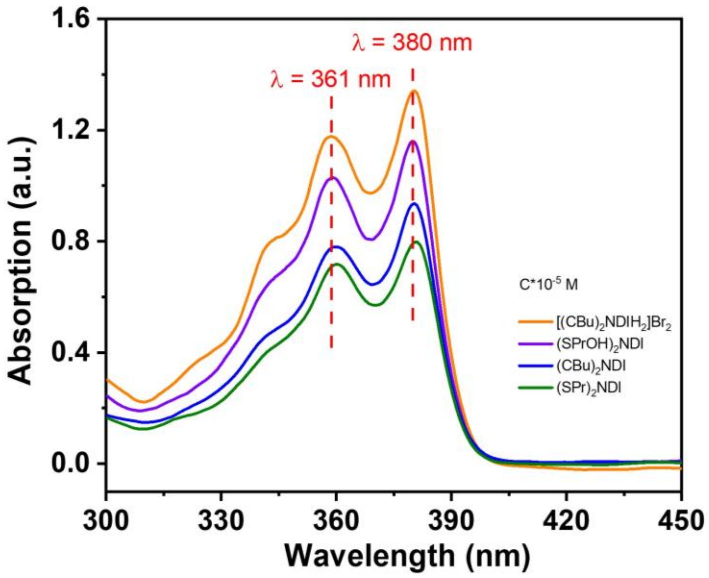

Figure S2. UV/Vis spectra of **$(SPr)_2$NDI**, **$(SPrOH)_2$NDI**, **[$(CBu)_2$NDIH_2]Br_2** and **$(CBu)_2$NDI** in Deionized water, c~10^{-5} M.

4. Solubility tests

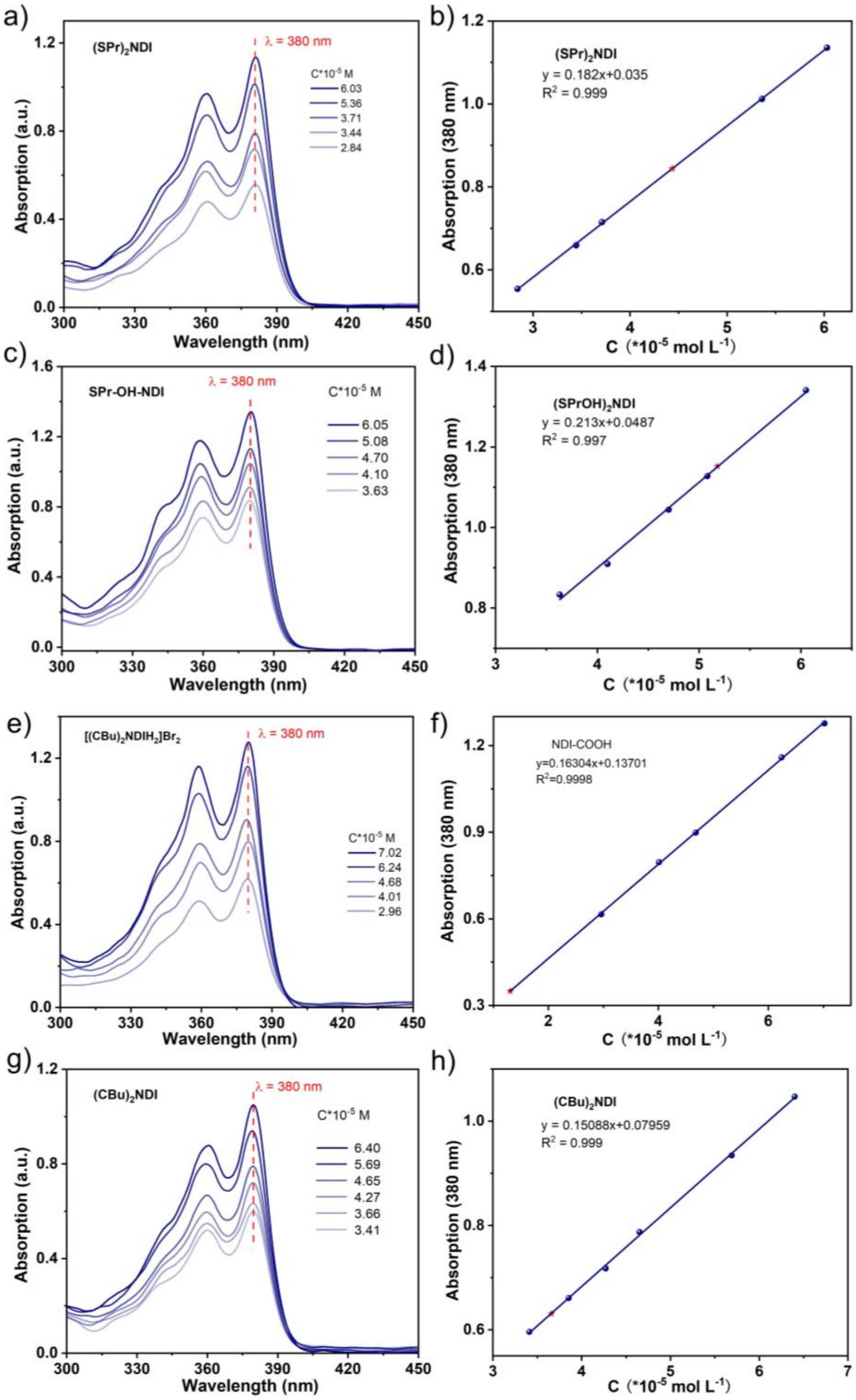

Figure S3. UV/Vis calibration lines for determination of the solubility of **$(SPr)_2NDI$**, **$(SPrOH)_2NDI$**, **$[(CBu)_2NDIH_2]Br_2$** and. **$(CBu)_2NDI$** UV/Vis spectrum for a) **$(SPr)_2NDI$**, c) **$(SPrOH)_2NDI$,** e) **$[(CBu)_2NDIH_2]Br_2$**, g) **$(CBu)_2NDI$** at different concentrations in Deionized water. The absorbance *versus* the concentration for b) **$(SPr)_2NDI$**, d) **$(SPrOH)_2NDI$**, f) **$[(CBu)_2NDIH_2]Br_2$**, h) **$(CBu)_2NDI$** at 380 nm.

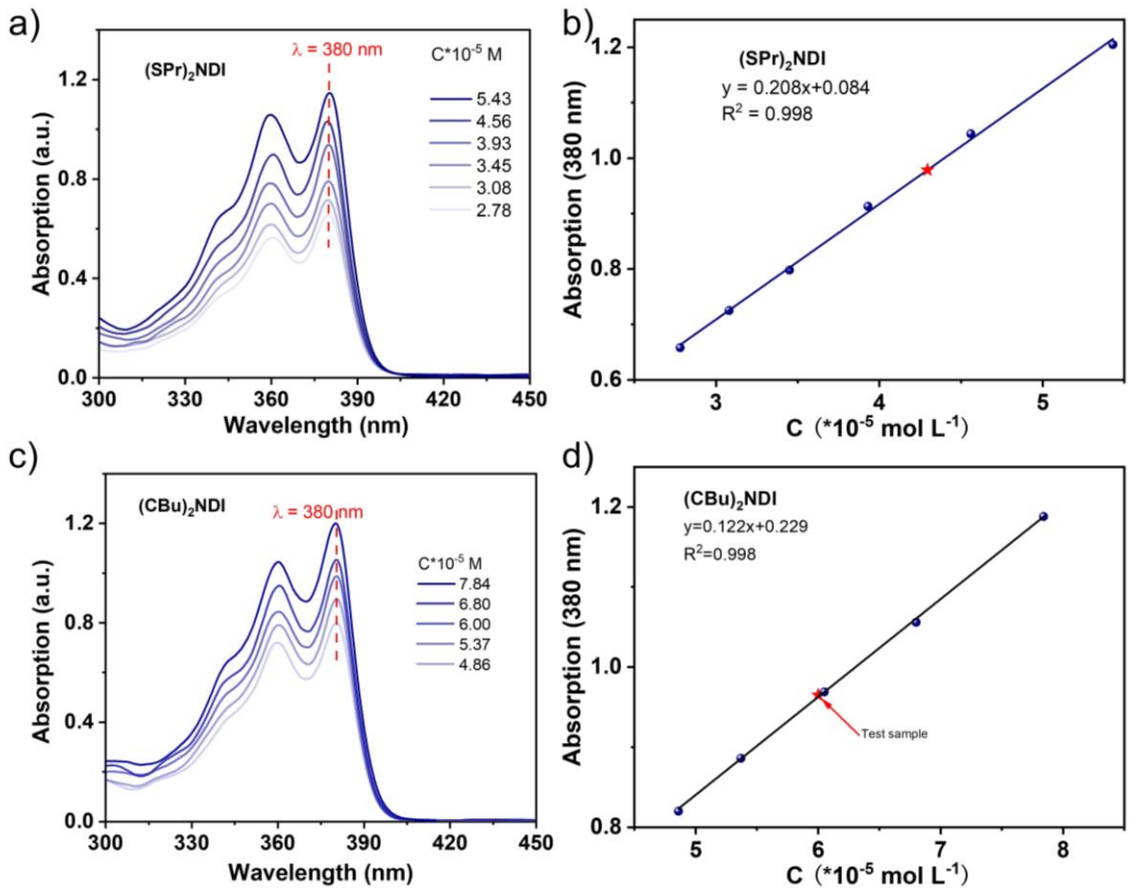

Figure S4. UV/Vis calibration lines for determination of the solubility of **$(SPr)_2NDI$** and **$(CBu)_2NDI$**. UV/Vis spectrum for a) **$(SPr)_2NDI$**, c) **$(CBu)_2NDI$** at different concentrations in 1 M KCl.The absorbance *versus* the concentration for b) **$(SPr)_2NDI$, d) $(CBu)_2NDI$** at 380 nm.

5. The cyclic voltammogram (CV) studies

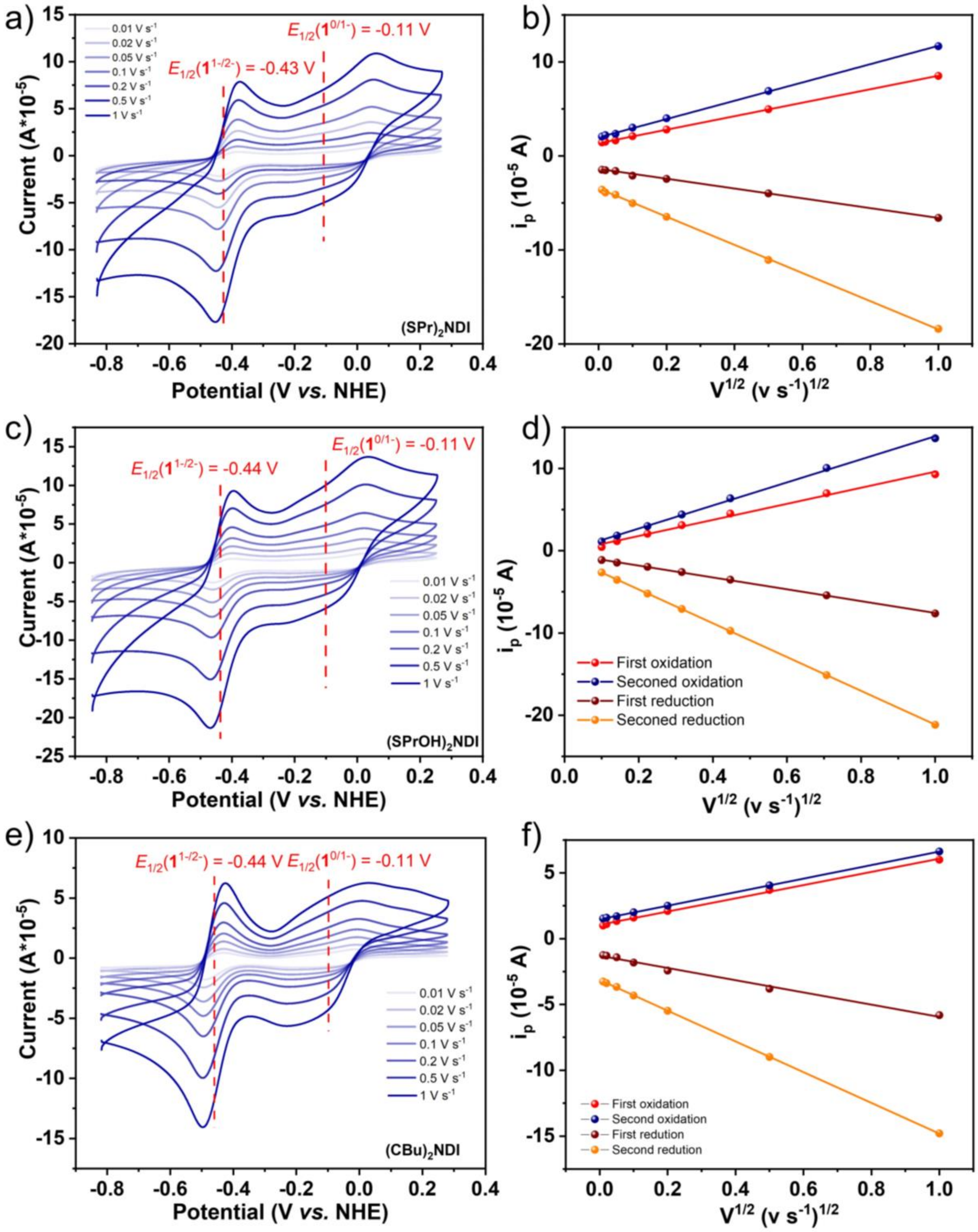

Figure S5. The cyclic voltammogram at different scan rates from 0.01 V s^{-1} to 1 V s^{-1} of three molecules, conditions: 4.0 mM a) **(SPr)$_2$NDI**, c) **(SPrOH)$_2$NDI,** e) **(CBu)$_2$NDI** in 0.5 M KCl electrolyte. The plot of peak current (i_p) over the square root of scan rates ($v^{1/2}$) for b) **(SPr)$_2$NDI**, d) **(SPrOH)$_2$NDI,** f) **(CBu)$_2$NDI**.

6. The differential pulse curve (DPV) studies

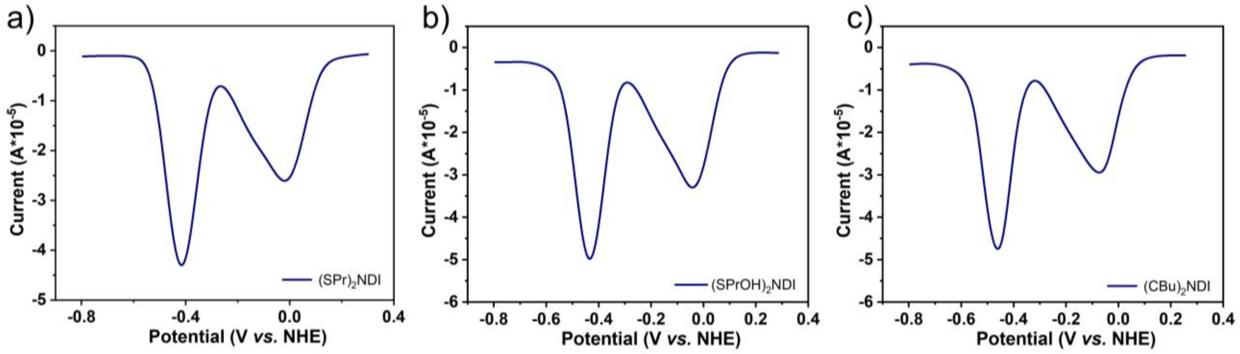

Figure S6. The differential pulse voltammetry with potential increment of 0.004 V; conditions: 4.0 mM a) **(SPr)$_2$NDI**, b) **(SPrOH)$_2$NDI**, c) **(CBu)$_2$NDI** in 0.5 M KCl electrolyte.

7. The CV curve under different conditions

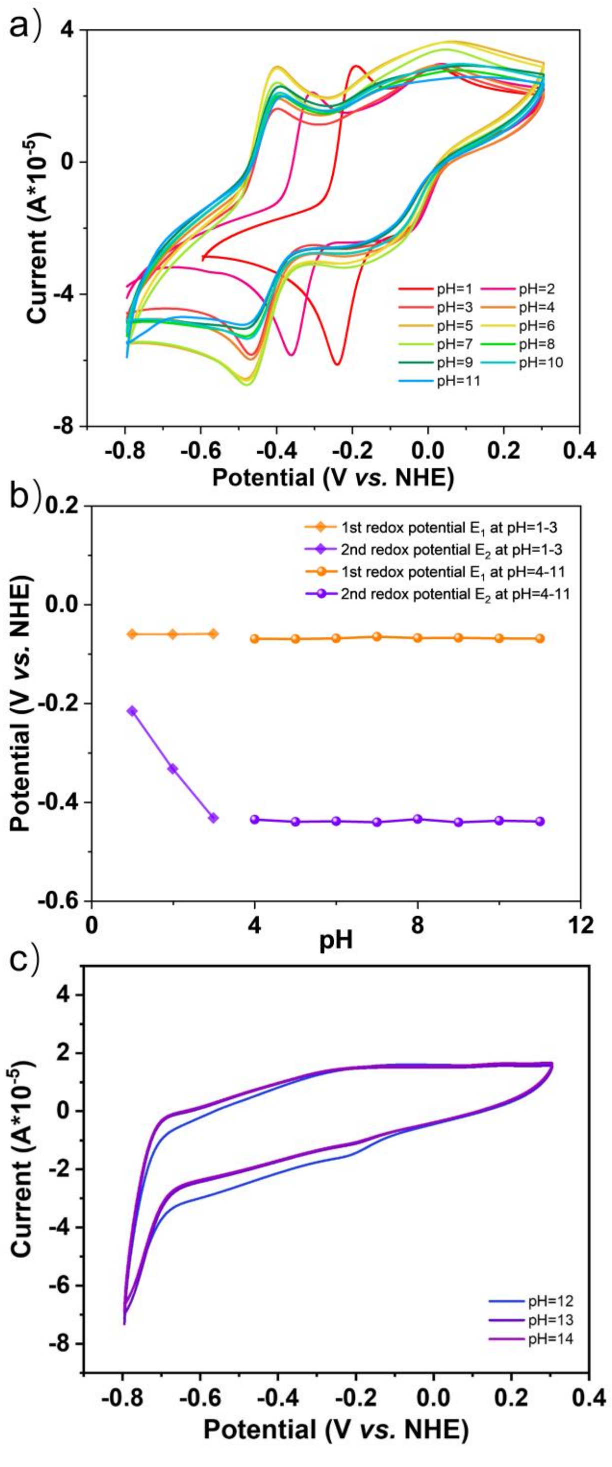

Figure S7. a) CV of **(CBu)$_2$NDI** from pH 1 to 11. Condition: 4 mM, 0.5 M KCl solution, 0.1 V s^{-1}. b) The corresponding Pourbaix diagrams of **(CBu)$_2$NDI**. c)CV curves of 4 mM **(CBu)$_2$NDI** at pH 12-14 in a 0.5 M KCl aqueous solution.

8. The electrochemical kinetics studies

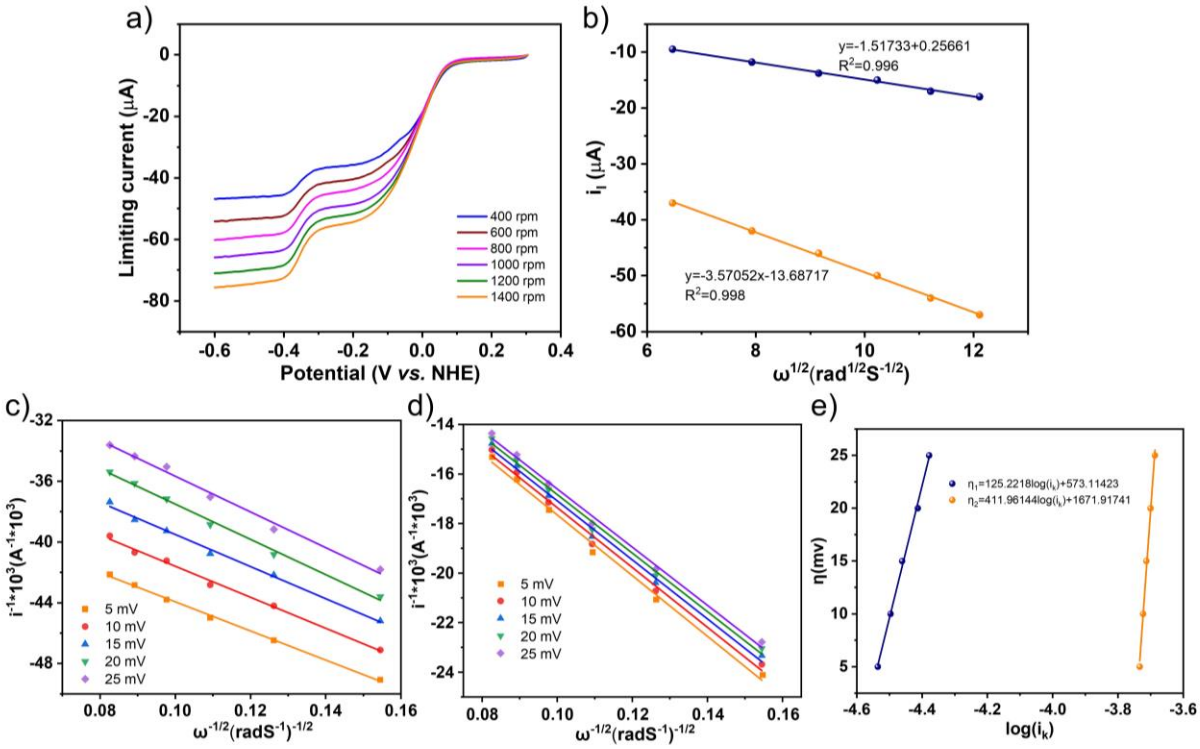

Figure S8. LSV measurements of 1 mM **$(SPr)_2$NDI** in 0.5 M KCl. a). The limiting current versus potential at rotation rates (400, 600, 800, 1000, 1200 rpm) and potential sweeping rate of 5 mV s^{-1}. b) Levich plot (i^{-1} *vs.* $\omega^{1/2}$) the of two-electron transfer process of **$(SPr)_2$NDI**. c) Koutecký-Levich plot (i^{-1} *vs.* $\omega^{-1/2}$) of first redox potential for **$(SPr)_2$NDI** at different overpotentials (5, 10, 15, 20, 25 mV), d) Koutecký-Levich plot (i^{-1} *vs.* $\omega^{-1/2}$) of second redox potential for **$(SPr)_2$NDI** at different overpotentials (5, 10, 15, 20, 25 mV). d) e) Tafel plot (η *vs.*logi_k) of the two-electron transfer process of **$(SPr)_2$NDI**.

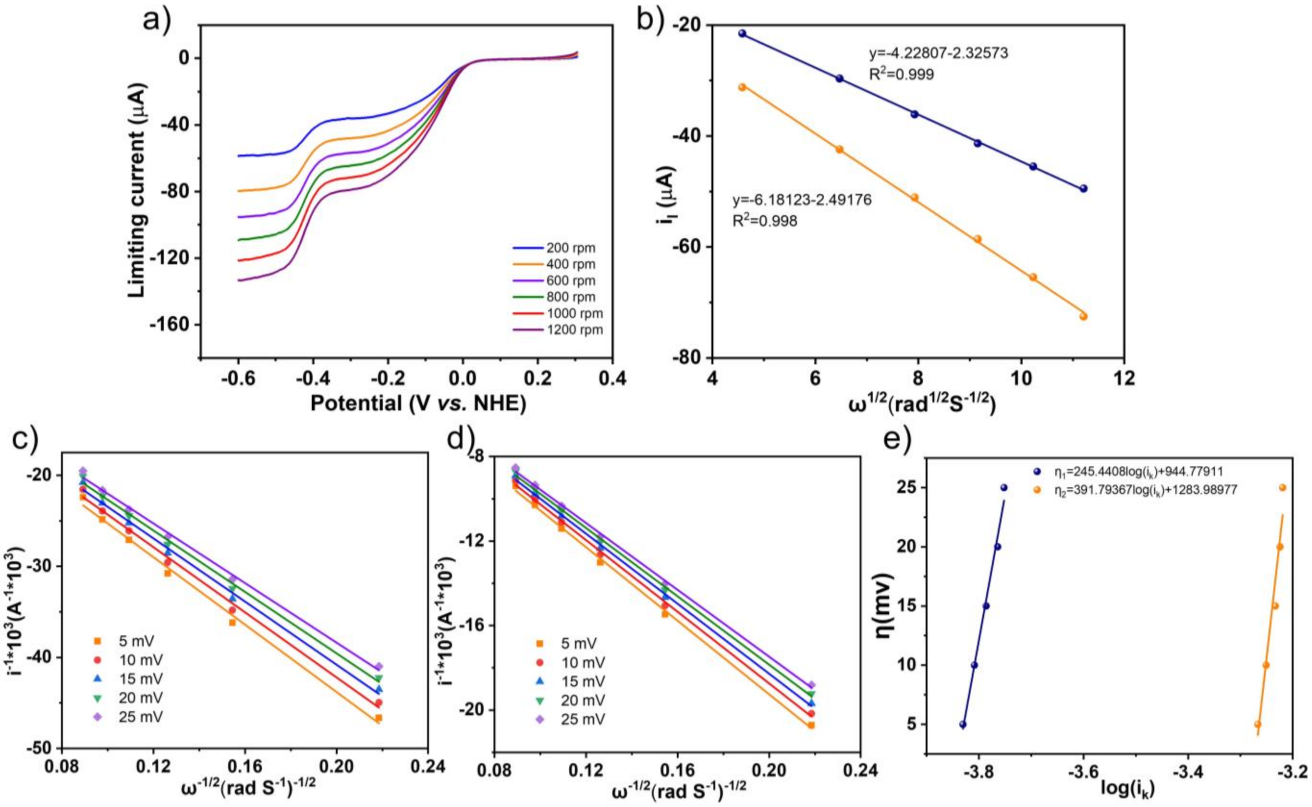

Figure S9. LSV measurements of 1 mM **(SPrOH)$_2$NDI** in 0.5 M KCl. a). The limiting current versus potential at rotation rates (200, 400, 600, 800, 1000, 1200 rpm) and potential sweeping rate of 5 mV s^{-1}. b) Levich plot (i^{-1} *vs.* $\omega^{1/2}$) the of two-electron transfer process of **(SPrOH)$_2$NDI**. c) Koutecký-Levich plot (i^{-1} *vs.* $\omega^{-1/2}$) of first redox potential for **(SPrOH)$_2$NDI** at different overpotentials (5, 10, 15, 20, 25 mV), d) Koutecký-Levich plot (i^{-1} vs. $\omega^{-1/2}$) of second redox potential for **(SPrOH)$_2$NDI** at different overpotentials (5, 10, 15, 20, 25 mV). d) e) Tafel plot (η *vs.*logi_k) of the two-electron transfer process of **(SPrOH)$_2$NDI**.

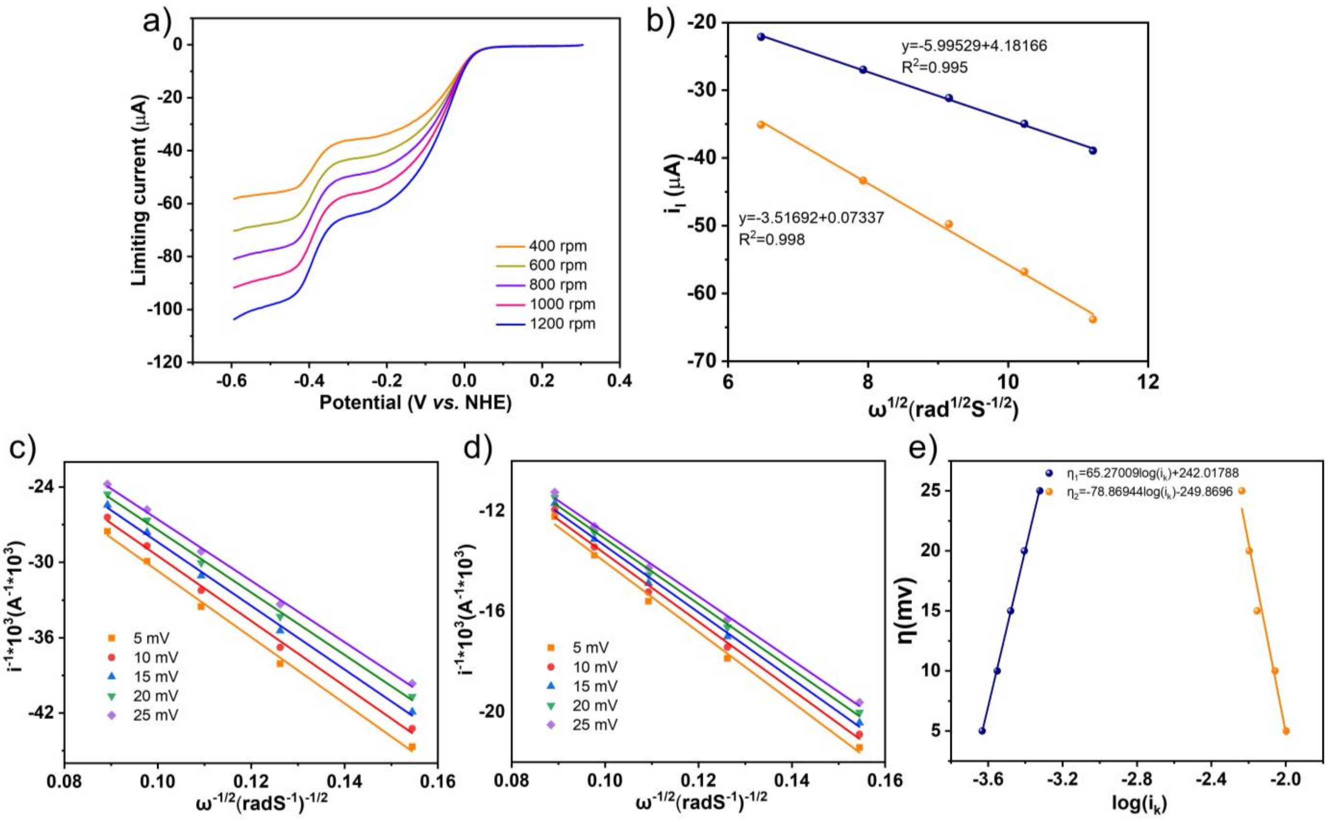

Figure S10. LSV measurements of 1 mM **(CBu)$_2$NDI** in 0.5 M KCl. a). The limiting current versus potential at rotation rates (200, 400, 600, 800, 1000, 1200 rpm) and potential sweeping rate of 5 mV s^{-1}. b) Levich plot (i^{-1} *vs.* $\omega^{1/2}$) the of two-electron transfer process of **(CBu)$_2$NDI**. c) Koutecký-Levich plot (i^{-1} *vs.* $\omega^{-1/2}$) of first redox potential for **(CBu)$_2$NDI** at different overpotentials (5, 10, 15, 20, 25 mV), d) Koutecký-Levich plot (i^{-1} *vs.* $\omega^{-1/2}$) of second redox potential for **(CBu)$_2$NDI** at different overpotentials (5, 10, 15, 20, 25 mV). d) e) Tafel plot (η *vs.*logi_k) of the two-electron transfer process of **(CBu)$_2$NDI**.

Table S1. Electrochemical data of **(SPr)$_2$NDI**, **(SPrOH)$_2$NDI,** and **(CBu)$_2$NDI**.

Compound	$E_{1/2}$(V *vs.* NHE)	Cell voltage (V) Paired with K_4FeCN_6	Diffusion coefficient (D, cm^2 s^{-1})	Electron transfer constant (k_0, cm s^{-1})
(SPr)$_2$NDI	-0.11, -0.43	-0.93	1.62×10^{-7}, 4.5×10^{-7}	1.40×10^{-3}, 4.61×10^{-3}
(SPrOH)$_2$NDI	-0.11, -0.44	-0.94	3.70×10^{-7}, 2.09×10^{-6}	7.46×10^{-3}, 2.77×10^{-2}
(CBu)$_2$NDI	-0.12, -0.44	-0.94	3.54×10^{-6}, 1.35×10^{-6}	1.03×10^{-2}, 3.58×10^{-2}

9. X-ray single crystal structural analyses

X-ray Crystallography. X-ray diffraction data collection of the compounds was recorded by Bruker VENTURE system with PHOTON II CPAD detector equipped at 296.15 K and a Ga-target Liquid Metal Source (λ = 1.34139 Å). The structures were solved by SHELXT (version 2018/2) and refined by full-matrix least-squares procedures using the SHELXL program (version 2018/3) through the OLEX2 graphical interface.

Method of single crystal growth: dissolve 10 mg of yellow **$(CBu)_2NDI$** powder in 5 ml of MeOH. After completely dissolving it by ultrasonication, filter the obtained solution through cotton into a 10 mL glass tube, seal the mouth with cling film, and then place it in a 100 mL glass bottle containing 20 ml of isopropyl ether solution. After covering the bottle, keep it at a constant temperature of around 25 degrees Celsius. Due to the diffusion effect, isopropyl ether evaporates slowly into the methanol solution. After three weeks, pale yellow needle-like **$(CBu)_2NDI$** crystals are obtained.**(CCDC: 2372397)**

Table 1. Crystal data and structure refinement for**$(CBu)_2NDI$**. (CCDC number: 2372397)

Empirical formula	$C_{32}H_{40}N_4O_8$
Formula weight	608.79
Temperature	193.00 K
Wavelength	1.34139 A
Crystal system, space group	Monoclinic, P21/c 1
Unit cell dimensions	a = 19.855(2) A alpha = 90 °C
b = 7.5862(9) A	beta = 108.252(4) °C.
c = 24.797(3) A	gamma = 90 °C.
Volume	3547.2(7) A^3
Z, Calculated density	4, 1.327 Mg/m^3
Absorption coefficient	0.534 mm^-1
F(000)	1520
Crystal size	0.13 x 0.1 x 0.08 mm
Theta range for data collection	5.468 to 57.277 °C.
Limiting indices	-24<=h<=23, -9<=k<=9, -30<=l<=24
Reflections collected / unique	25294 / 7205 [R(int) = 0.0769]
Completeness to theta = 53.594	99.40%
Absorption correction	Semi-empirical from equivalents
Max. and min. transmission	0.7512 and 0.6103
Refinement method	Full-matrix least-squares on F^2
Data / restraints / parameters	7205 / 2 / 462
Goodness-of-fit on F^2	1.025
Final R indices [I>2sigma(I)]	R1 = 0.0859, wR2 = 0.2471
R indices (all data)	R1 = 0.1552, wR2 = 0.2988
Extinction coefficient	n/a
Largest diff. peak and hole	0.386 and -0.501 e.A^-3

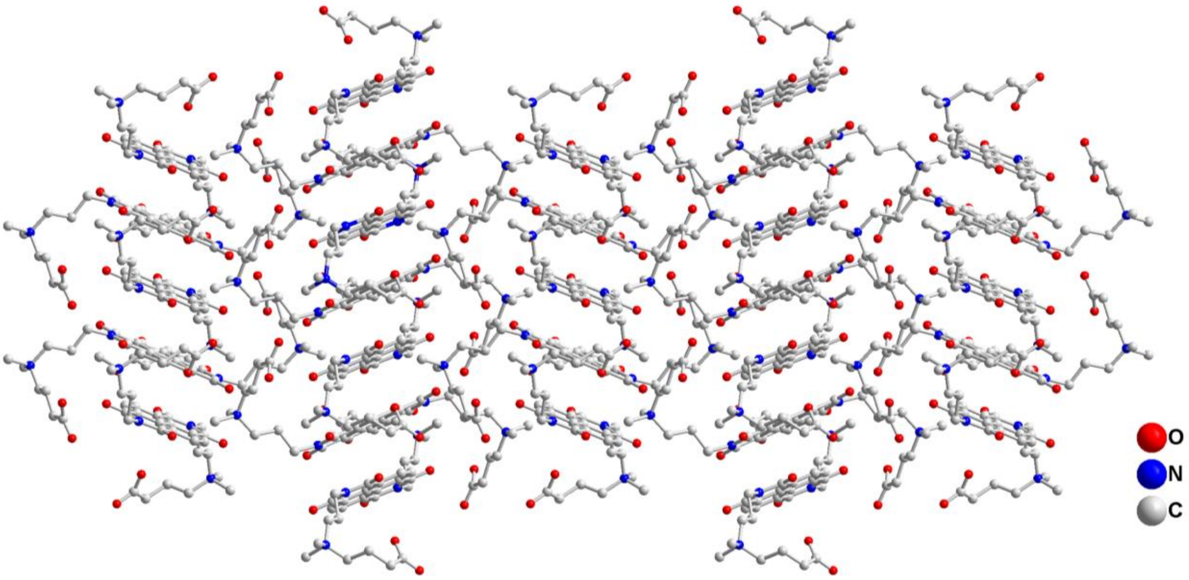

Figure S11. 3D frame perspective of **3** along the a-axis. Color code: C, grey; N, blue;H, cyan; and O, red.

10. Density functional theory (DFT) calculation.

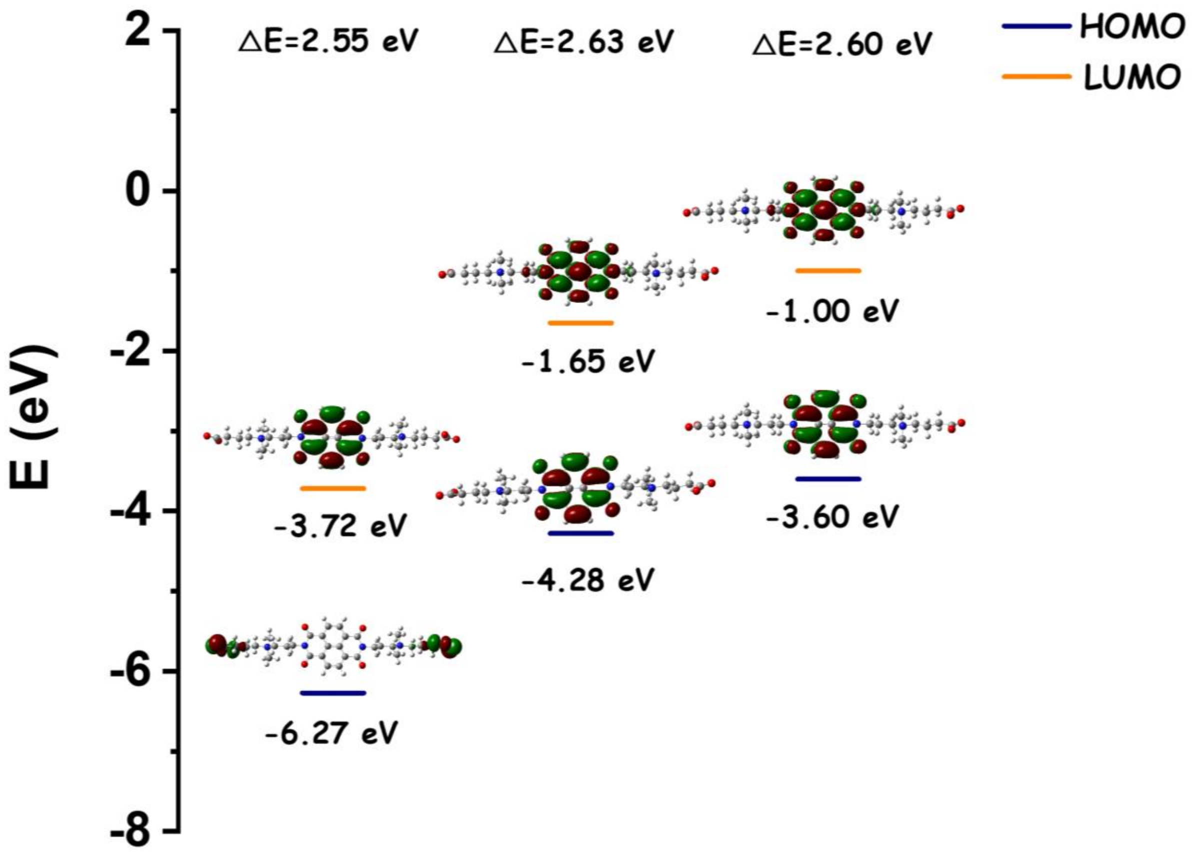

Figure S12. HOMO–LUMO plots and energy-level diagrams of **$(CBu)_2$NDI** at different redox states.

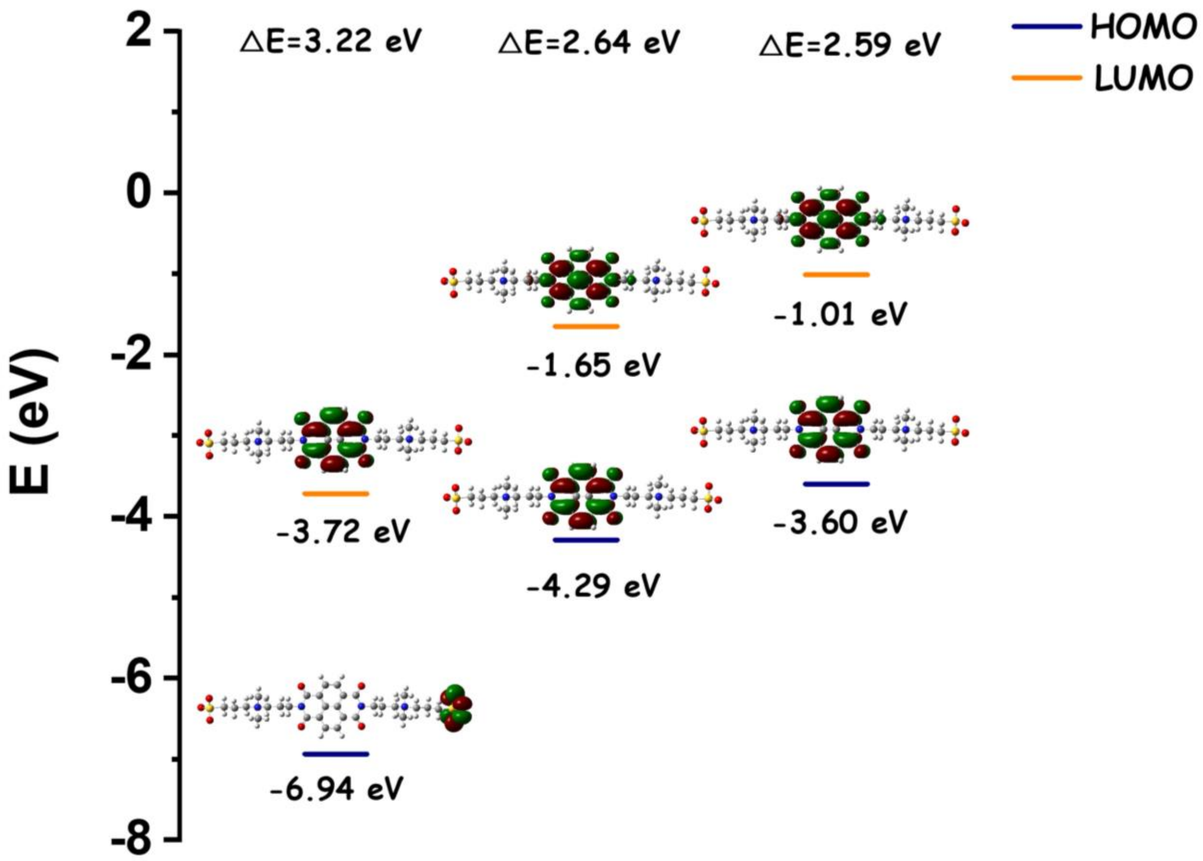

Figure S13. HOMO–LUMO plots and energy-level diagrams of **(SPr)$_2$NDI** at different redox states.

Table S2. Statistics of HOMO-LUMO and energy gap of **(CBu)$_2$NDI** and **(SPr)$_2$NDI**.

E (eV)	HOMO	LUMO	Energy gap (eV)
**(CBu)$_2$NDI**	-6.27	-3.72	2.55
**(CBu)$_2$NDI$^{1-\bullet}$**	-4.28	-1.65	2.63
**(CBu)$_2$NDI^{2-}**	-3.60	-1.00	2.60
**(SPr)$_2$NDI**	-6.94	-3.72	3.22
**(SPr)$_2$NDI$^{1-\bullet}$**	-4.29	-1.65	2.64
**(SPr)$_2$NDI^{2-}**	-3.60	-1.01	2.59

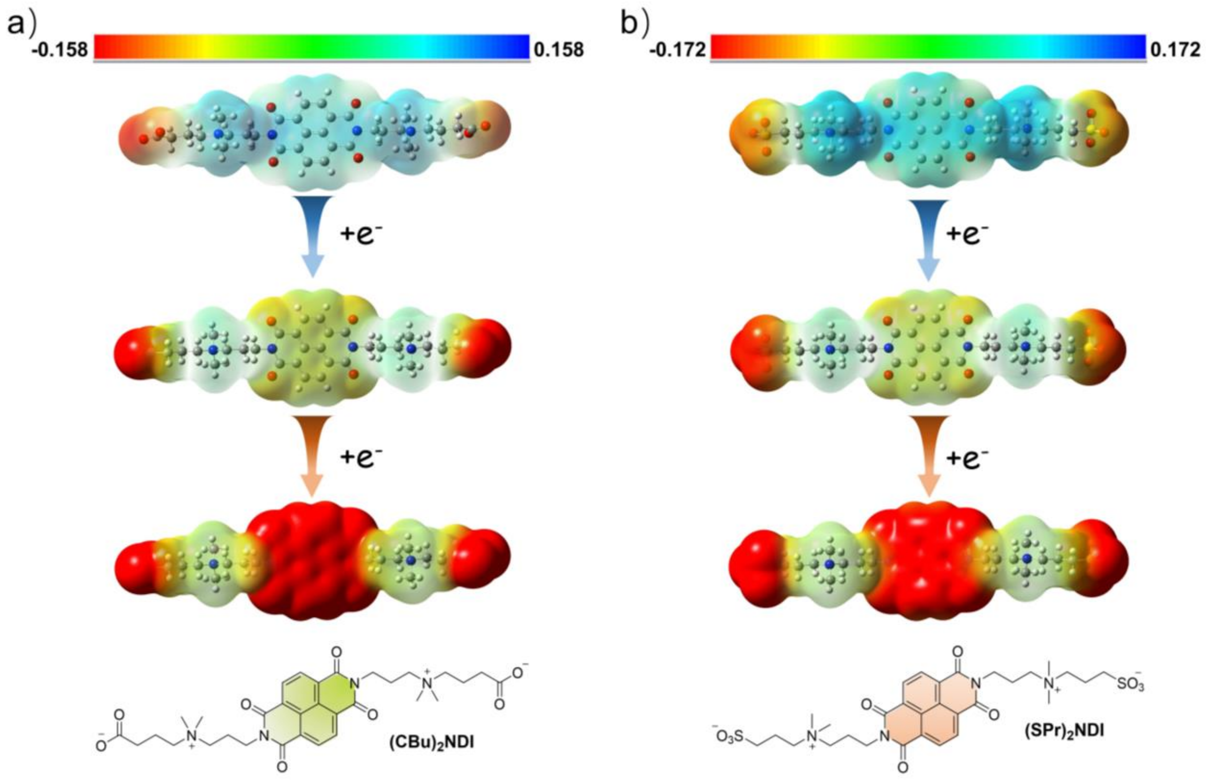

Figure S14. Electrostatic potential surfaces of a) **$(CBu)_2$NDI** and b) **$(SPr)_2$NDI** at three different states.

11. The spin density calculation.

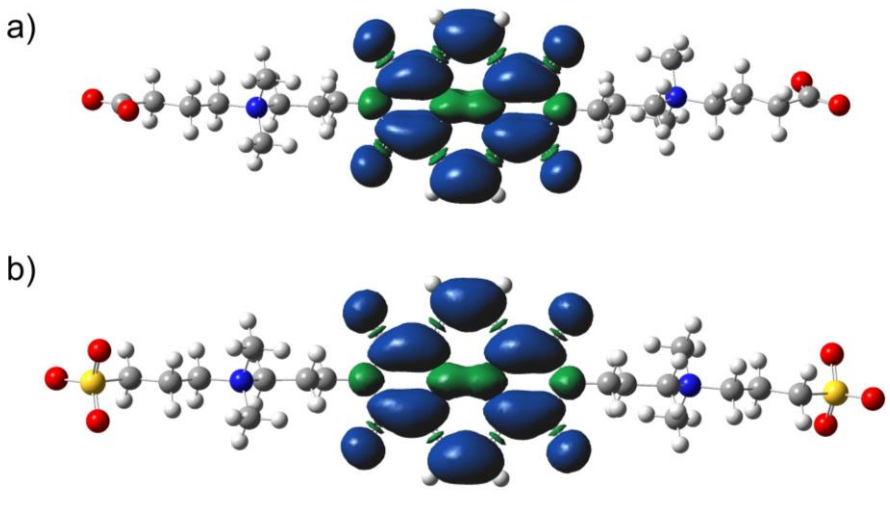

Figure S15. Spin density distribution of **$(CBu)_2NDI^{1-\bullet}$** and **$(SPr)_2NDI^{1-\bullet}$**.

12. Materials Studio.

The crystal structure prediction and powder X-ray diffraction pattern simulation were carried out using Materials Studio 2019.

<table>
<tr><th>Spatial type</th><th>Spatial structure</th><th>Diffraction peak comparison</th></tr>
<tr><td>C2</td><td>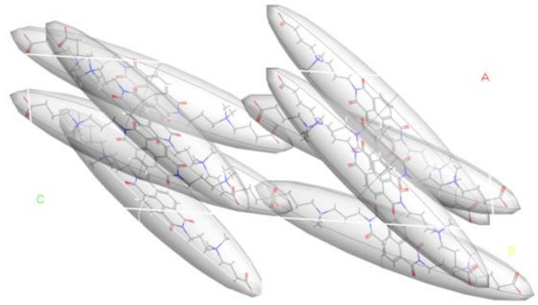</td><td>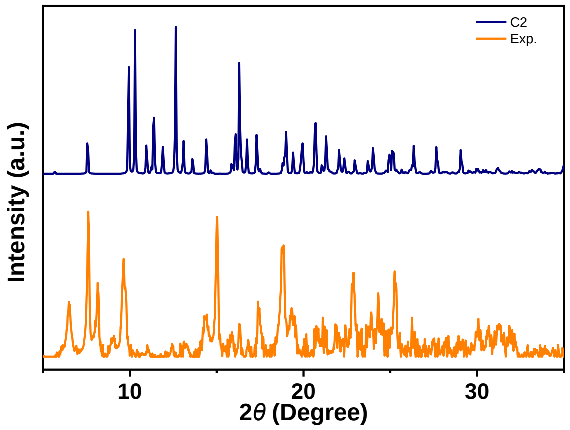
</td></tr>
<tr><td>C2-C</td><td>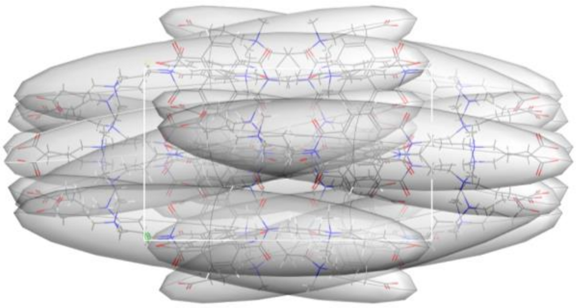</td><td>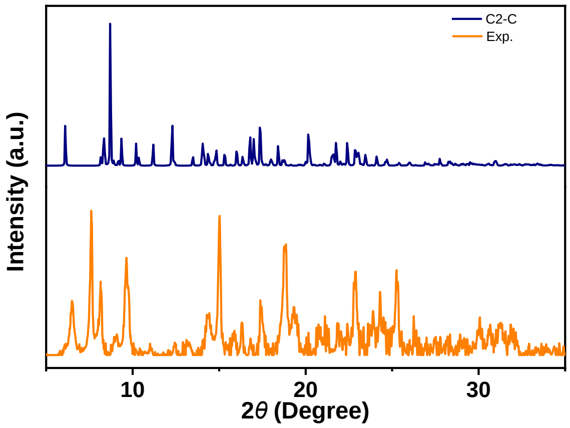
</td></tr>
<tr><td>CC</td><td>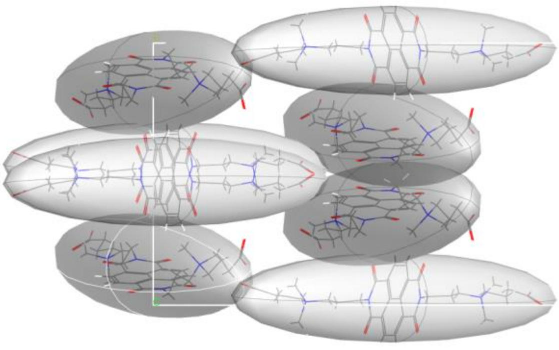</td><td>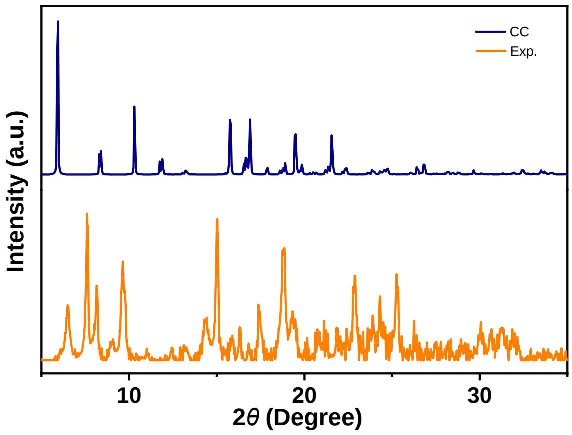
</td></tr>
</table>

P1	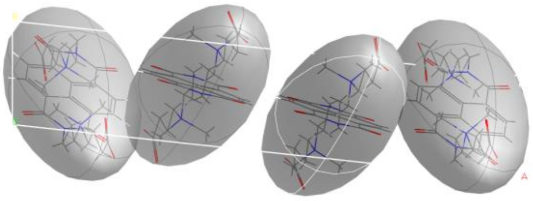	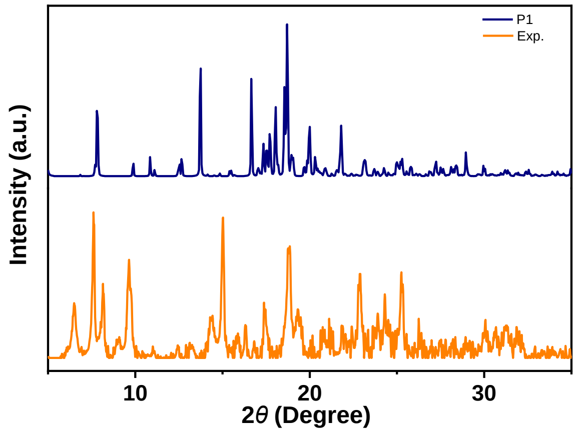 P1 Exp. Intensity (a.u.) 10 20 30 2θ (Degree)
P21-C	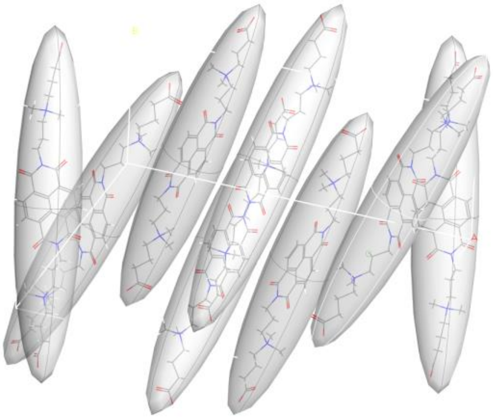	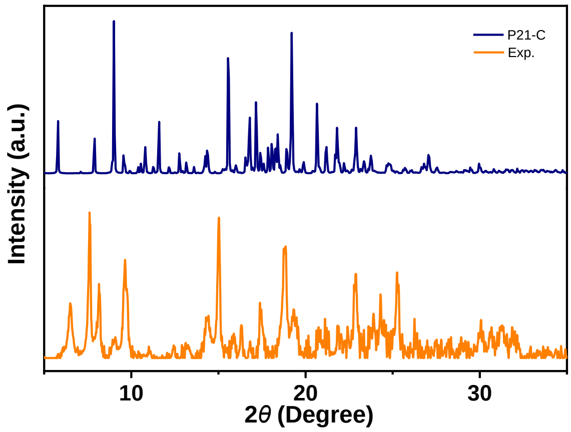 P21-C Exp. Intensity (a.u.) 10 20 30 2θ (Degree)
P21	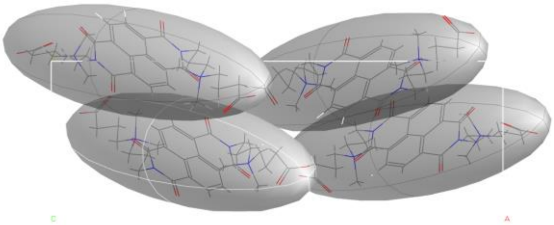	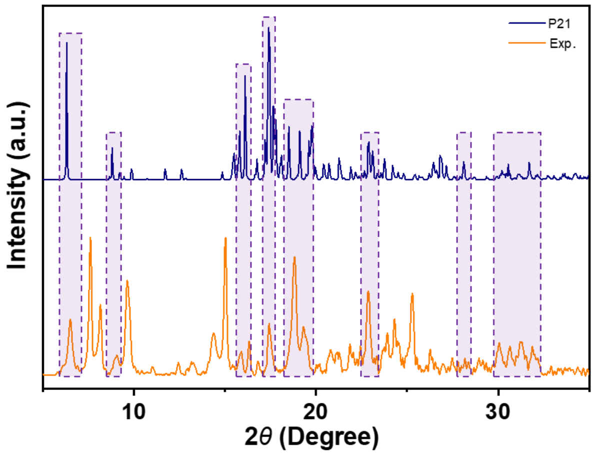 P21 Exp. Intensity (a.u.) 10 20 30 2θ (Degree)

<table>
<tr>
<td>P21212
1</td>
<td>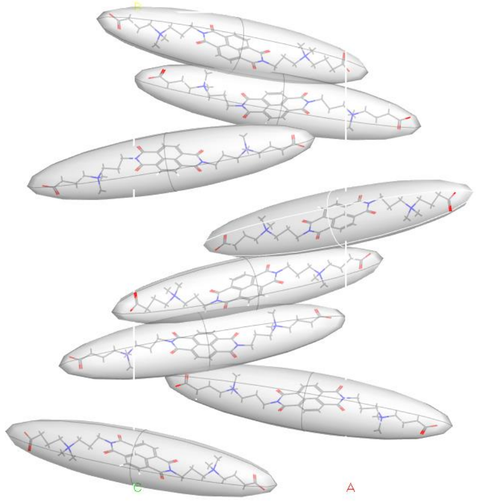</td>
<td>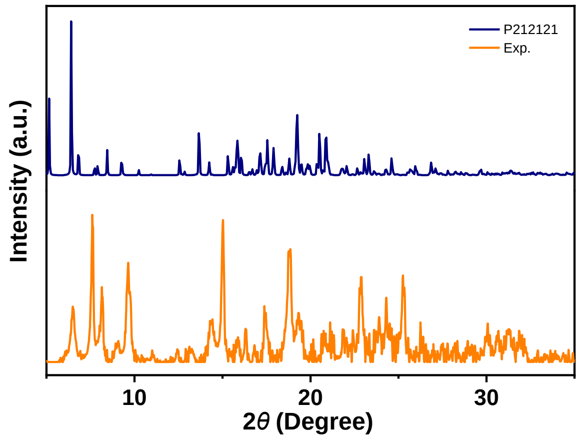
</td>
</tr>
<tr>
<td>PBCA</td>
<td>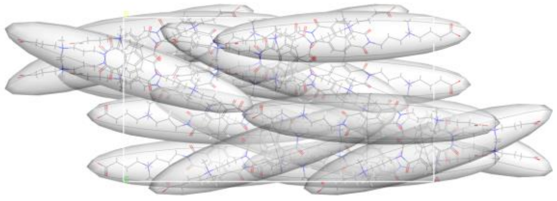</td>
<td>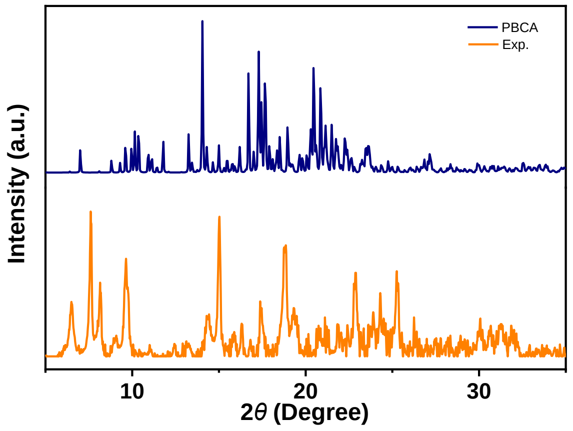
</td>
</tr>
<tr>
<td>PBCN</td>
<td>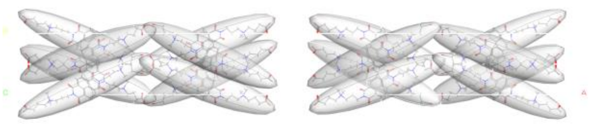</td>
<td>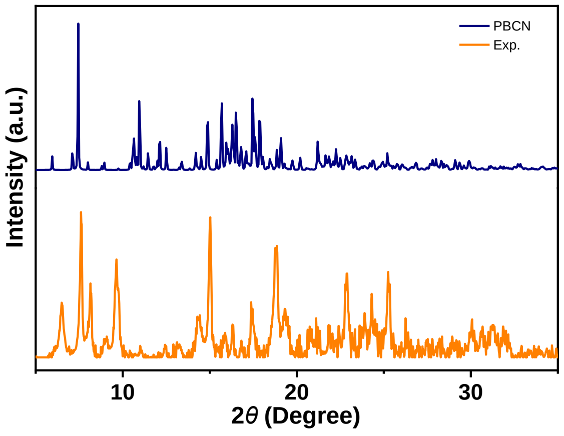
</td>
</tr>
</table>

PNA21	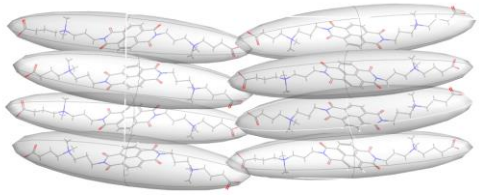	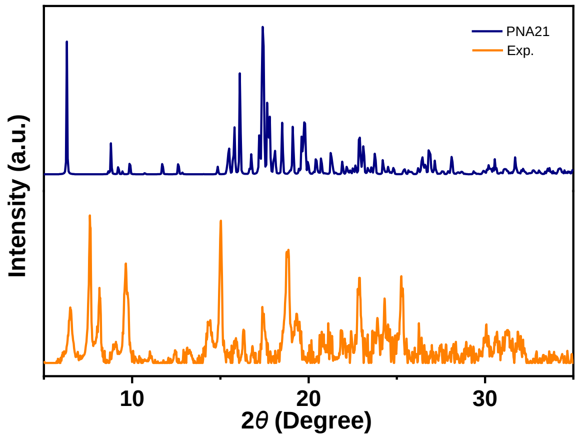

Figure S16. The XRD patterns of $(CBu)_2NDI$, experience (blue) and simulation (orange). Stacked structures of the **$(CBu)_2NDI$** simulated under ten possible crystal space groups, including C2, C2-C, CC,P1, P21-C, P21, P212121, PBCA, PBCN, PNA21.

13. Single point energy

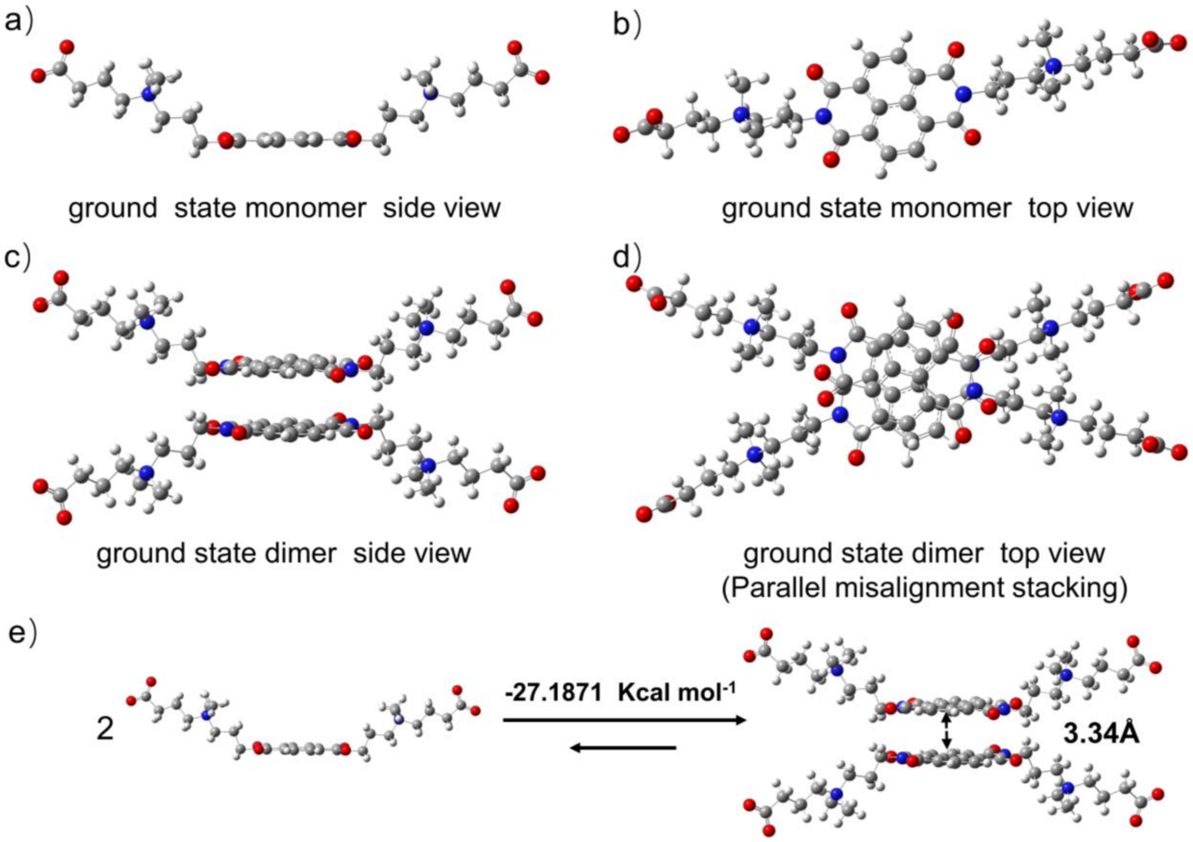

Figure S17. Ground state optimized structure in aqueous solution: a) monomer side view, b) monomer top view, c) dimer side view, d) dimer top view, e) monomer and dimer single point energy calculation and more stable existing structure.

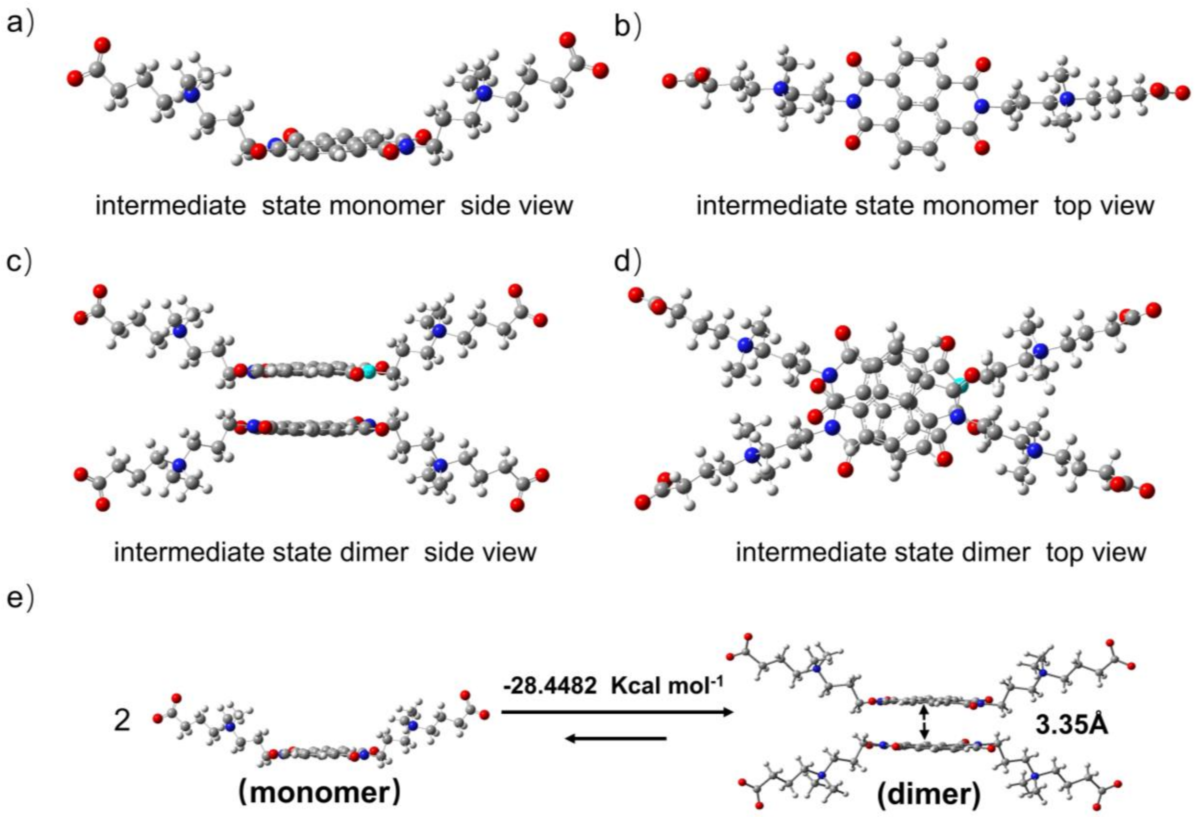

Figure S18. Intermediate state optimized structure in aqueous solution: a) monomer side view, b) monomer top view, c) dimer side view, d) dimer top view, e) monomer and dimer single point energy calculation and more stable existing structure.

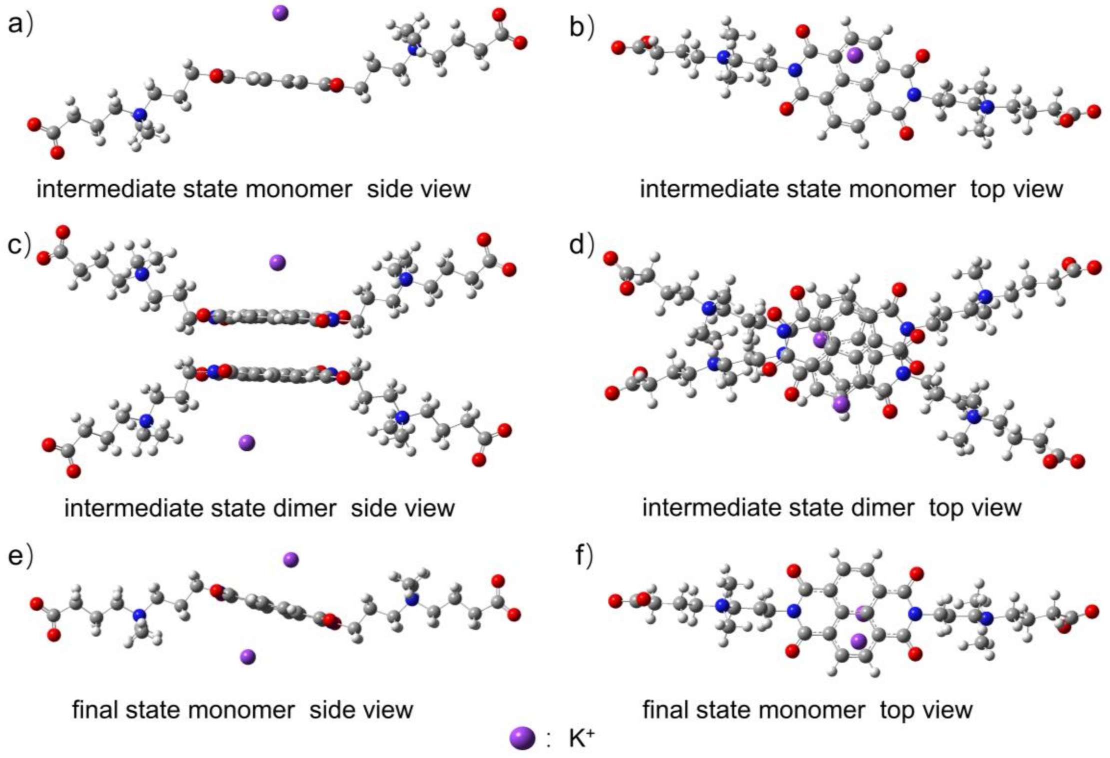

Figure S19. Optimized structures for electrostatic interactions between molecules and potassium ions in KCl solution: a) side view of an intermediate monomer interacting with one potassium ion, b) top view, c) side view of an intermediate dimer interacting with two potassium ions, d) top view. e) Side view of the terminal monomer interacting with two potassium ions, f) top view.

14. Electrostatic potentials and interactions in the presence of K^+

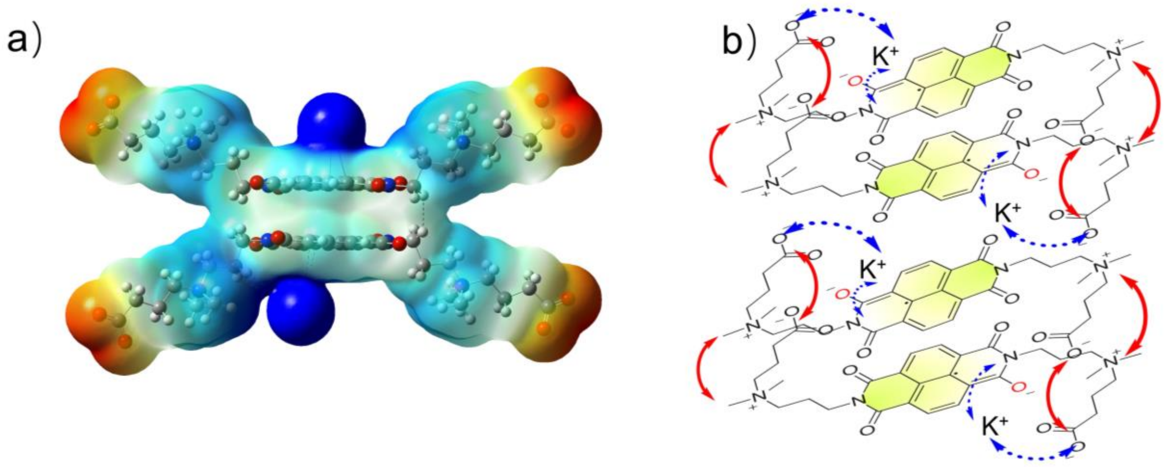

Figure S20. a) The electrostatic potential of the optimal configuration of the intermediate state. b) Schematic diagram of the interaction between molecules and the charge buffering effect of potassium ions (the solid red arrow represents electrostatic repulsion, and the dashed blue arrow represents electrostatic attraction).

15. Decomposition resistance test of $(CBu)_2$NDI

Table S3. pH at different Na_2CO_3 solutions.

Amounts / Samples	0 (μL)	1 (μL)	2 (μL)	3 (μL)	4 (μL)	5 (μL)	10 (μL)	20 (μL)
**pH of dex-NDI**	6.69	7.92	8.50	8.87	8.92	9.04	9.39	9.58
**pH of $(CBu)_2$NDI**	6.92	8.24	8.94	9.13	9.32	9.40	9.86	10.17

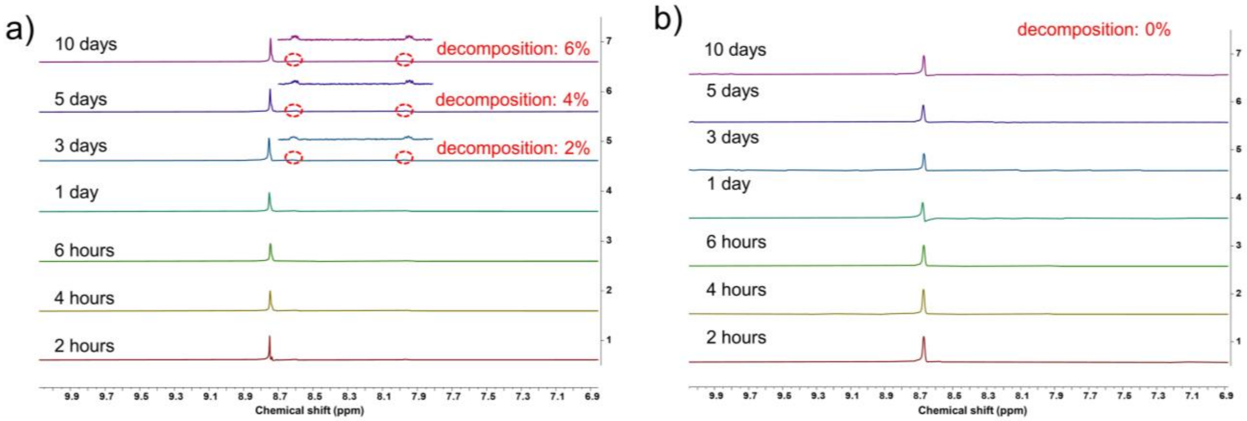

Figure S21. ^{1}H NMR spectra of 0.01 M (a) dex-NDI and (b) **$(CBu)_2$NDI** at 1 μL 0.1 M Na_2CO_3 amount.

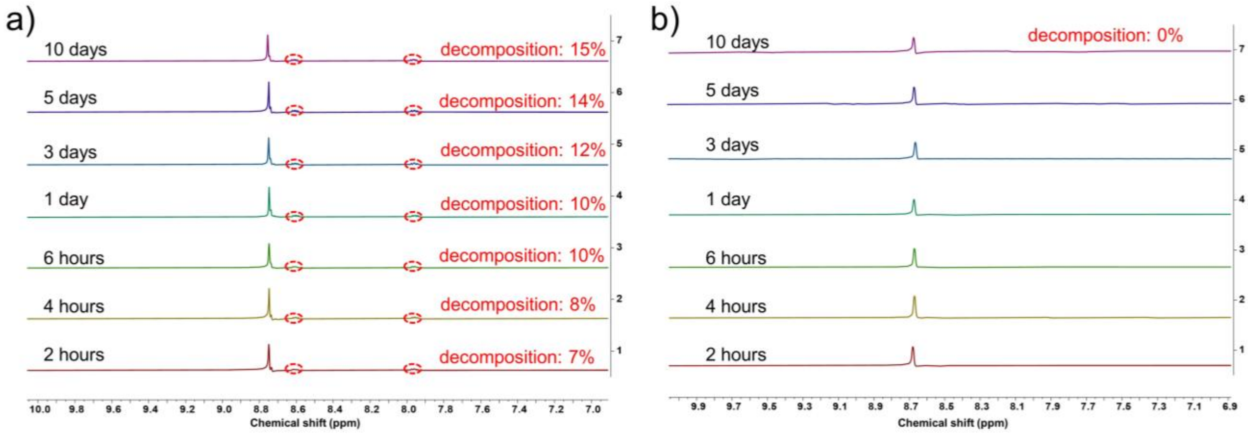

Figure S22. ^{1}H NMR spectra of 0.01 M (a) dex-NDI and (b) **$(CBu)_2$NDI** at 2 μL 0.1 M Na_2CO_3 amount.

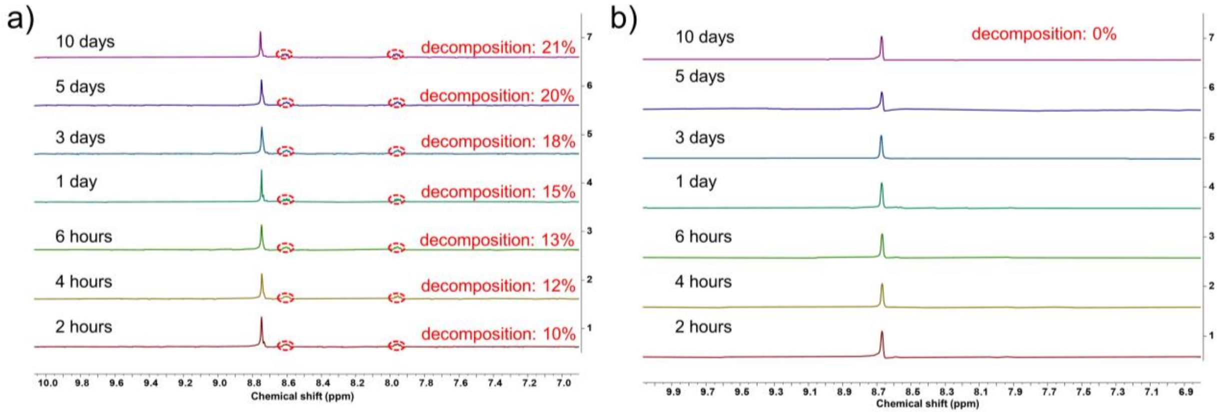

Figure S23. ^{1}H NMR spectra of 0.01 M (a) dex-NDI and (b) **$(CBu)_2$NDI** at 3 μL 0.1 M Na_2CO_3 amount.

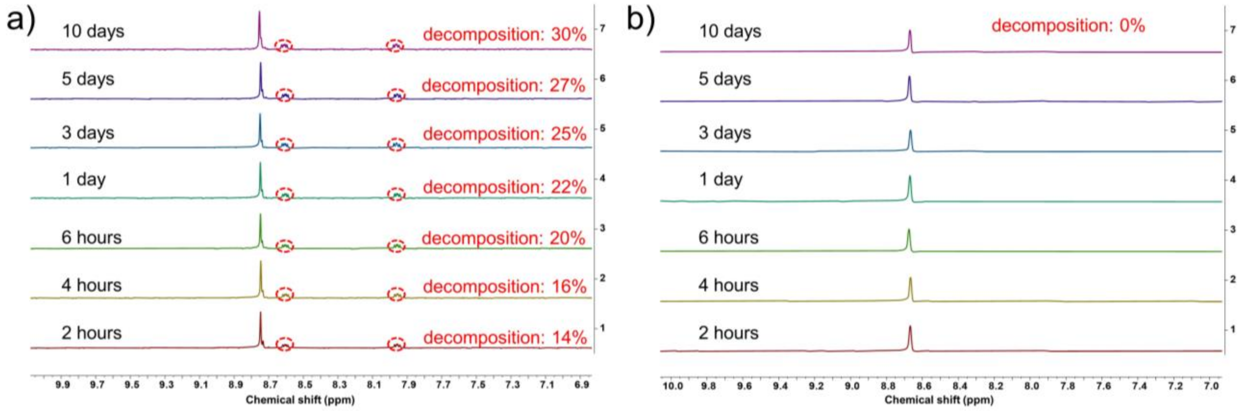

Figure S24. ^{1}H NMR spectra of 0.01 M (a) dex-NDI and (b) **$(CBu)_2$NDI** at 4 μL 0.1 M Na_2CO_3 amount.

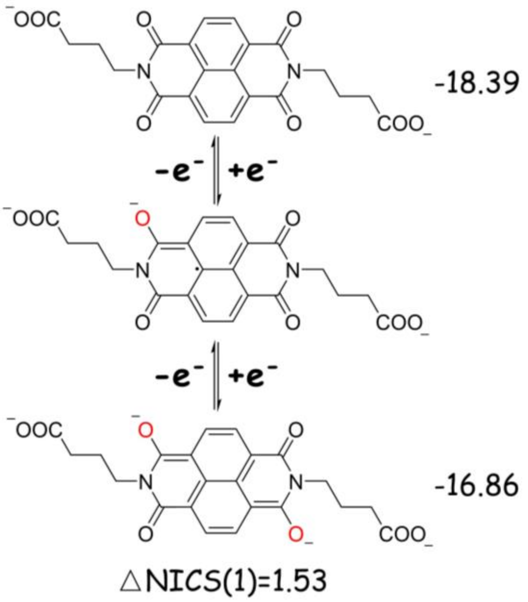

Figure S25. The NICS(1) values of K_2NDI.

16. Change in pH during charging

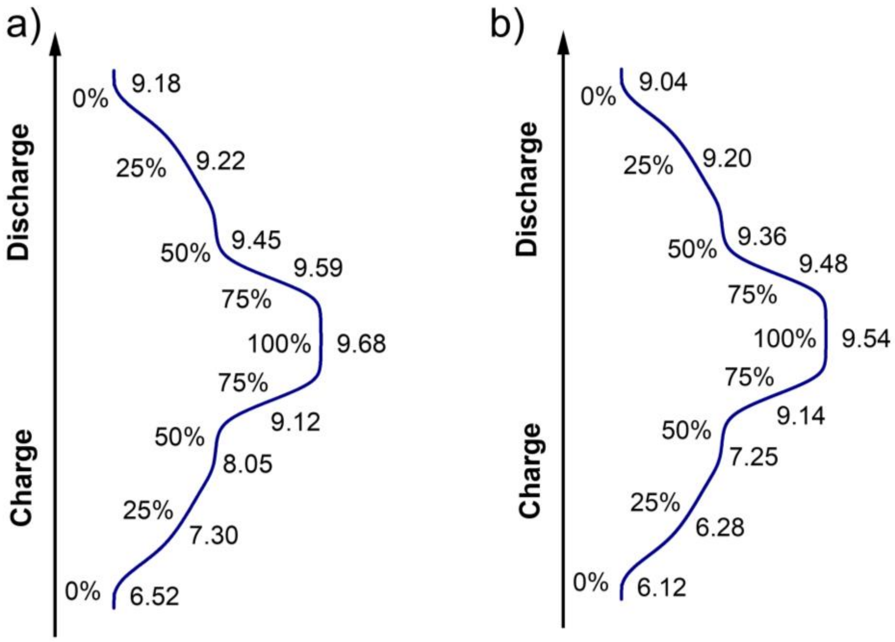

Figure S26. Changes in pH of a)$(CBu)_2$NDI and b)$(SPr)_2$NDI solution during charging and discharging.

17. HRMS of decomposition products

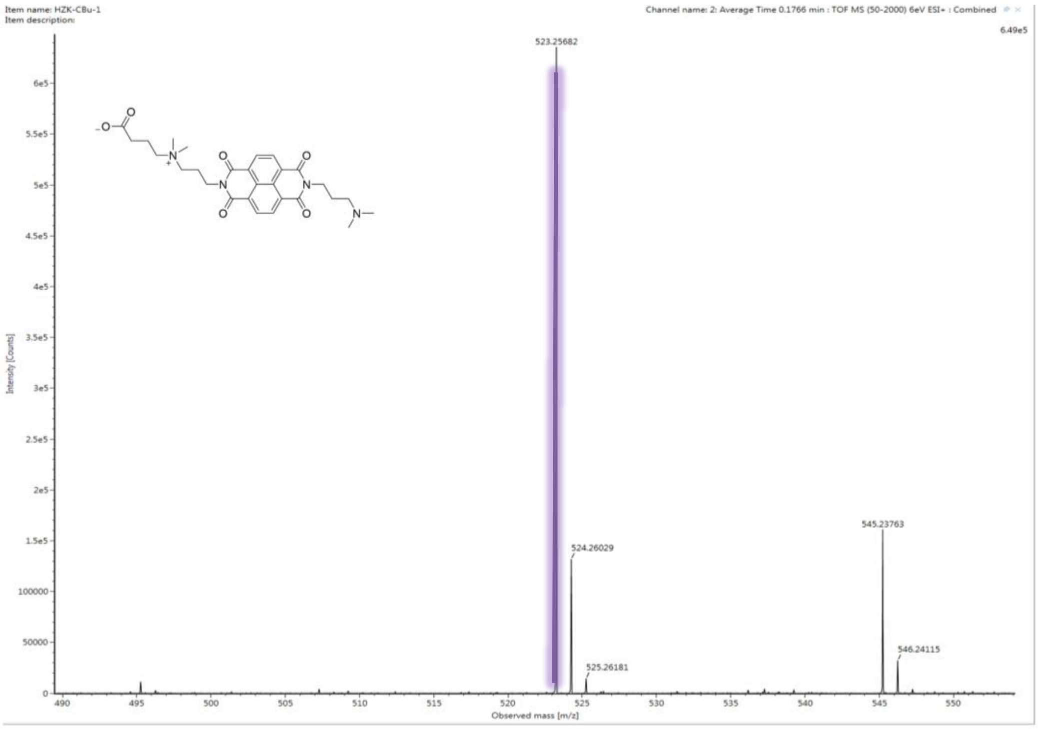

Figure S27. The HRMS spectra of $(CBu)_2NDI$ (One side). HRMS (ESI) m/z: [CBuNDI] calcd for C28H34N4O6 523.25511; found 523.25682.

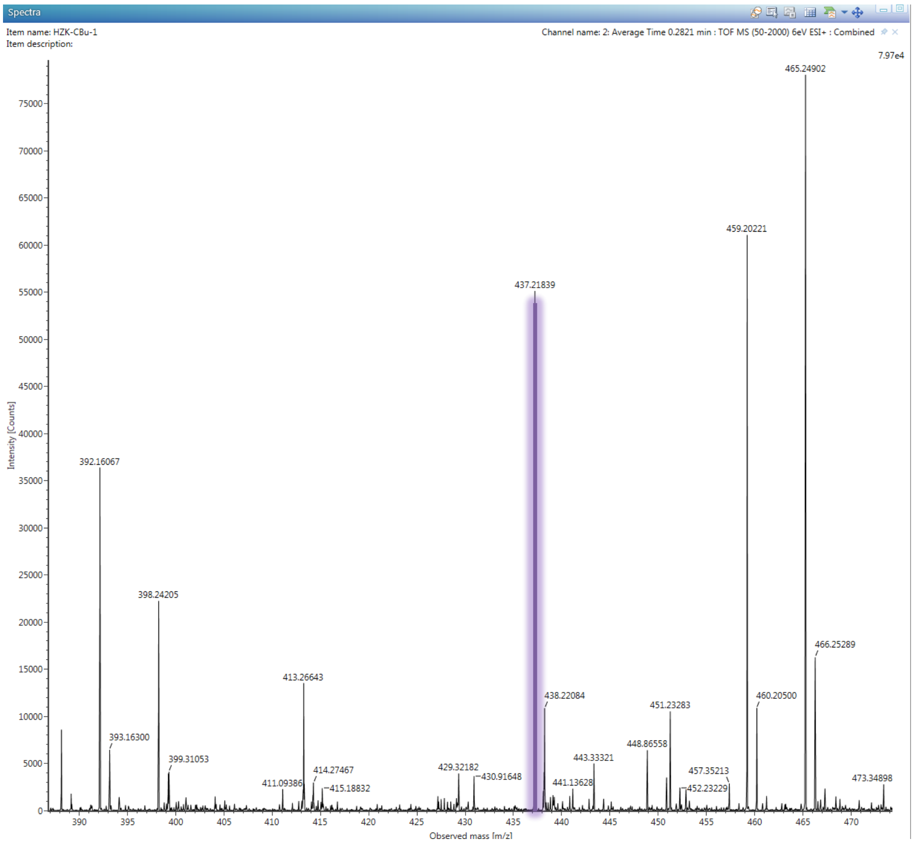

Figure S28. The HRMS spectra of NDI. HRMS (ESI) m/z: [NDI] calcd for C24H28N4O4 437.21833; found 437.21839.

18. Hydrogen bond interaction

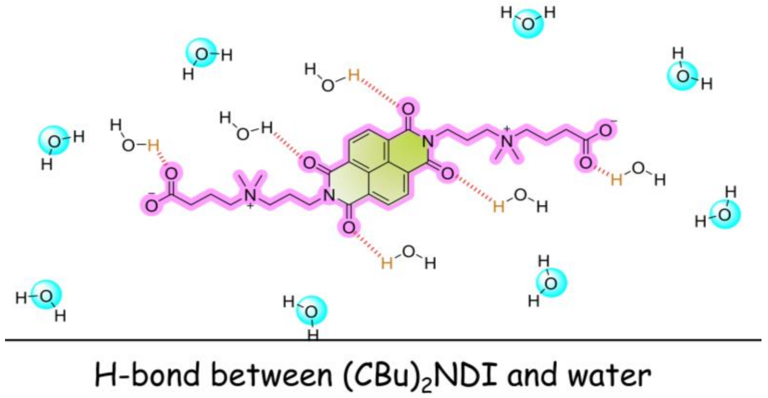

Figure S29. Schematic diagram of $(CBu)_2$NDI hydrogen bonding with water.

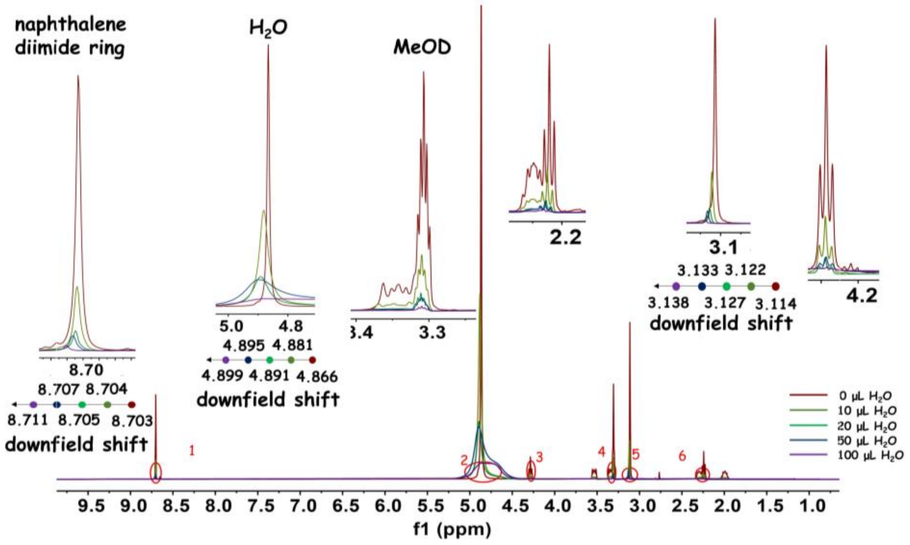

Figure S30. The ^{1}H NMR spectra of **$(CBu)_2$NDI** in MeOD solution at different water contents.

19. Configuration changes

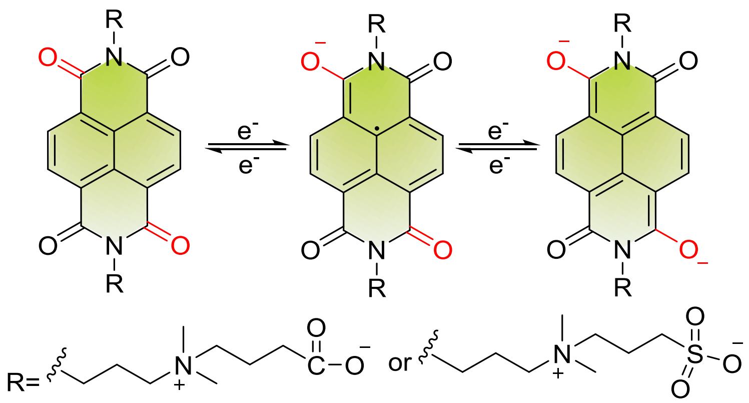

Figure S31. Configuration changes during the process of gaining and losing electrons of naphthalene diimide

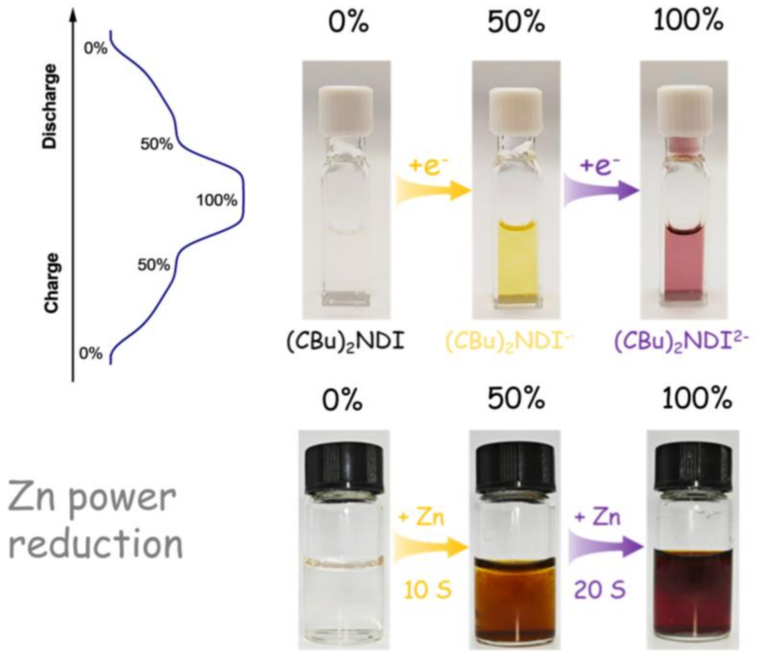

Figure S32. Color changes during electrochemical reduction (above) and chemical reduction (below) of **$(CBu)_2$NDI**

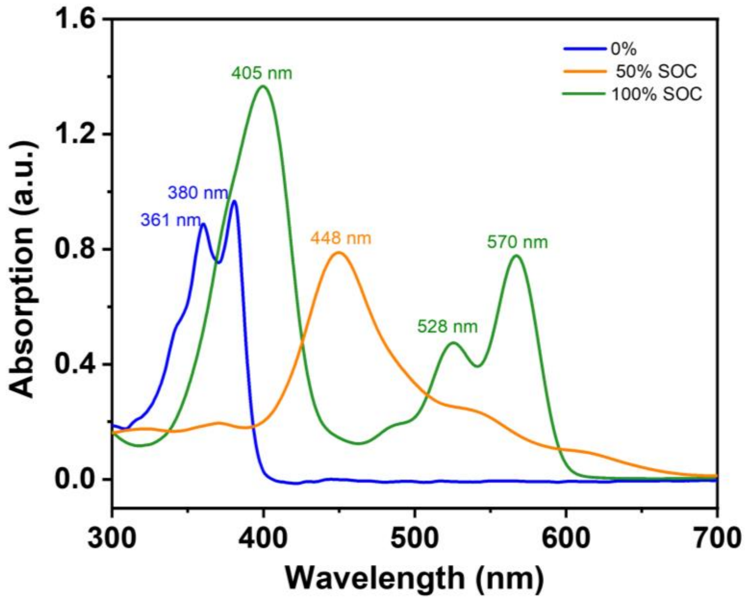

Figure S33. UV/Vis absorption spectra in three charging states.

20. EPR test

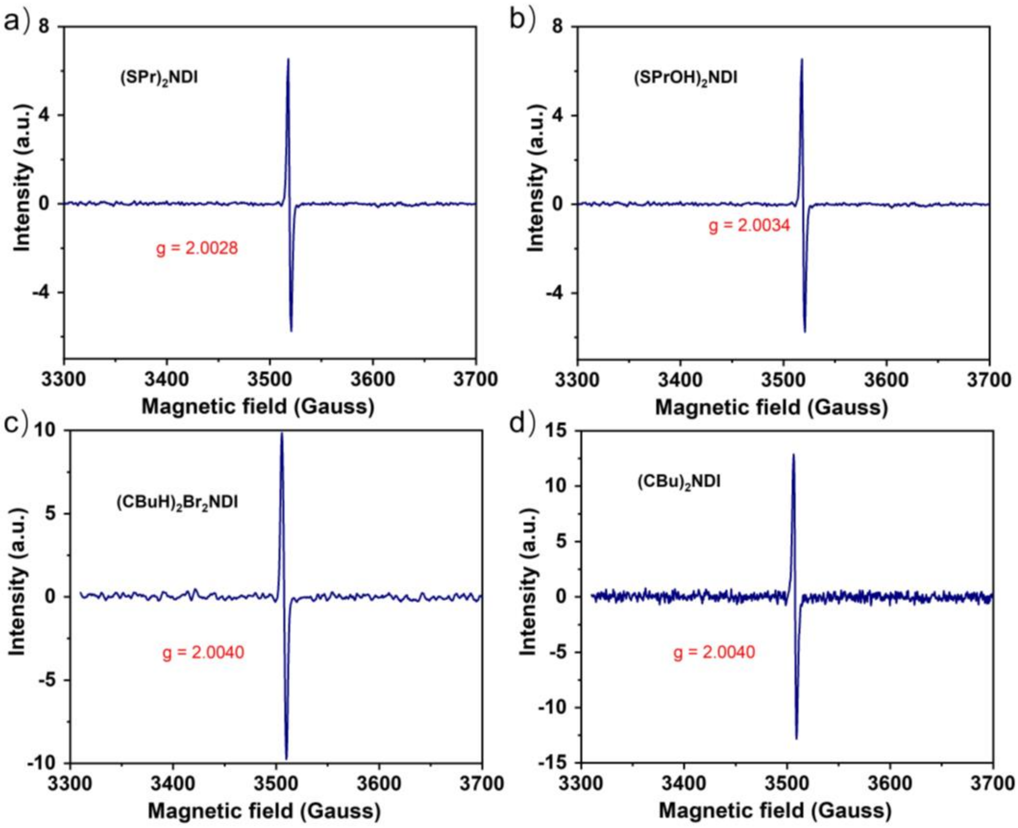

Figure S34. EPR of four molecules was measured in deionized water. a) **$(SPr)_2NDI$**, b) **$(SPrOH)_2NDI$**, c) **$[(CBu)_2NDIH_2]Br_2$** and d) **$(CBu)_2NDI$.**

21. Computed UV/Vis spectra

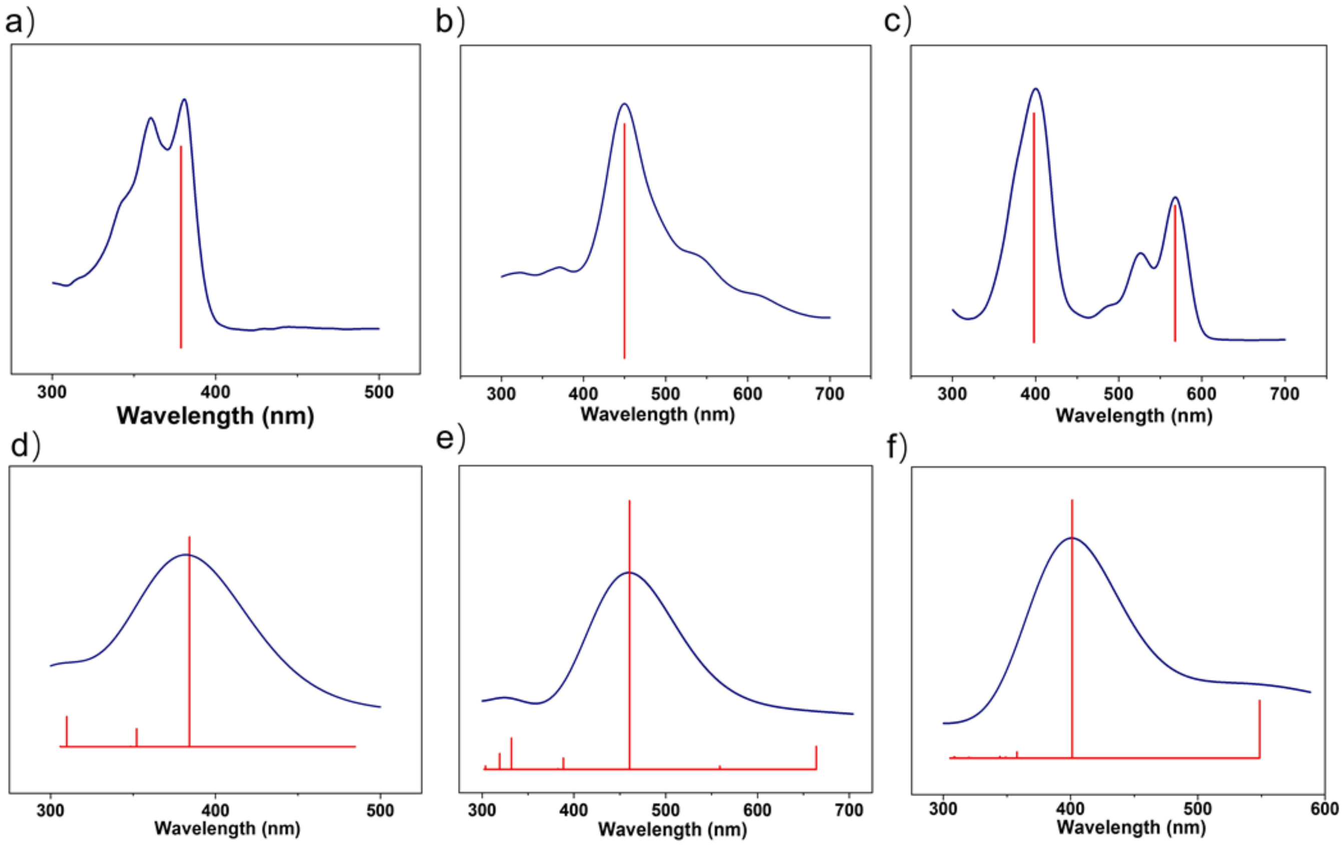

Figure S35. Experimental UV/Vis spectra in the H_2O of **$(CBu)_2$NDI** at a) ground state, b) after gaining one electron c) after gaining two electrons and Computed, at the TD-B3LYP/6-311+G(d) level of theory at a) ground state, b) after gaining one electron c) after gaining two electrons.

22. In situ UV/Vis spectra

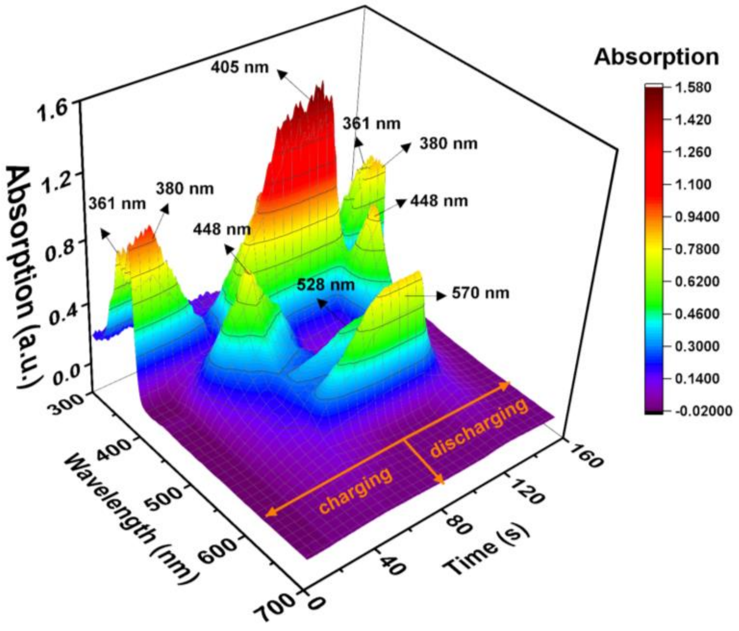

Figure S36. In situ UV/Vis absorption spectra during charging and discharging of **(CBu)$_2$NDI**.

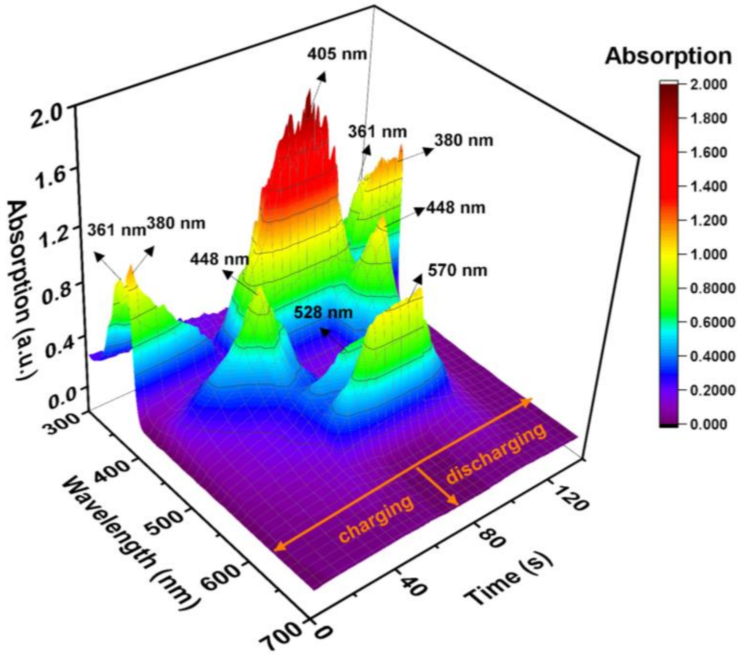

Figure S37. In situ UV/Vis absorption spectra during charging and discharging of **(SPr)$_2$NDI**.

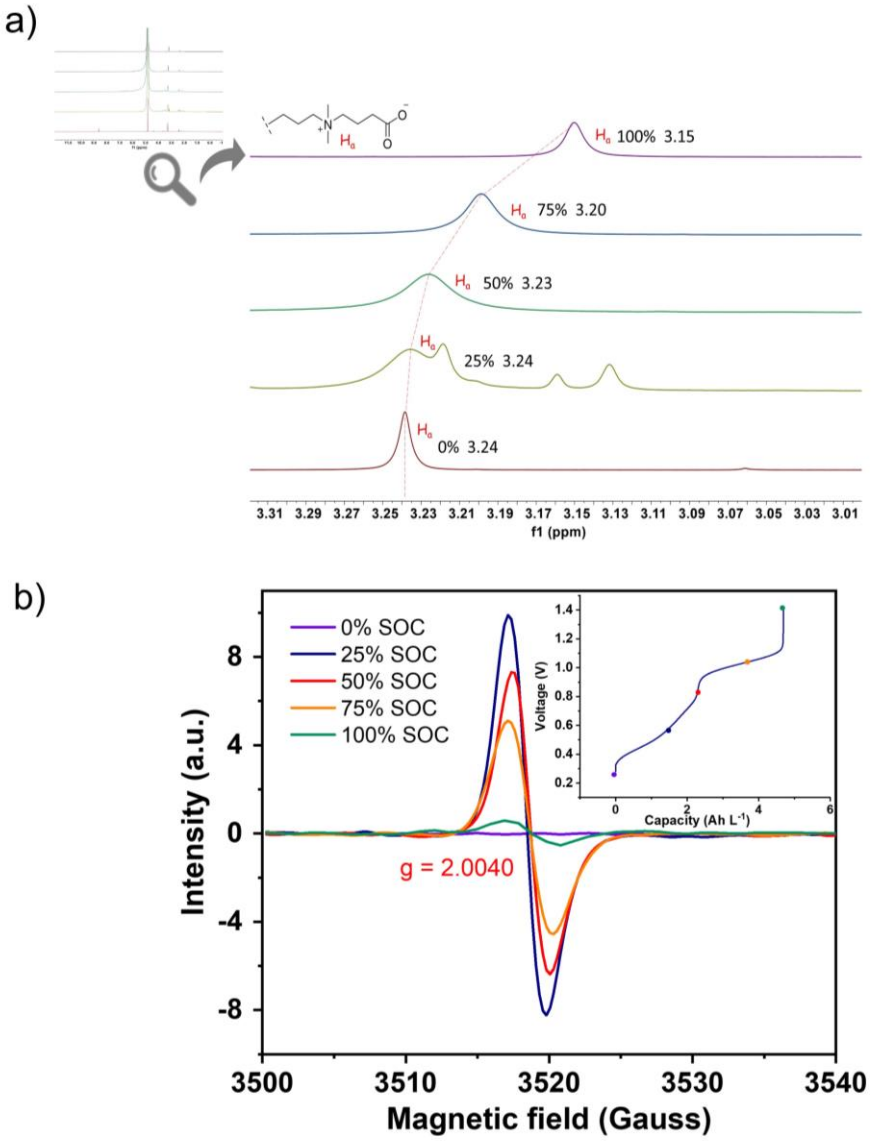

Figure S38. a) Full ^{1}H NMR (400 MHz) spectra of **(CBu)$_2$NDI** in a KCl solution throughout 0~100% SOC. The disappearance of Hc (~8.6 ppm, aromatic-ring part) at 25~100% SOC is attributed to the formation of radicals. b) EPR responses of 0.1 M **(CBu)$_2$NDI** anolyte at different charge states, corresponding to the coloured dots of the charge curve in inset.

23. ^{1}H NMR and UV/Vis characterization of $(CBu)_2NDI$ before cycling

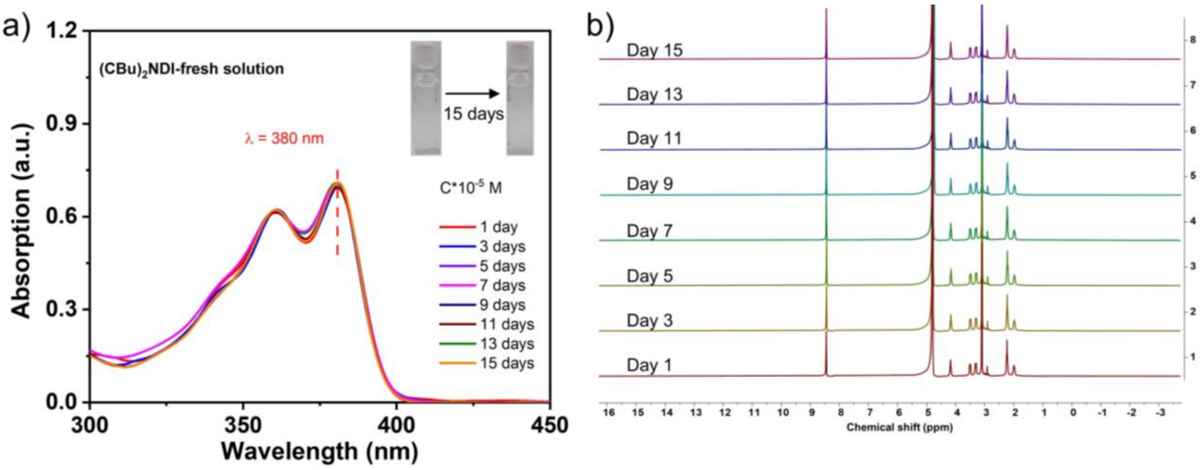

Figure S39. a) The ^{1}H NMR spectra of **$(CBu)_2NDI$** during 15 days. b) The UV absorption change of **$(CBu)_2NDI$** during 15 days.

24. ^{1}H NMR characterization of $(CBu)_2NDI$ in cycling

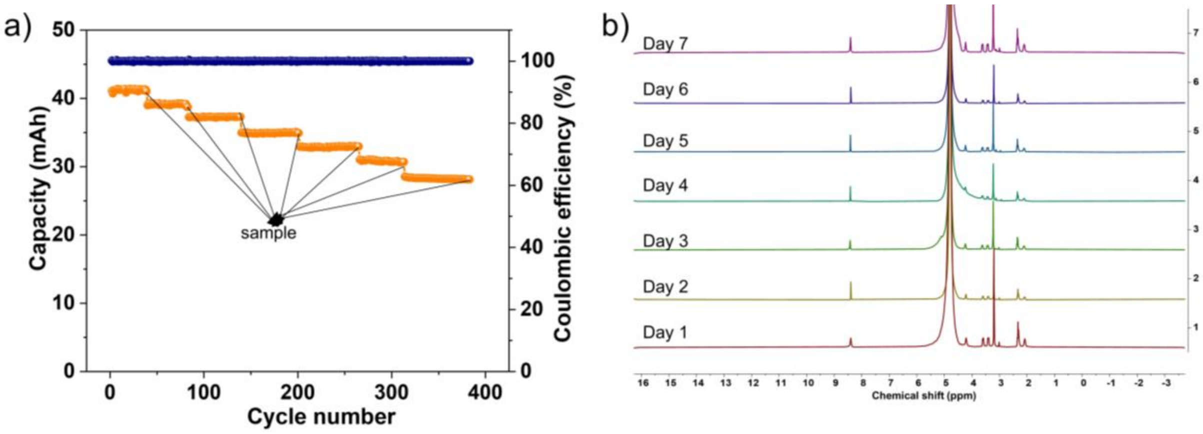

Figure S40. a) The cycle data of 0.1 M **$(CBu)_2NDI/K_4Fe(CN)_6$**-based AORFB. b) The ^{1}H NMR spectra analysis of 0.1 M **$(CBu)_2NDI$** in cycling for 7 days. It is worth noting that, **$(CBu)_2NDI$** was pre-dissolved in 10 mL of D_2O for convenience in sampling. Samples were taken every other day, and as the volume of the solution gradually decreased, the battery capacity also correspondingly declined.

25. Treatment of ion-exchange membranes

The pretreatment process for commercially acquired Nafion 212 membranes aims to convert its conductive ions and optimize its performance. The specific steps are as follows: Firstly, the Nafion 212 membrane is immersed in a 5% hydrogen peroxide (H_2O_2) solution and boiled at a constant temperature of 80 °C for 1 hour. This step is designed to remove organic impurities and potential contaminants from the membrane surface. Afterwards, the membrane is thoroughly rinsed twice with deionized water to ensure the removal of residual hydrogen peroxide. Next, the cleaned membrane is transferred to a 5% sulfuric acid (H_2SO_4) solution and boiled continuously at 80 °C for another hour. This acid wash stage further cleans the membrane surface and potentially modifies its surface chemistry, preparing it for subsequent ion exchange. Following this, the membrane is again rinsed twice with deionized water to thoroughly remove any sulfuric acid residue. Subsequently, the membrane is placed in a 1 M KOH solution and boiled under 80 °C conditions for 2 h. This crucial step achieves the transformation from a proton-conductive (H^+) to a potassium-ion-conductive (K^+) membrane, as potassium ions in the KOH solution exchange with hydrogen ions within the membrane, thereby imparting new conductive properties. Finally, the processed and cleaned membrane is soaked in a 1 M KCl solution to maintain the stability of its potassium-ion conductivity during storage and use.

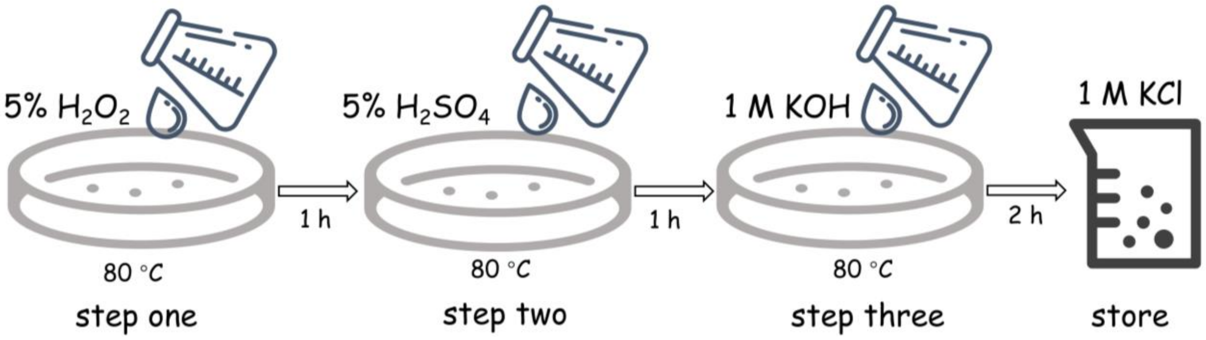

Figure S41. Schematic diagram of ion-exchange membrane processing steps

26. Symmetric battery

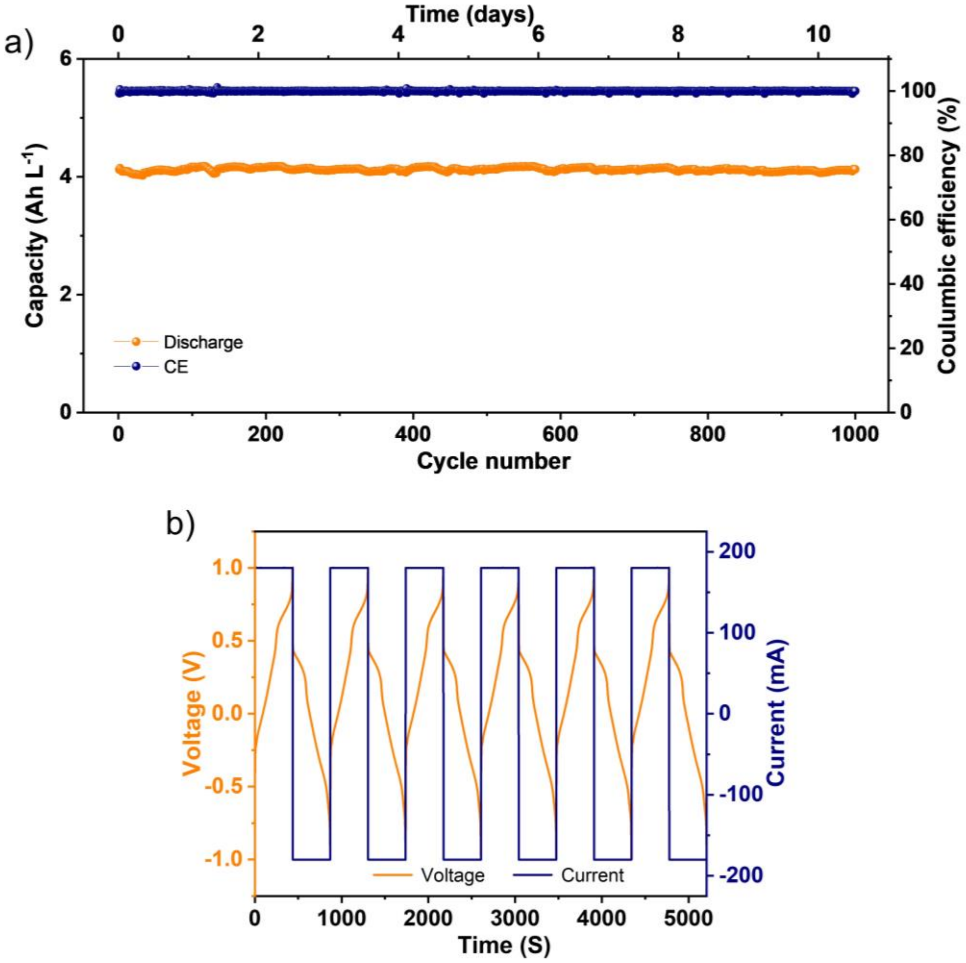

Figure S42. Symmetrical battery experiment of **(SPr)$_2$NDI**. a) Long-term cycling profile of the 0.1 M **(SPr)$_2$NDI/(SPr)$_2$NDI^{2-}** AORFB. b) Example of symmetric battery tests.

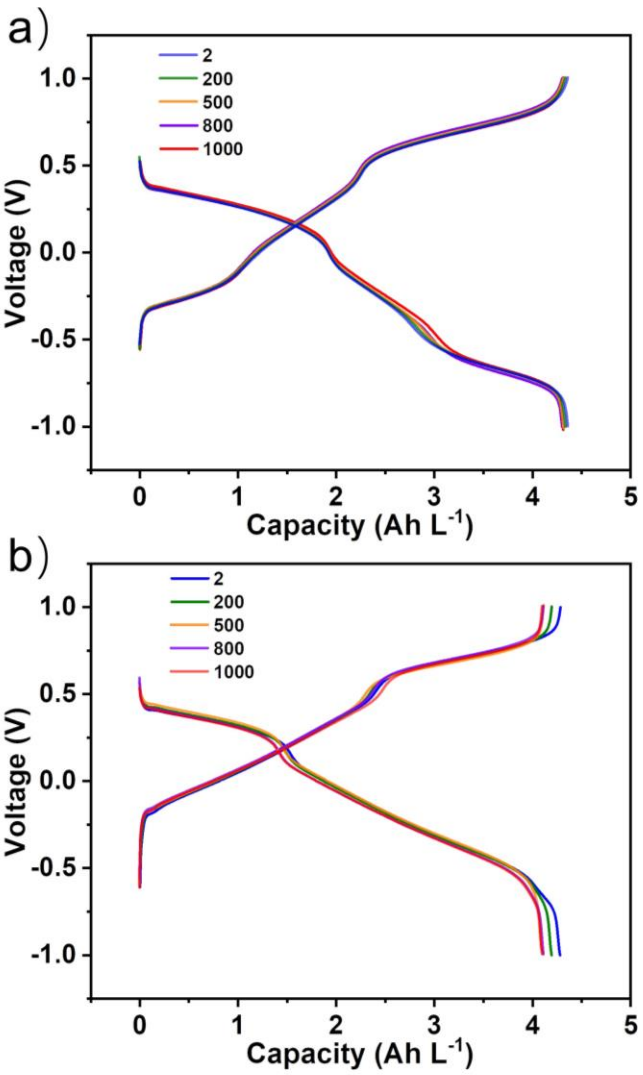

Figure S43. Representative charge and discharge curves of a) 0.1 M **$(CBu)_2NDI/(CBu)_2NDI^{2-}$** and b) 0.1 M **$(SPr)_2NDI/(SPr)_2NDI^{2-}$** in symmetric battery test for two-electron storage.

27. Full battery tests

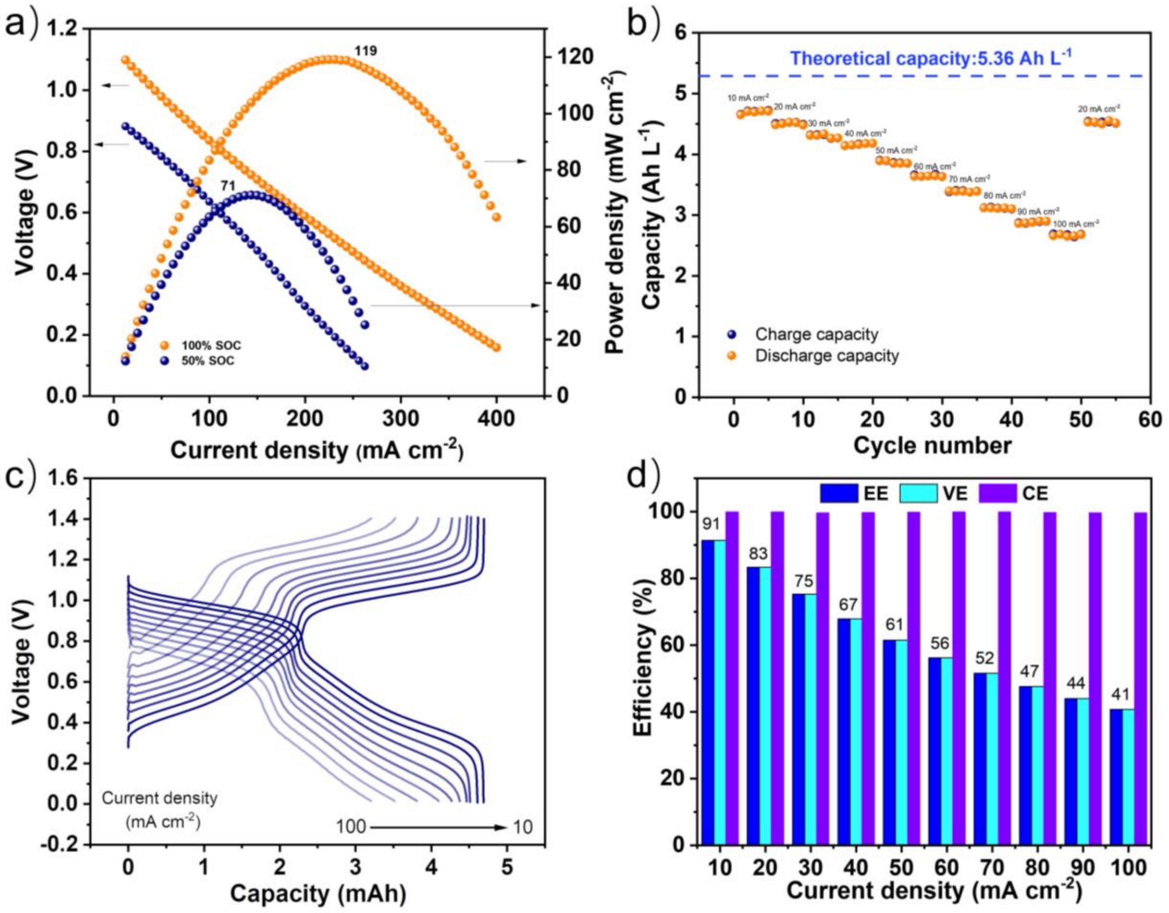

Figure S44. The rate performances at different current densities from 10 mA cm^{-2} to 100 mA cm^{-2} with a 10 mA cm^{-2} increment. a) Polarization and power density curves after full charge using 5 mA cm^{-2}. b) Plot of the battery capacity versus cycling numbers. c) Representative charge and discharge curves. d) Plot of average CE, EE, and VE. Condition: Anolyte: 0.1 M **(SPr)$_2$NDI** in 1 M KCl (5 mL); Catholyte: 0.1 M **$K_4Fe(CN)_6$** in 1 M KCl (12 mL). The 0.1 M **(SPr)$_2$NDI**/$K_4Fe(CN)_6$-based AORFB with a depth of 2 mm for graphite plate and a thickness of 2.5 mm for graphite felt. Nafion 212 cation-exchange membrane, 25 ^oC.

28. Conductivity, viscosity and ^{1}H NMR

Operational procedure of conductivity: The **(CBu)$_2$NDI** was individually prepared at four concentration gradients (0.1 M, 0.2 M, 0.5 M,1.0 M) in volumes of 10 mL at 25 °C. Here, **(CBu)$_2$NDI** for all concentrations were dissolved in 1.0 M KCl. Before conducting the test, thoroughly clean the conductivity electrode using deionized water, then place it into the prepared solutions of different concentrations, and record the readings from the instrument.

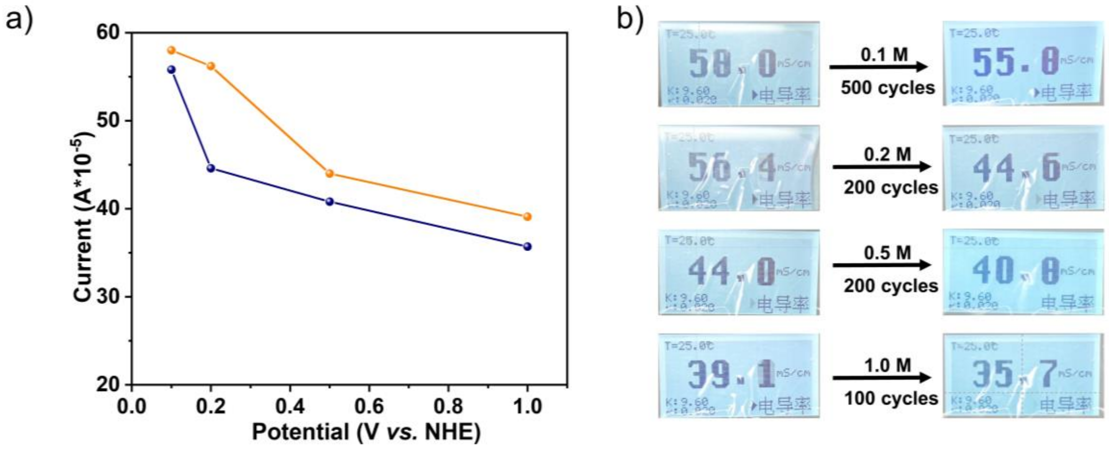

Figure S45. a) Conductivity of **(CBu)$_2$NDI** at different concentrations in 1 M KCl solution (before and after cycling), b) The readings of the conductivity meter before and after cycling.

Operational procedure of viscosity: The **(CBu)$_2$NDI** was individually prepared at four concentration gradients (0.1 M, 0.2 M, 0.5 M,1.0 M) in volumes of 10 mL at 25 °C. Here, **(CBu)$_2$NDI** for all concentrations were dissolved in 1.0 M KCl. During measurement, firstly, the solution was drawn into the measuring column of the viscometer. Once the solution filled the entire measuring column, the viscometer was uprighted and kept vertical (Figure S46). Start timing when the solution reaches the calibration line between the first and second storage bulbs, and stop timing when the solution falls below the calibration line beneath the second storage bulb, recording the time (t). The viscosity (η) can be calculated from the following formula: $\eta = K \times t$, where K is the capillary constant. Corresponding inner diameters of capillaries were chosen based on the solution concentration, with 1.0 M using a 0.8 mm inner diameter (K = 0.0349 mm^2/s^2) and 0.1 M, 0.2 M and 0.5 M using a 0.6 mm inner diameter (K = 0.00936 mm^2/s^2). Therefore, the viscosity values of **(CBu)$_2$NDI** are 1.71 (0.1 M), 2.34 (0.2 M), 4.22 (0.5 M), and 8.53 mm^2 s^{-1} (1.0 M) respectively.

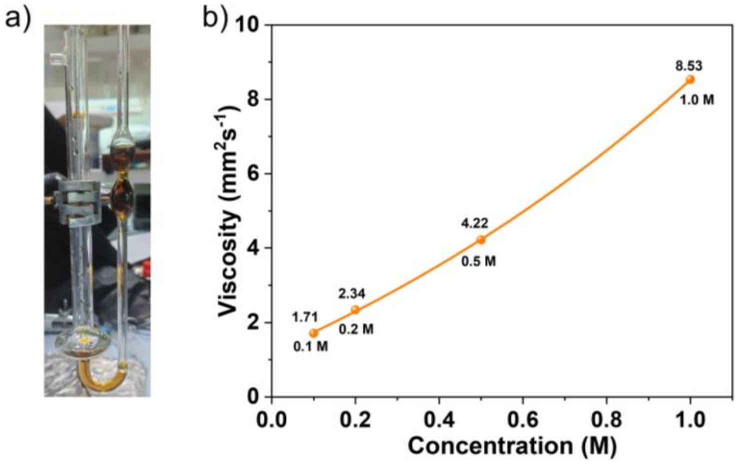

Figure S46. a) Schematic diagram of viscosity test. b) Viscosity of **(CBu)$_2$NDI** at different concentrations in 1 M KCl solution.

29. Electrochemical Impedance Spectroscopy (EIS)

The Potentio-controlled Electrochemical impedance spectroscopy (EIS) of batteries were obtained by Nava with a frequency range from 0.01 Hz to 10 kHz.

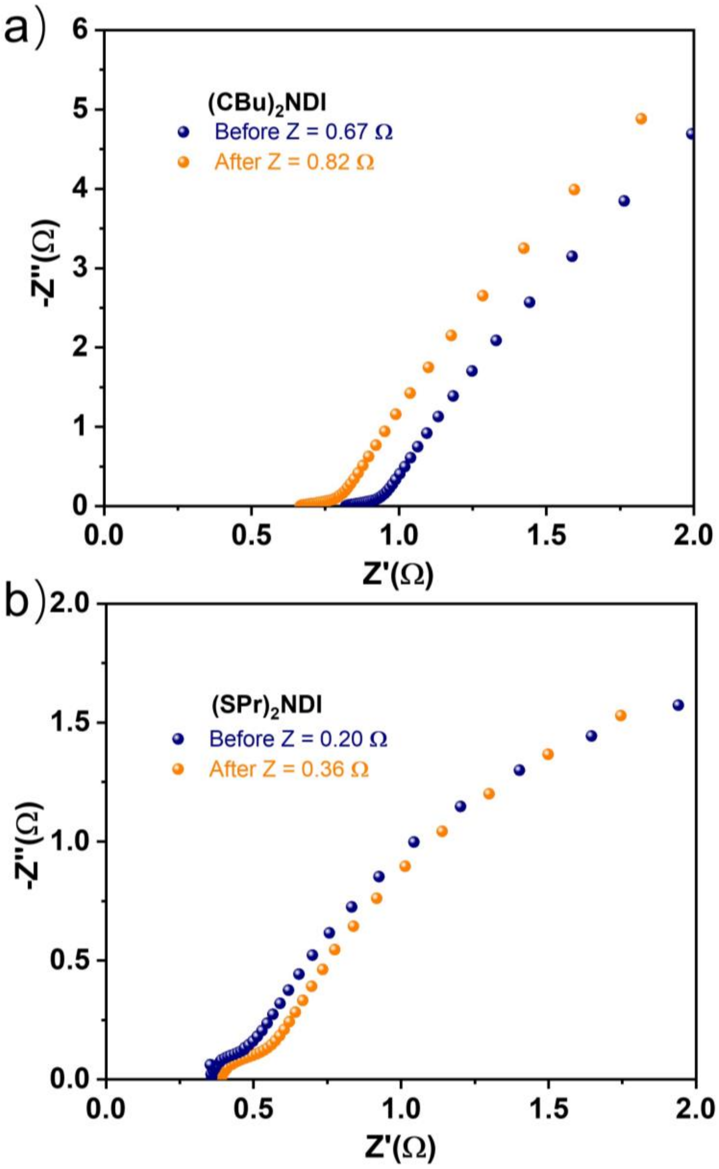

Figure S47. Electrochemical impedance spectroscopy (EIS) of a) 1 M **$(CBu)_2$NDI/$K_4Fe(CN)_6$**-based AORFB before and after 220 cycles and 0.25 M **$(SPr)_2$NDI/$K_4Fe(CN)_6$**-based AORFB before and after 1000 cycles

30. The cyclic voltammogram after the cycle of the battery test

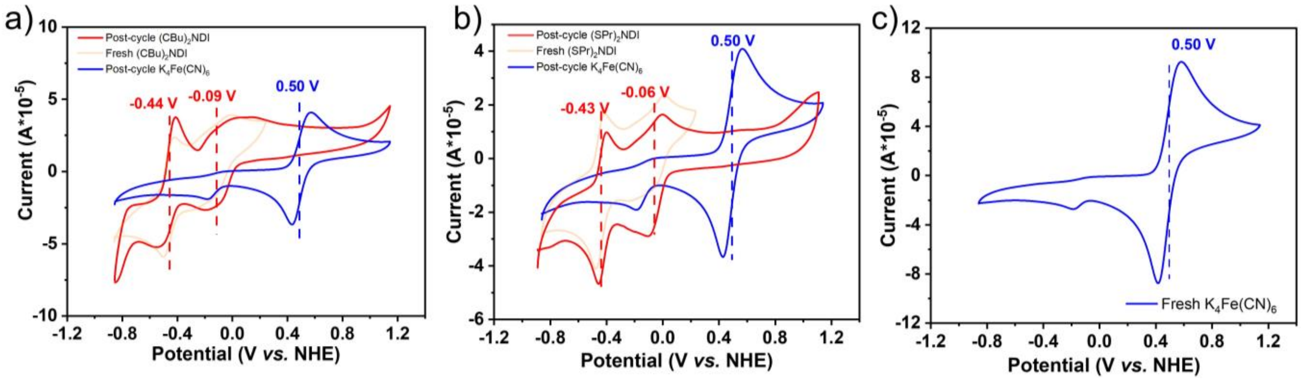

Figure S48. CV curves of a) 0.1 M **$(CBu)_2NDI/K_4Fe(CN)_6$**-based AORFB before and after 5070 cycles in a full battery (red: post-cycle **$(CBu)_2NDI$**, orange: fresh **$(CBu)_2NDI$**, blue: post-cycle **$K_4Fe(CN)_6$**.) b) 0.25 M **$(SPr)_2NDI/K_4Fe(CN)_6$**-based AORFB before and after 1000 cycles in a full battery (red: post-cycle **$(CBu)_2NDI$**, orange: fresh **$(CBu)_2NDI$**, blue: post-cycle **$K_4Fe(CN)_6$**.). c) 4 mM **$K_4Fe(CN)_6$** fresh electrolyte.

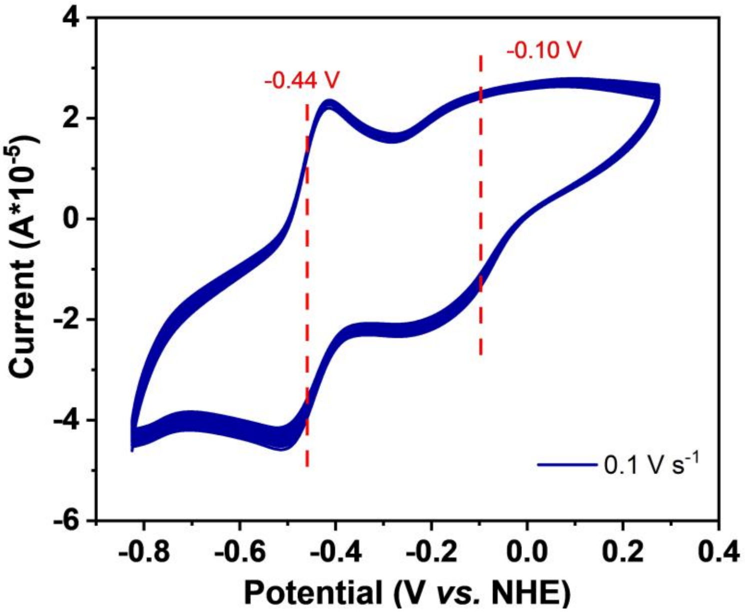

Figure S49. The cyclic voltammogram at different scan rates at 0.1 V S^{-1} of the recovered **$(CBu)_2NDI$**.

31. ^{1}H NMR and UV/Vis characterization of $(CBu)_2$NDI after cycling

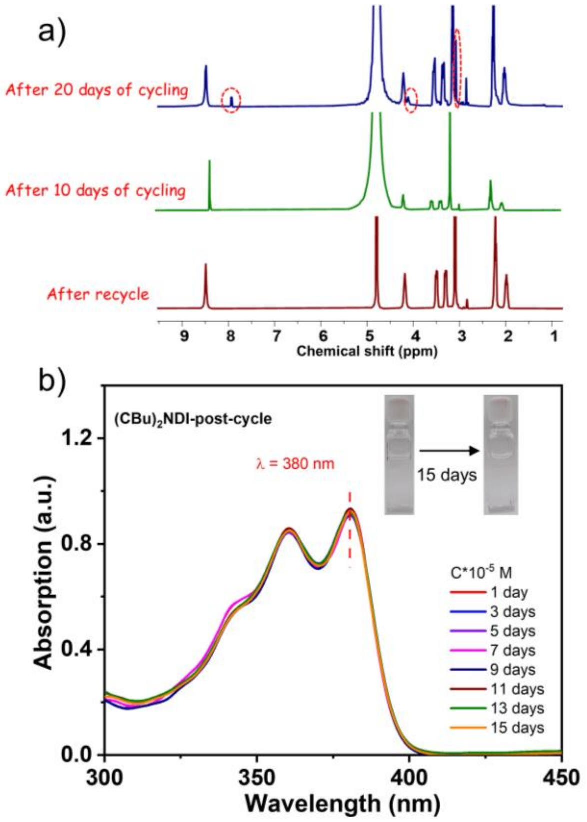

Figure S50. a)The ^{1}H NMR of **$(CBu)_2$NDI** after cycling (the battery was cycled for 100 cycles at 1 M **$(CBu)_2$NDI**) and recovery. b) The UV absorption change of **$(CBu)_2$NDI** during 15 days after cycling.

32. Simulation of molecule penetration through Nafion membrane.

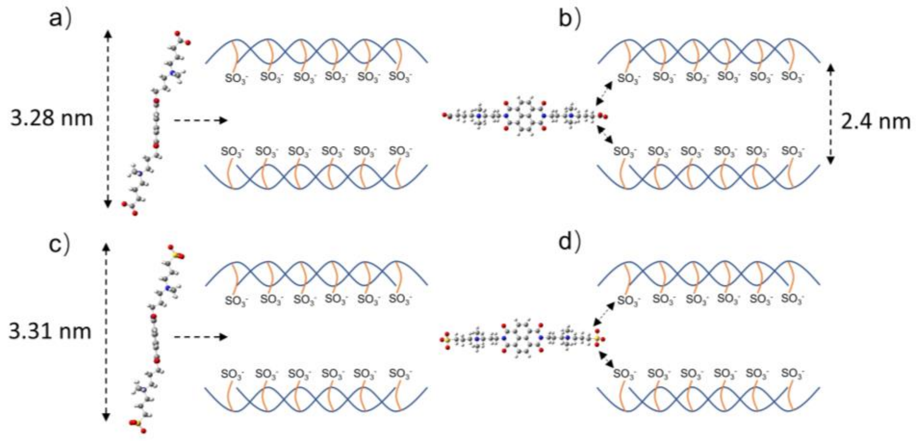

Figure S51. Proposed interaction of a) **$(CBu)_2$NDI** b) **$(SPr)_2$NDI** represented in a space-filling model adopting perpendicular orientation with the Nafion 212. Proposed interaction of c) **$(CBu)_2$NDI** d) **$(SPr)_2$NDI** represented in a space-filling model adopting parallel orientation with the Nafion 212.

33. Permeability measurement of $(CBu)_2$NDI.

Permeability measurements:

The membrane permeability of active materials was measured with a homemade H-tube, assembling with two L-shaped glass tubes, two glass stoppers, two metal clip, and two gaskets separated by a piece of Nafion 212 membrane. 0.2 M **$(CBu)_2$NDI** in 1 M NaCl was placed on one storage tank as the donating side, while the other storage tank filled with 30 mL 1 M NaCl was used as the receiving side. The osmotic pressure at both ends was adjusted to equilibrate, ensuring that no water molecules migrate. The whole device was placed on the agitator, and the magneton was added for stirring at all times. The crossover of active materials was periodically monitored by measuring the concentration of the solution in the receiving side by UV/Vis.

$$p = \frac{\text{In}(1-\frac{2\text{Ct}}{\text{C0}})(-\frac{\text{Vol}}{2\text{A}})}{\Delta t}$$

Where P is the permeability ($cm^2\ s^{-1}$), C_t is the concentration measured at the receiving side at time t, C_0 (0.2 M) is the initial concentration of active materials at the donating side. V_0 (30 mL) is the initial volume on either the receiving or donating sides. A (2π cm^2) is the effective membrane area. l (50.8 μm) is the thickness of the Nafion 212 membrane. t is the time (s).

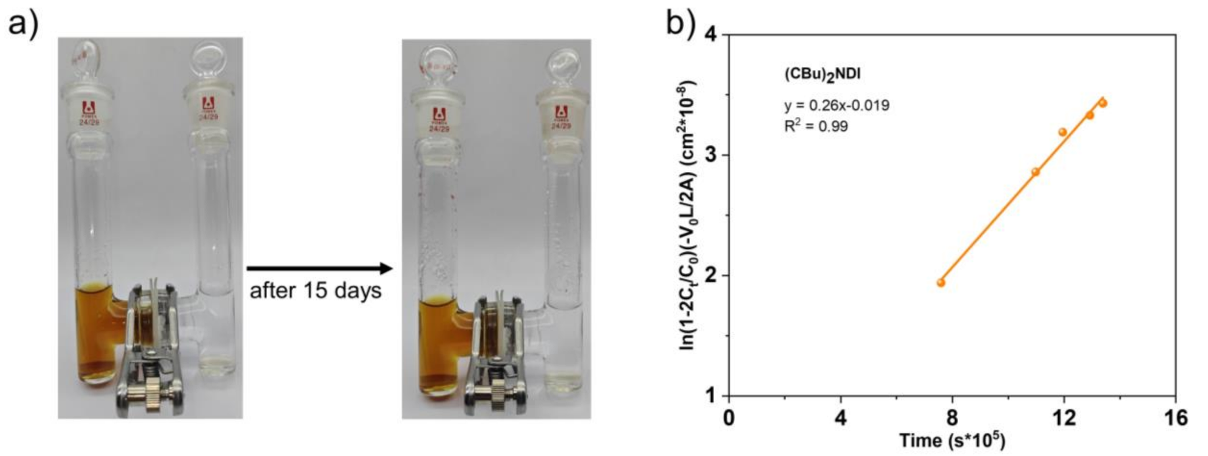

Figure S52. a) The color change of active materials for day 1 and day 15. b)The permeability of **$(CBu)_2$NDI** across a Nafion 212 membrane.

It can be seen that the color of the receiving side did not change over 15 days. Due to the low molecular crossover rate and the detection limit of the UV/Vis spectrophotometer, we can only estimate the permeability of **$(CBu)_2$NDI**. According to the Fick's Law, the permeability of **$(CBu)_2$NDI** should be lower than $2.6 \times 10^{-14}\ cm^2\ s^{-1}$.

34. Electrolyte recovery experiment for $(CBu)_2NDI$.

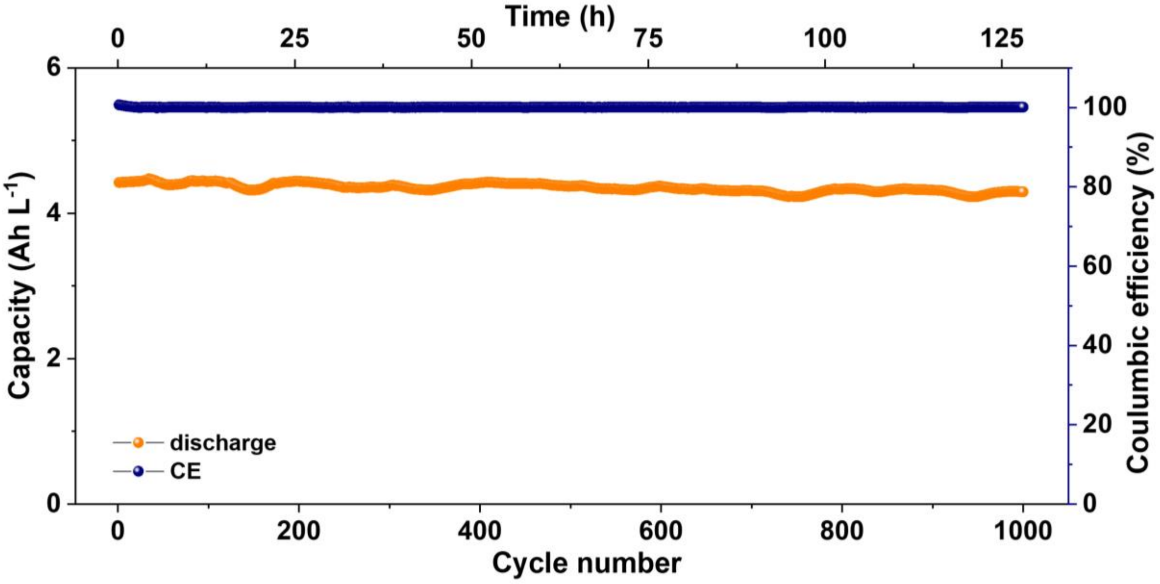

Figure S53. Extended 1000 cycles data of the recycled 0.1 M **$(CBu)_2NDI/K_4Fe(CN)_6$**-based AORFB showing discharge capacity and Coulombic efficiency versus cycle number and time at 40 mA cm^{-2}. Inset: ^1H NMR spectra of (CBu)2NDI after cycling and recycling.

35. ^{1}H, ^{13}C NMR spectra

^{1}H NMR (D_2O, 400 MHz) and ^{13}C NMR (D_2O, 101 MHz) spectra of **NDI-N.**

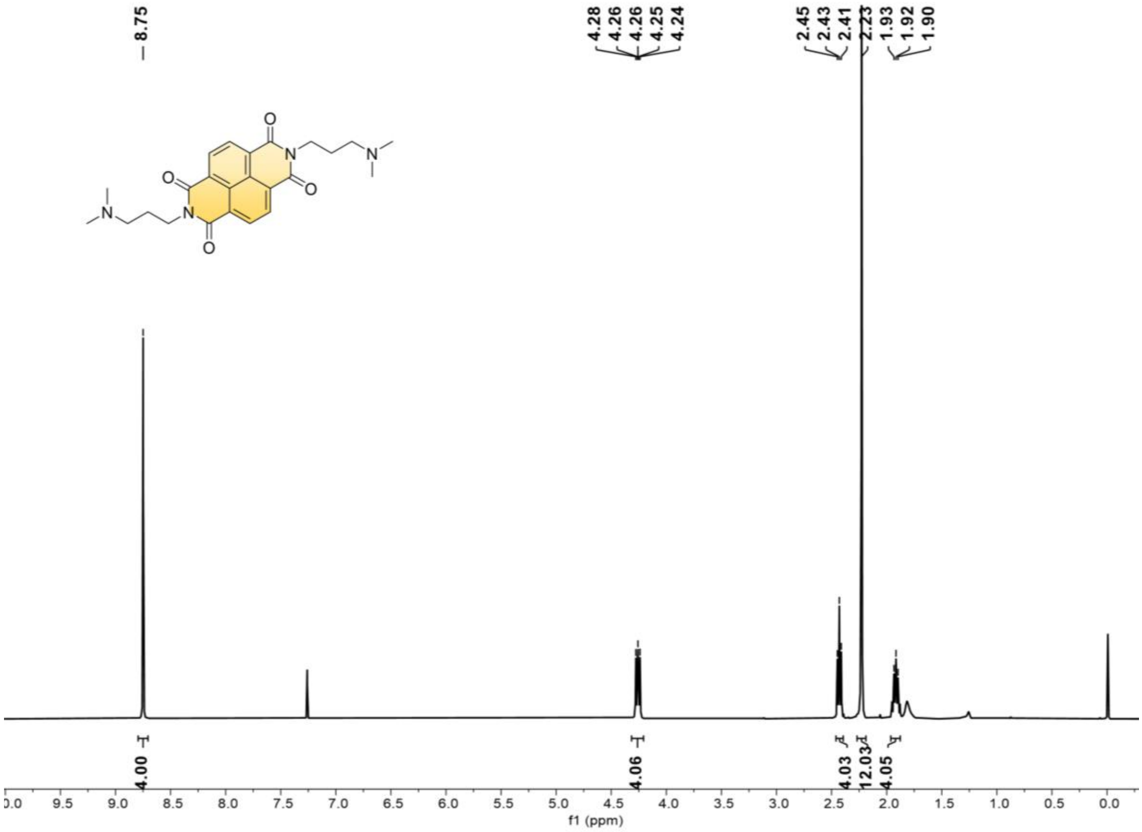

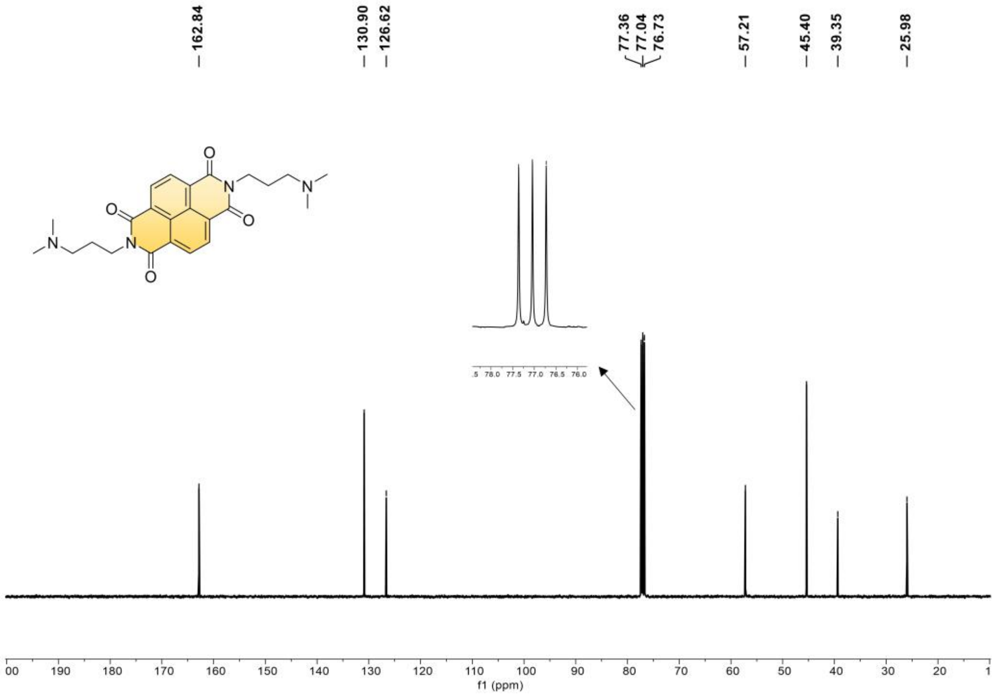

^{1}H NMR (D_2O, 400 MHz) and ^{13}C NMR (D_2O, 101 MHz) spectra of **[(EB)$_2$NDI]Br$_2$**:

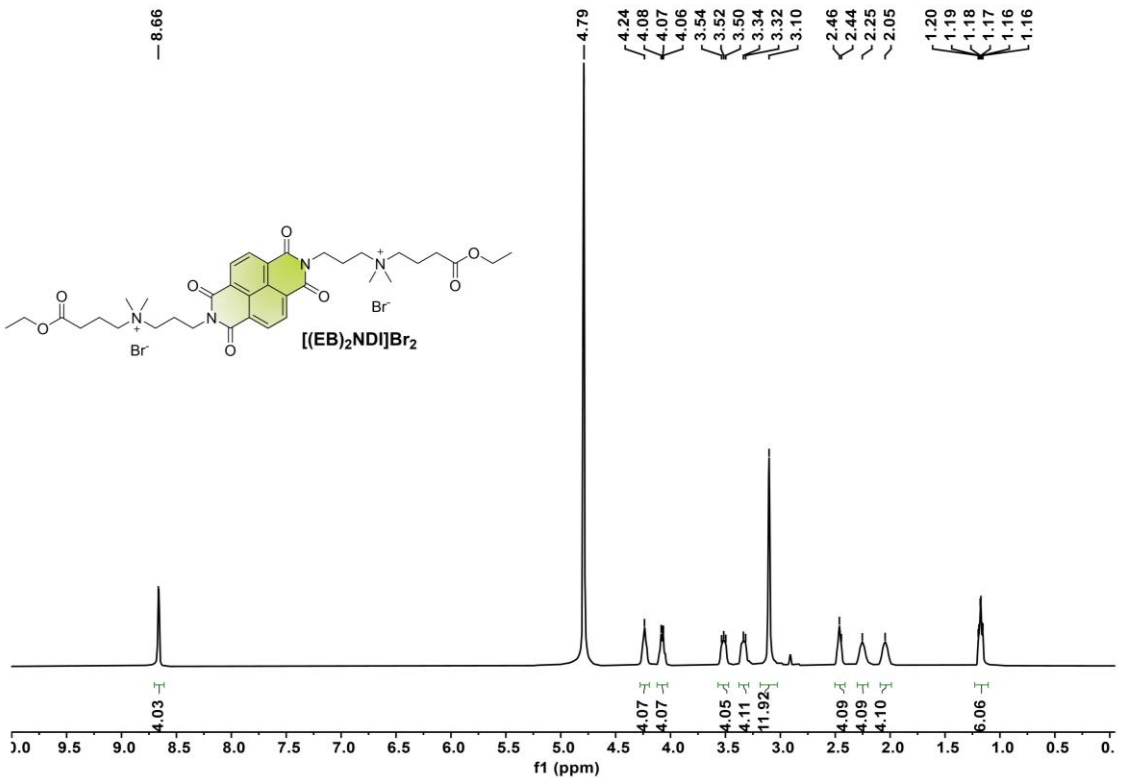

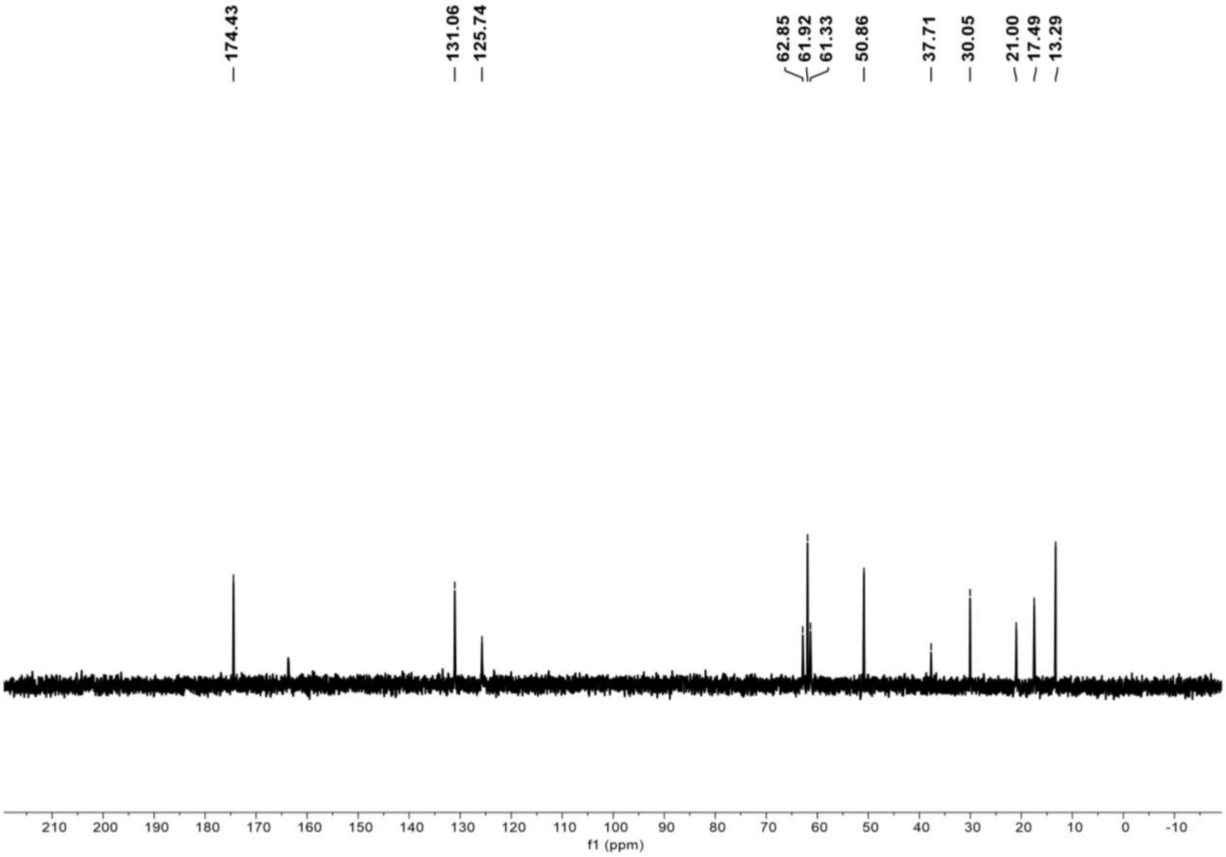

^{1}H NMR (D_2O, 400 MHz) and ^{13}C NMR (D_2O, 101 MHz) spectra of **$[(CBu)_2NDIH_2]Br_2$**:

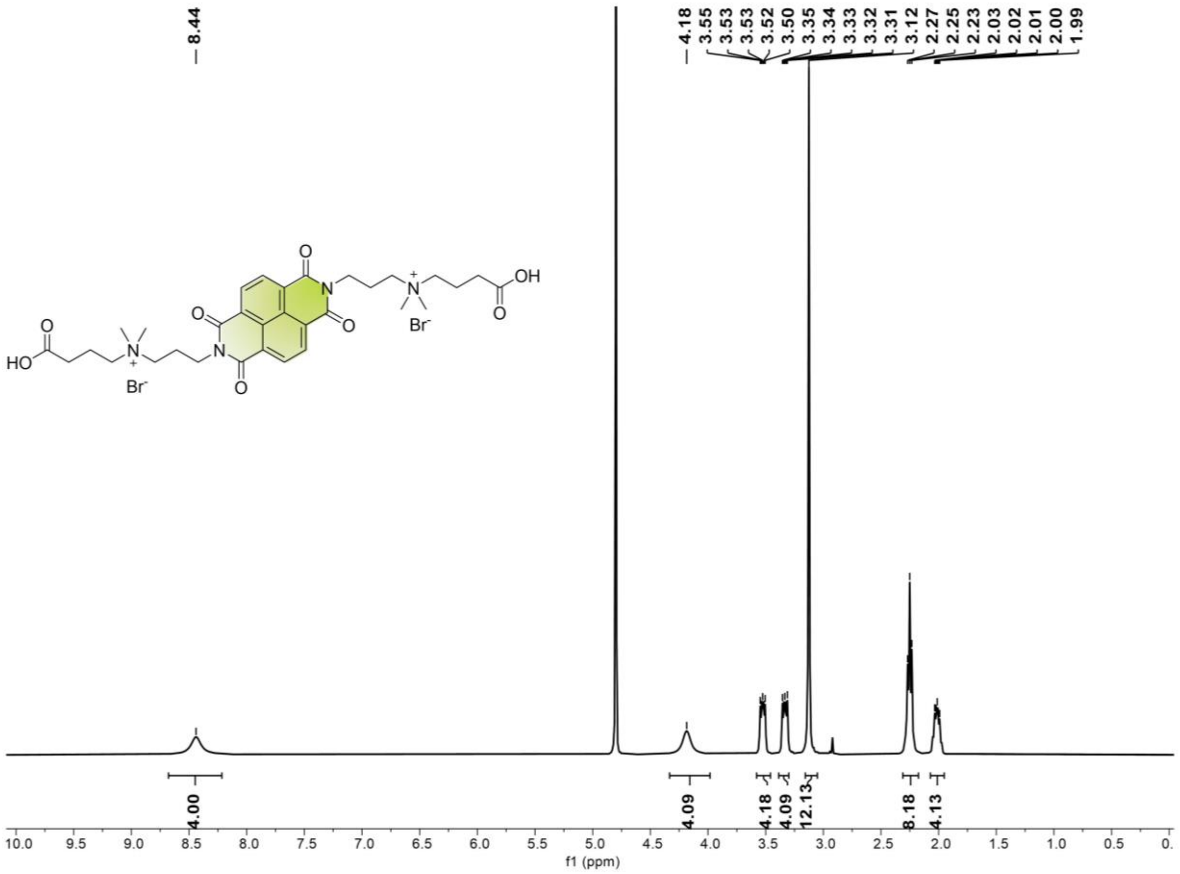

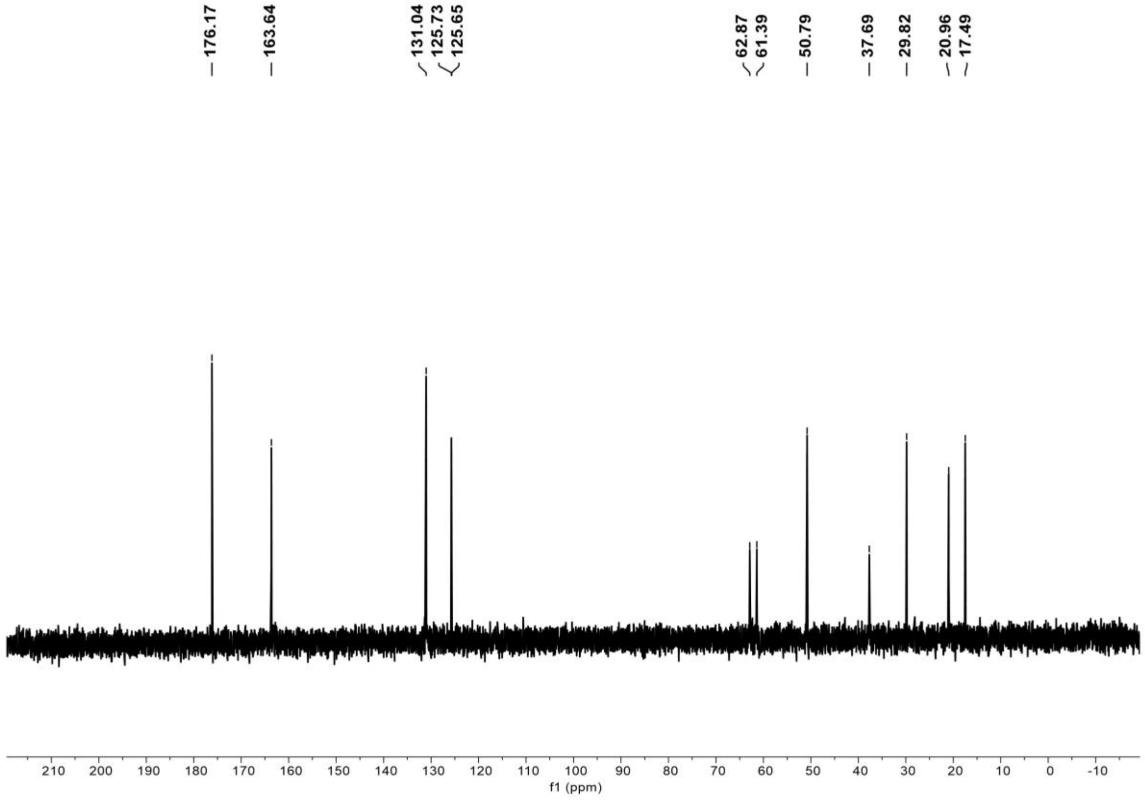

^{1}H NMR (D_2O, 400 MHz) and ^{13}C NMR (D_2O, 101 MHz) spectra of **$(CBu)_2$NDI**:

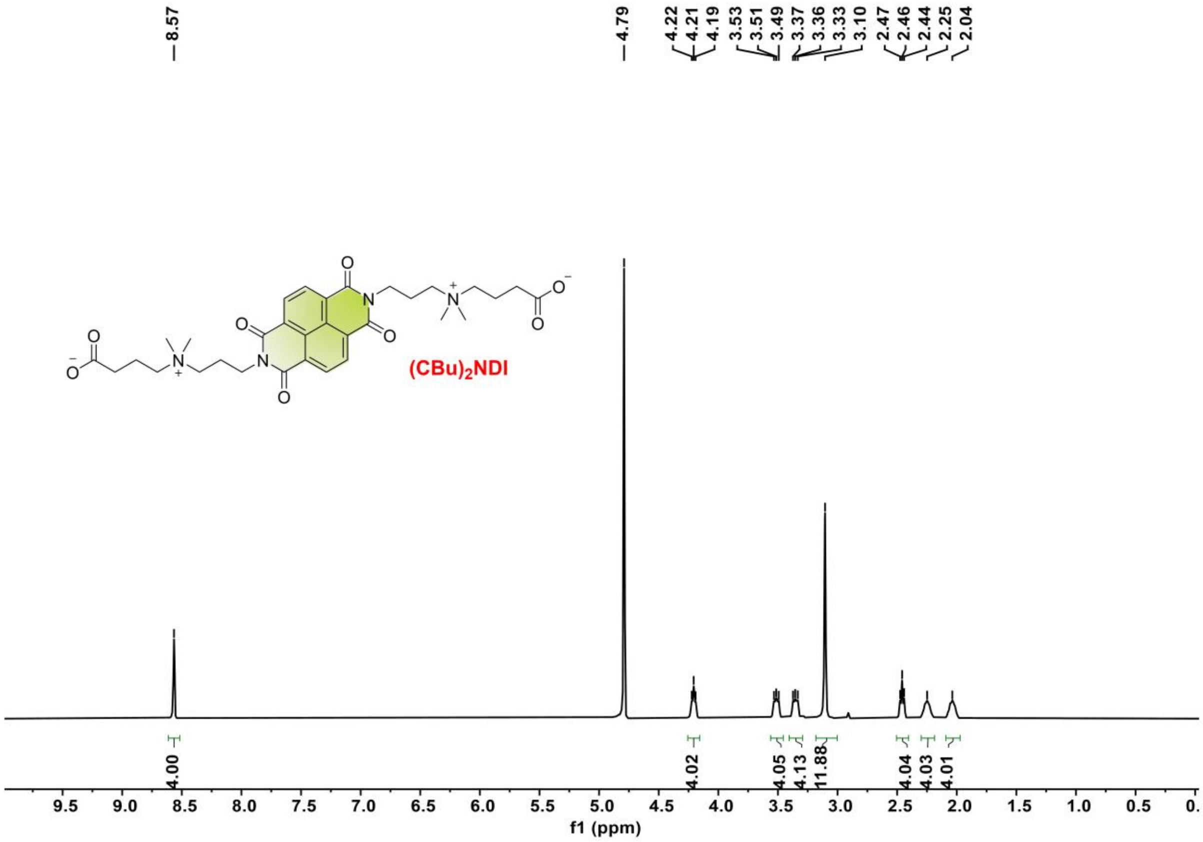

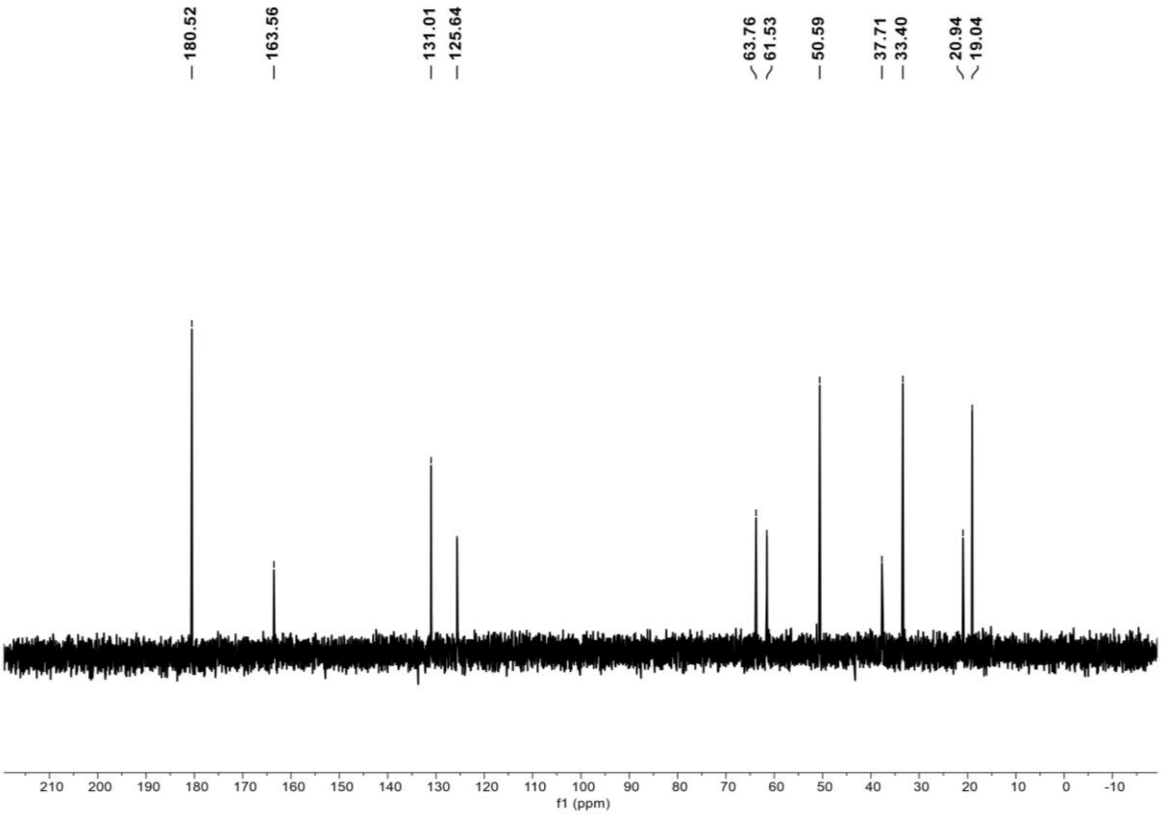

^{1}H NMR (D_2O, 400 MHz) and ^{13}C NMR (D_2O, 101 MHz) spectra of **$(SPr)_2NDI$**:

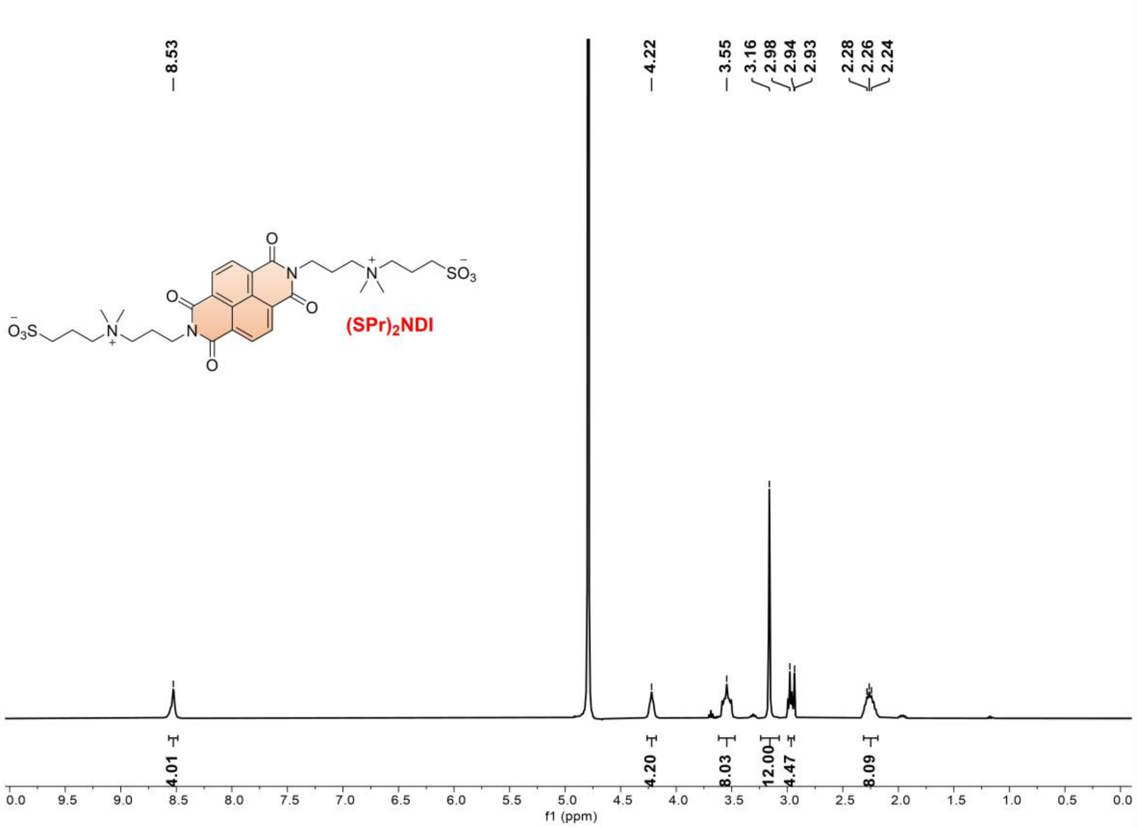

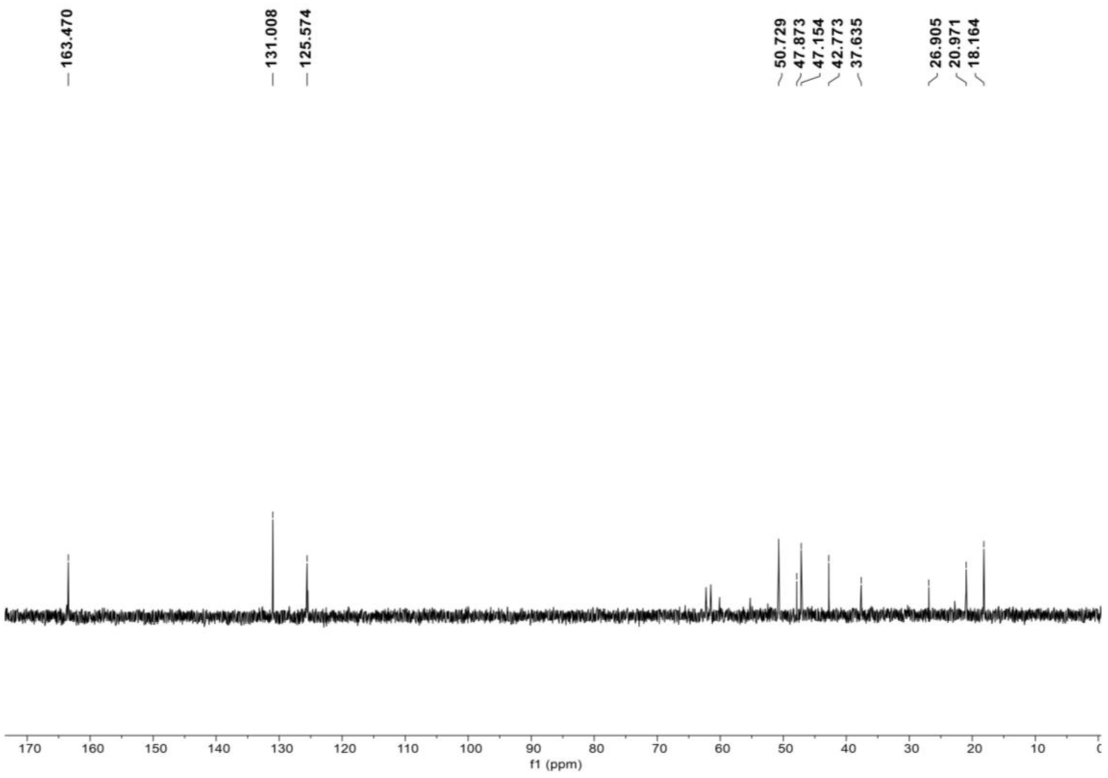

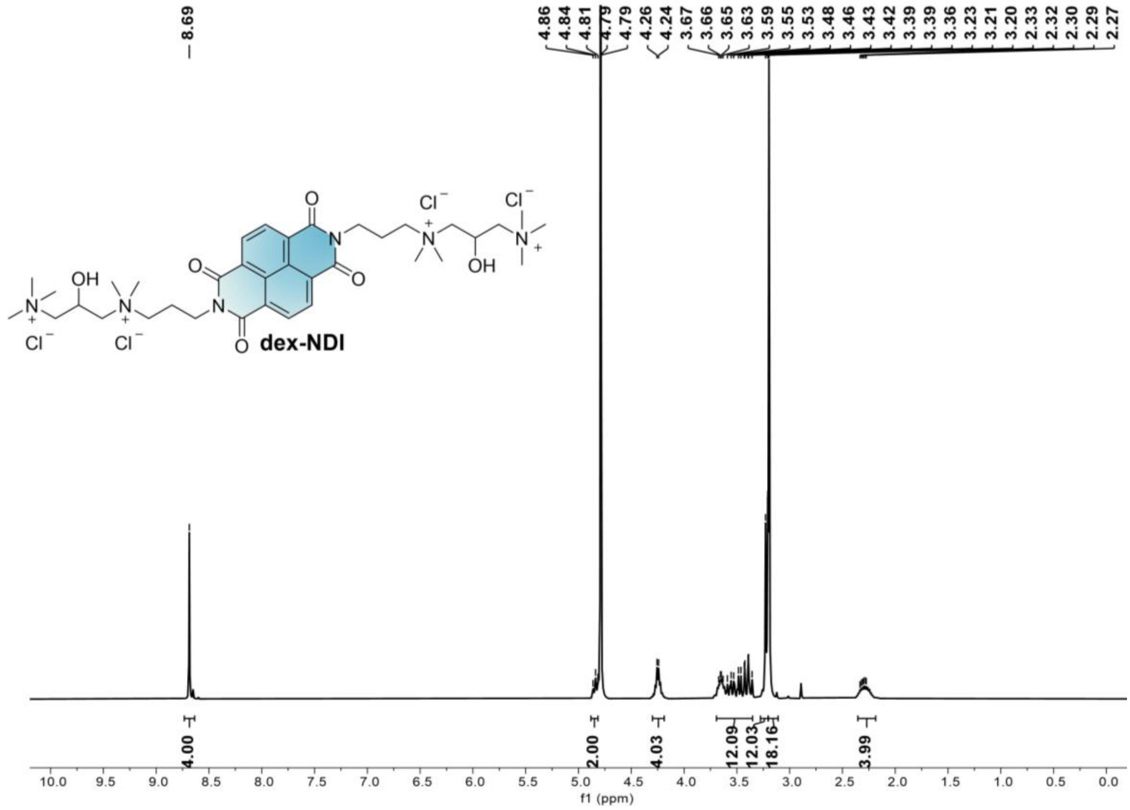

8.69
4.86
4.84
4.81
4.79
4.79
4.26
4.24
3.67
3.66
3.65
3.63
3.59
3.55
3.53
3.48
3.46
3.43
3.42
3.39
3.39
3.36
3.23
3.21
3.20
2.33
2.32
2.30
2.29
2.27
dex-NDI
OH
OH
Cl^-
4.00
2.00
4.03
12.09
12.03
18.16
3.99
f1 (ppm)

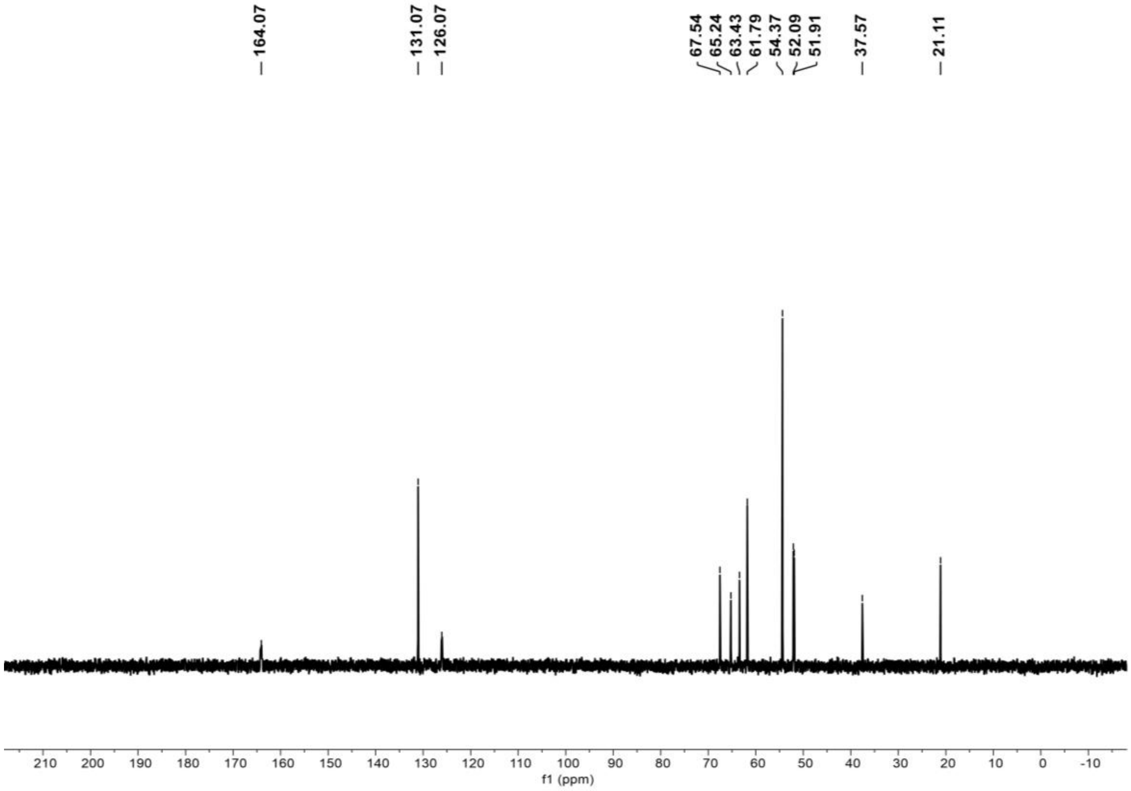

164.07
131.07
126.07
67.54
65.24
63.43
61.79
54.37
52.09
51.91
37.57
21.11
f1 (ppm)

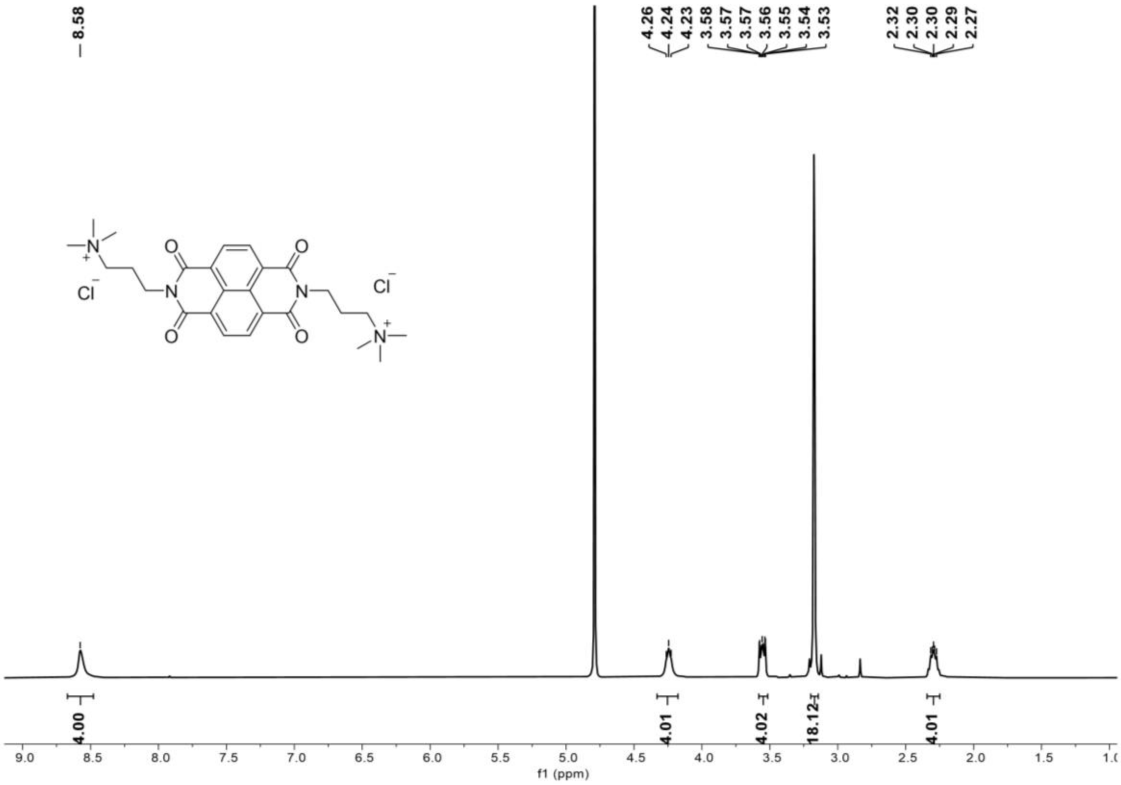
8.58
4.26
4.24
4.23
3.58
3.57
3.57
3.56
3.55
3.54
3.53
2.32
2.30
2.30
2.29
2.27
Cl⁻
Cl⁻
4.00
4.01
4.02
18.12
4.01
f1 (ppm)

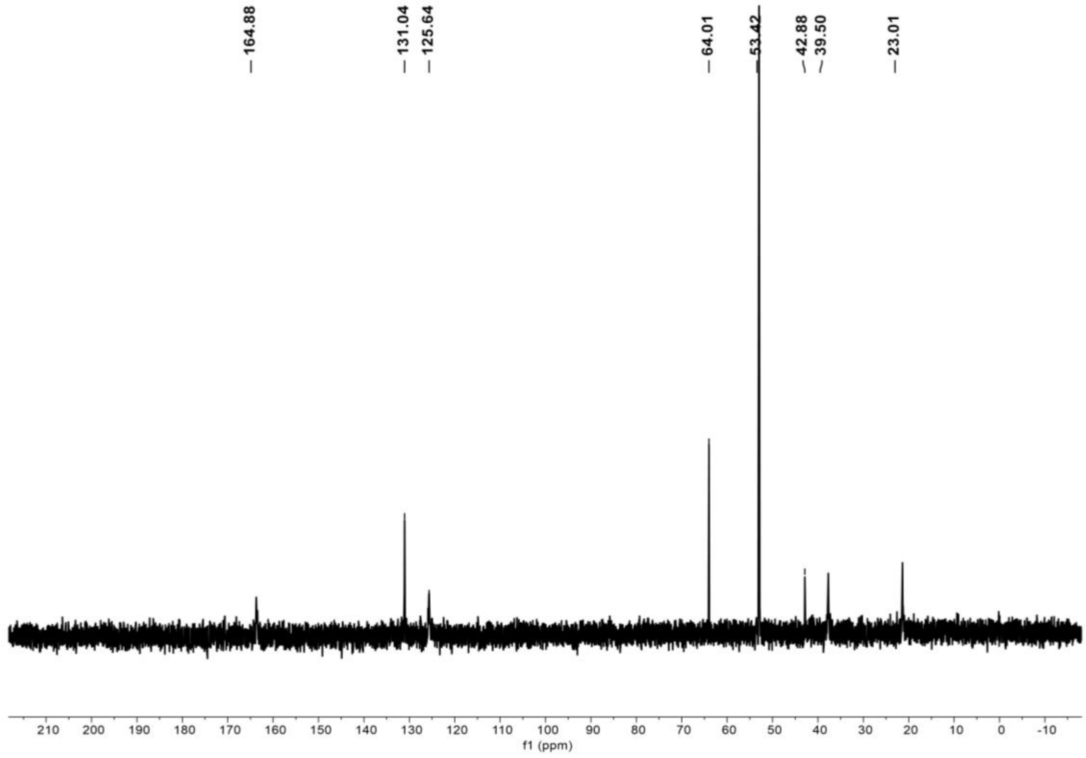
164.88
131.04
125.64
64.01
53.42
42.88
39.50
23.01
f1 (ppm)

36. Anolytes and catholytes price comparison

Table S4. The total cost for per Ah of the representative AORFBs.

AORFB	Cost ($ Ah^{-1})
$(CBu)_2NDI/K_4Fe(CN)_6$	6.58
$(SPr)_2NDI/K_4Fe(CN)_6$	4.27
dex-NDI/MiAcNH-TEMPO	37.63
$4A^{4+}$-NDI / KI	30.07
$[(NPr)_2TTz]Cl_4$ / NMe-TEMPO	119.85
1,6-DPAP / $K_4Fe(CN)_6$	37.84

Anolyte :

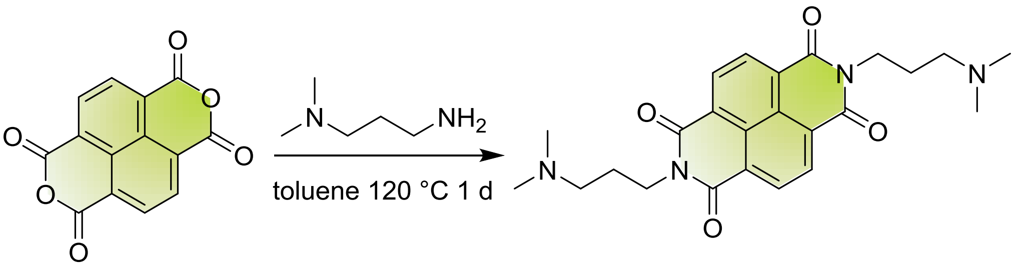

Cost analysis of NDI-N on a laboratory-scale. (This work)

Chemical	NTCDA	3-(dimethylamino)-1-propylamine	**NDI-N(95%)**
Ratio	1.0 eq	3.0 eq	/
MW	268.18 g mol^{-1}	102.18 g mol^{-1}	436.51 g mol^{-1}
Price	$0.32 g^{-1}	$23.7 L^{-1}	**$0.26 g^{-1}**
Toluene ($4.17), postprocessing ($1.39)			
Cost: $26/100 g (Including raw materials and solvent)			

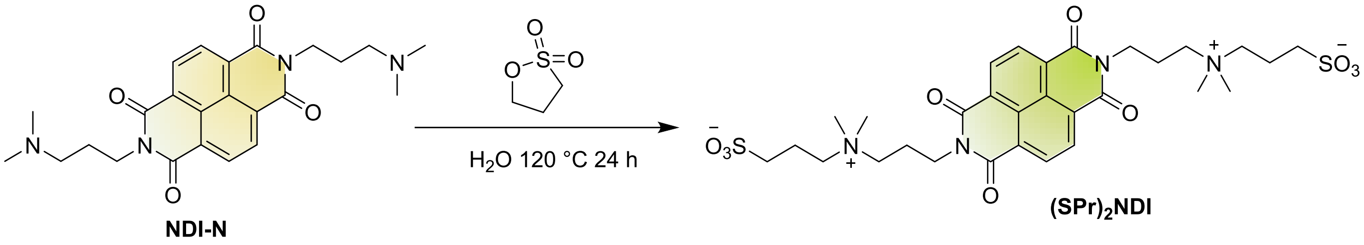

Cost analysis of **$(SPr)_2$NDI** on a laboratory-scale. (This work)

Chemical	NDI-N	1,3-Propanesultone	**$(SPr)_2$NDI(90%)**
Ratio	1.0 eq	3.0 eq	/
MW	436.51 g mol^{-1}	122.14 g mol^{-1}	680.79 g mol^{-1}
Price	$0.26 g^{-1}	$0.036 g^{-1}	**$0.229 g^{-1}**
postprocessing ($ 3)			
Cost: $22.9/100 g (Including raw materials and solvent)			

Cost analysis of **(CBu)$_2$NDI** on a laboratory-scale. (This work)

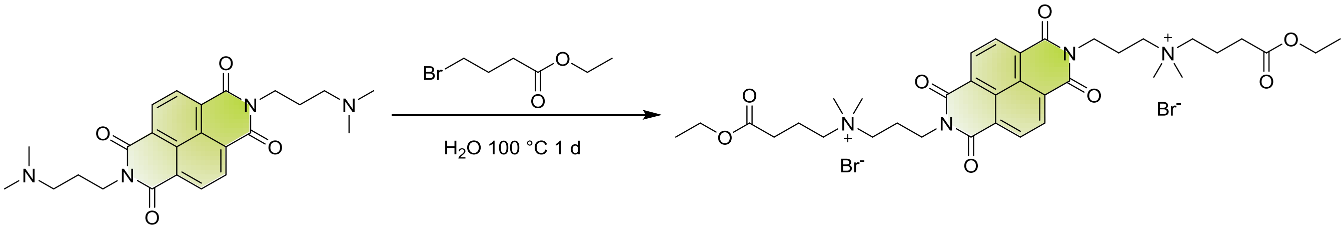

Chemical	NDI-N	Ethyl 4-bromobutyrate	**NDI-COOC$_2$H$_5$(90%)**
Ratio	1.0 eq	3.0 eq	/
MW	436.51 g mol^{-1}	195.06 g mol^{-1}	826.62 g mol^{-1}
Price	\$0.26 g^{-1}	\$0.046 g^{-1}	**\$0.213 g^{-1}**
postprocessing (\$ 4.17)			
Cost: \$21.3/100 g (Including raw materials and solvent)			

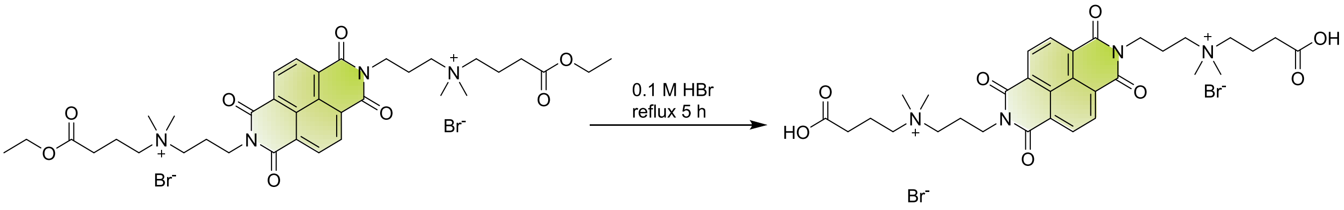

Chemical	NDI-COOC$_2$H$_5$	HBr (0.1 M)	**[(CBu)$_2$NDIH$_2$]Br$_2$(92%)**
Ratio	1.0 eq	22 eq	/
MW	826.62 g mol^{-1}	80.91 g mol^{-1}	770.52 g mol^{-1}
Price	\$0.213 g^{-1}	\$0.306 L^{-1}	**\$0.257 g^{-1}**
postprocessing (\$ 0)			
Cost: \$25.7/100 g (Including raw materials and solvent)			

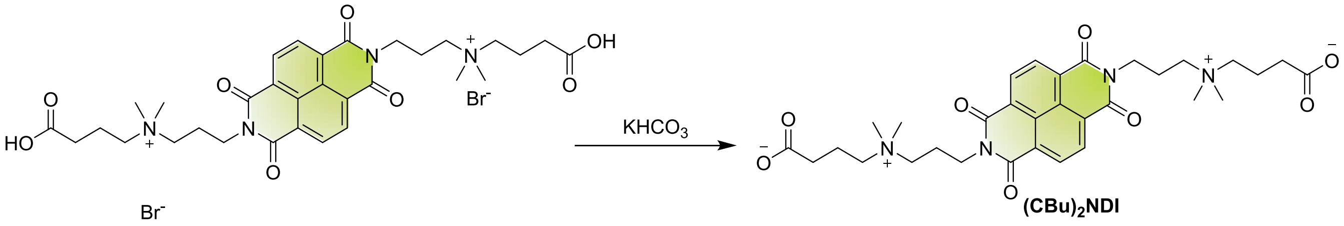

(CBu)$_2$NDI

Chemical	[(CBu)$_2$NDIH$_2$]Br$_2$	KHCO$_3$	**(CBu)$_2$NDI(82%)**
Ratio	1.0 eq	2.0 eq	/
MW	770.52 g mol^{-1}	100 g mol^{-1}	608.69 g mol^{-1}
Price	\$0.257 g^{-1}	\$0.0116 g^{-1}	**\$0.46 g^{-1}**
solvent (\$ 4.17)			
Cost: \$46/100 g (Including raw materials and solvent)			

Cost analysis of **(CBu)$_2$NDI** on a Industrial production.

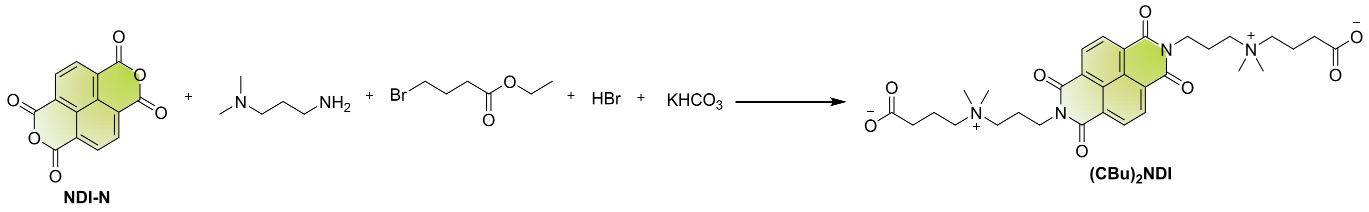

Chemical	NTCDA	3-(dimethylamino)-1-propylamine	Ethyl 4-bromobutyrate	HBr(0.1 M)	$KHCO_3$	**$(CBu)_2$NDI**
Ratio	1.0 eq	3.0 eq	2.85 eq		1.8 eq	/
MW	268.18 g mol^{-1}	102.18 g mol^{-1}	195.06 g mol^{-1}	80.91 g mol^{-1}	100 g mol^{-1}	608.69 g mol^{-1}
Price	\$48.61 kg^{-1}	\$2.19 L^{-1}	\$6.25 kg^{-1}	\$0.0579 Kg^{-1}	\$3.6 kg^{-1}	**\$0.376 g^{-1}**
Solvent cost to produce 1 kg of product: toluene (\$1), postprocessing (\$4.9)						
Cost: \$37.55/kg						

Catholyte：
Cost analysis of **$K_4Fe(CN)_6$** on a laboratory-scale.

Chemical	$K_4Fe(CN)_6$
MW	368.34 g mol^{-1}
Price	\$ 0.079 g^{-1}

Cost analysis of **$K_4Fe(CN)_6$** on a Industrial production.

Chemical	$K_4Fe(CN)_6$
MW	368.34 g mol^{-1}
Price	\$ 3.755 Kg^{-1}

For the anolytes with double electron storage, 1 mol of material can store 53.6 Ah of electricity. So to store 1 Ah electricity in the laboratory, the price of **$(CBu)_2NDI/K_4Fe(CN)_6$** required is:

Chemical	**$(CBu)_2NDI$**	**$K_4Fe(CN)_6$**
Ratio	1 eq	2.5 eq
MW	608.69 g mol^{-1}	368.34 g mol^{-1}
m	11.35 g	17.18 g
Price	**$5.22**	**$1.36**
Total price	$6.58	

So to store 1 Ah electricity in the laboratory, the price of **$(SPr)_2NDI/K_4Fe(CN)_6$** required is:

Chemical	**$(SPr)_2NDI$**	**$K_4Fe(CN)_6$**
Ratio	1 eq	2.5 eq
MW	680.79 g mol^{-1}	368.34 g mol^{-1}
m	12.7 g	17.18 g
Price	$2.91	$1.36
Total price	$4.27	

For other materials for double electron storage anolyte：

dex-NDI anolyte：

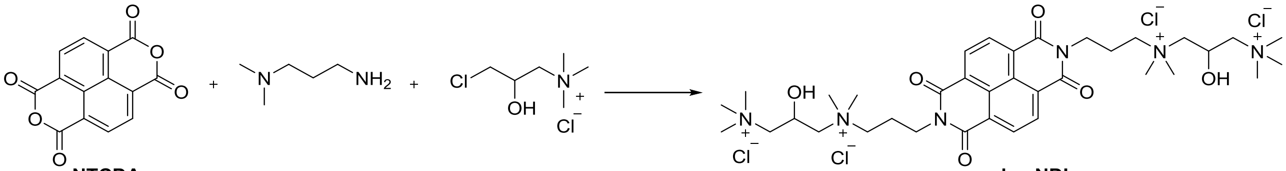

Chemical	NTCDA	3-(dimethylamino)-1-propylamine	(3-Chloro-2-hydroxypropyl) trimethylammonium chloride	**dex-NDI**
Ratio	1.0 eq	3.0 eq	2.85 eq	/
MW	268.18 g mol^{-1}	102.18 g mol^{-1}	188.09 g mol^{-1}	812.7 g mol^{-1}
Price	\$0.32 g^{-1}	\$23.7 L^{-1}	\$0.026 g^{-1}	**\$0.32 g^{-1}**
Solvent cost to produce 100 g of product: toluene (\$4.17), postprocessing (\$4.17)				
Cost: \$32/100 g, \$4.85/Ah				

MiAcNH-TEMPO catholyte:
Cost analysis of **MiAcNH-TEMPO** on a laboratory-scale.

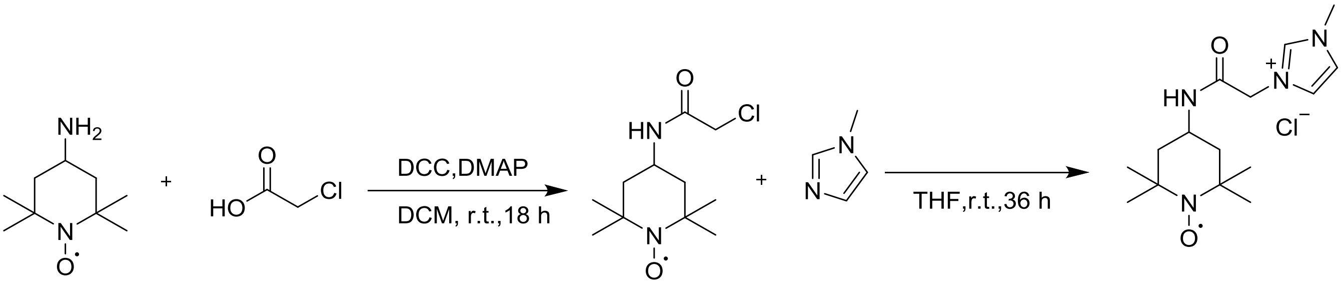

Chemical	NH_2-TEMPO	Chloroacetic acid	DCC	DMAP	1-Methylimidazole	**MiAcNH-TEMPO**
Ratio	1	1	0.25	1	1.45	/
MW	171.26 g mol^{-1}	94.49 g mol^{-1}	206.33 g mol^{-1}	122.17 g mol^{-1}	82.11 g mol^{-1}	329.85 g mol^{-1}
Price	\$1.83 g^{-1}	\$ 0.0139	\$ 0.124	\$ 0.043	\$ 0.050	**\$2.13 g^{-1}**
Solvent cost to produce 100 g of product: DCM and THF (\$ 27.8), postprocessing (\$ 6.95)						
Cost: \$213/100 g						

To store 1 Ah electricity in the laboratory, the price of **dex-NDI/MiAcNH-TEMPO** required is:

Chemical	**dex-NDI**	**MiAcNH-TEMPO**
Ratio	1 eq	2.5 eq
MW	812.7 g mol-1	329.85 g mol^{-1}
m	15.16 g	15.39 g
Price	\$4.85	\$32.78
Total price	\$37.63	

4A^{4+}-NDI anolyte :

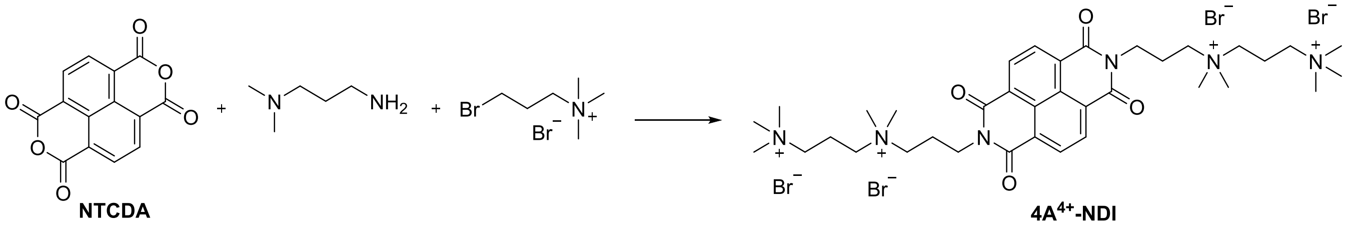

Chemical	NTCDA	3-(dimethylamino)-1-propylamine	(3-Bromopropyl) trimethylammonium bromide	**4A^{4+}-NDI**
Ratio	1.0 eq	5.0 eq	4.6 eq	/
MW	268.18 g mol^{-1}	102.18 g mol^{-1}	261 g mol^{-1}	958.51g mol^{-1}
Price	\$0.32 g^{-1}	\$23.7 L^{-1}	\$0.15 g^{-1}	**\$1.57 g^{-1}**
Solvent cost to produce 100 g of product: DMF (\$18), CH_3CN (\$80), postprocessing (\$75)				
Cost: \$157/100 g (Including raw materials and solvent)				

KI catholyte:
Cost analysis of **KI** on a laboratory-scale.

Chemical	KI
MW	166.00 g mol^{-1}
Price	\$ 0.208 g^{-1}

To store 1 Ah electricity in the laboratory, the price of **4A^{4+}-NDI** / **KI** required is:

Chemical	**4A^{4+}-NDI**	**KI**
Ratio	1 eq	3.1 eq
MW	958.51 g mol^{-1}	166.00 g mol^{-1}
m	17.88 g	9.6 g
Price	\$28.07	\$2
Total price	\$30.07	

[(NPr)$_2$TTz]Cl$_4$ anolyte :

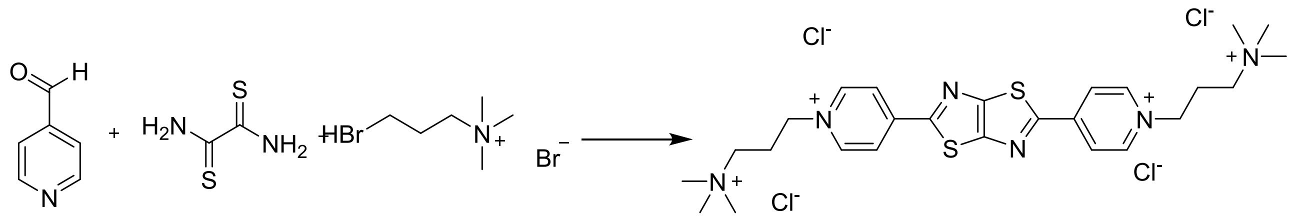

Chemical	4-Pyridinecarboxaldehyde	Dithiooxamide	(3-Bromopropyl)trimethylammonium bromide	**[(NPr)$_2$TTz]Cl$_4$**
Ratio	1.0 eq	0.5 eq	0.85 eq	/
MW	107.11 g mol^{-1}	120.19 g mol^{-1}	261.00 g mol^{-1}	640.55 g mol^{-1}
Price	\$0.334 g^{-1}	\$11.68 g^{-1}	\$0.15 g^{-1}	**\$5.326 g^{-1}**
Solvent cost to produce 100 g of product: DMF (\$38.94), postprocessing and Ion exchange column (\$ 20.8)				
Cost: \$533/100 g				

N^{Me}-TEMPO catholyte:
Cost analysis of **N^{Me}-TEMPO** on a laboratory-scale.

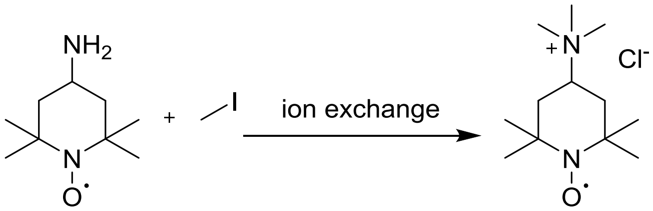

Chemical	NH$_2$-TEMPO	Iodomethane	N^{Me}-TEMPO
Ratio	1.0 eq	6.0 eq	
MW	171.26 g mol^{-1}	141.94 g mol^{-1}	249.8 g mol^{-1}
Price	\$ 1.83 g^{-1}	\$ 0.222 g^{-1}	**\$6.03 g^{-1}**
Solvent cost to produce 100 g of product: CH_3CN (\$ 5.56), postprocessing and Ion exchange column (\$ 22.25)			
Cost: \$ 603/100 g,			

To store 1 Ah electricity in the laboratory, the price of **[(NPr)$_2$TTz]Cl$_4$** / **N^{Me}-TEMPO** required is:

Chemical	**[(NPr)$_2$TTz]Cl$_4$**	**N^{Me}-TEMPO**
Ratio	1 eq	2.5 eq
MW	640.55 g mol^{-1}	249.8 g mol^{-1}
m	11.95 g	9.32 g
Price	\$63.65	\$56.20
Total price	\$119.85	

1,6-DPAP anolyte：

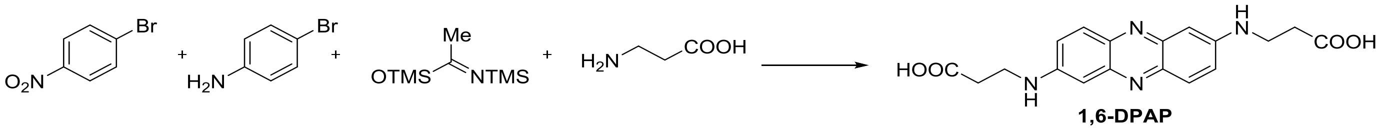

Chemical	1-Bromo-4-nitrobenzene	4-Bromoaniline	N,O-bis(trimethylsilyl)a-cetamide	β-Alanine	**1,6-DPAP**
Ratio	1.0 eq	1.0 eq	0.85 eq	0.36 eq	/
MW	202.01 g mol^{-1}	172 g mol^{-1}	203.43 g mol^{-1}	89.09 g mol^{-1}	354.37 g mol^{-1}
Price	\$0.53 g^{-1}	\$0.12 g^{-1}	\$35.9 L^{-1}	\$0.018 g^{-1}	**\$8.64 g^{-1}**
Solvent cost to produce 100 g of product: THF (\$70), CH_3CN (\$32), postprocessing (\$300). Other reactants and catalysts: KO^tBu (\$22), Brettphos (\$68) Tris(dibenzylideneacetone)dipalladium(0) (\$122)					
Cost: \$864/100 g (Including raw materials and solvent)					

$K_4Fe(CN)_6$ catholyte：

Chemical	$K_4Fe(CN)_6$
MW	368.34 g mol^{-1}
Price	\$ 0.079 g^{-1}

To store 1 Ah electricity in the laboratory, the price of **1,6-DPAP** / **$K_4Fe(CN)_6$** required is:

Chemical	**1,6-DPAP**	**$K_4Fe(CN)_6$**
Ratio	1 eq	7 eq
MW	354.37 g mol^{-1}	368.34 g mol^{-1}
m	6.61 g	48.10 g
Price	\$57.11	\$3.8
Total price	\$60.91	

37. Comparison of two-electron storage materials for neutral AORFBs.

Table S5. Comparison of two-electron storage materials for neutral AORFBs.

Catholyte	Anolyte	Con. active species (Electron concentration) (M)	Current density (mA cm^{-2})	Number of cycles	Battery voltage	capacity retention	Capacity fade (% per cycle)	Membrane	Ref.
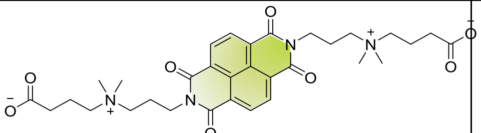 (CBu)$_2$NDI	$K_4Fe(CN)_6$	0.10 (0.20)	40	5070	0.92	100	0%	Nafion 212	This work
		1.00 (2.00)	20	220	0.92	100	0%	Nafion 212	This work
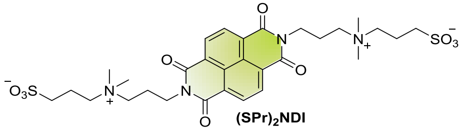 (SPr)$_2$NDI	$K_4Fe(CN)_6$	0.25 (0.50)	20	1000	0.91	100	0%	Nafion 212	This work
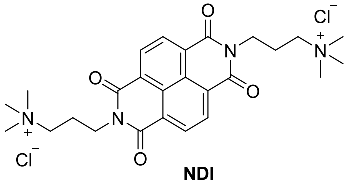 NDI	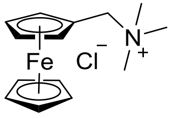 FcNCl	0.1 (0.2)	40	350	1.07	98.44	0.045	AMVN	[2]
		0.5 (1.00)	40	100	1.07	82.4	0.176	AMVN	[2]
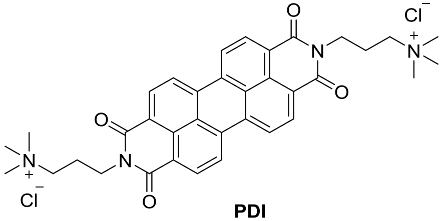 PDI		0.02 (0.04)	20	1500	1.06	98.1	0.0013	AMVN	[2]
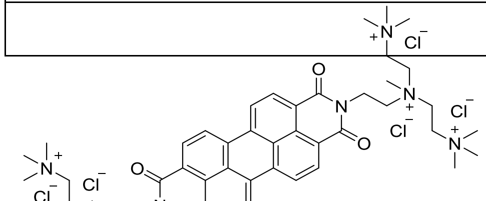		0.1 (0.2)	20	200	0.96	97.22	0.0139	AMVN	[2]

		0.05 (0.1)	20	2300	0.96	80	0.0087	AMVN	[2]
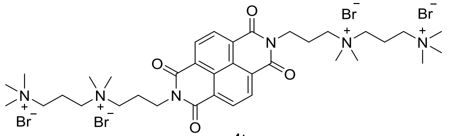 $4A^{4+}$-NDI	NH4I	1 (2)	20	500	1.15	98	0.004	Nafion	[3]
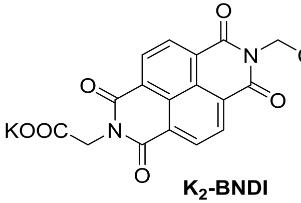 K_2-BNDI	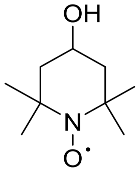 4-OH-TEMPO	0.025 (0.05)	5	100	1.27	83.2	0.168	AEM	[4]
		0.04 (0.08)	10	200	1.27	/	/	AEM	[4]
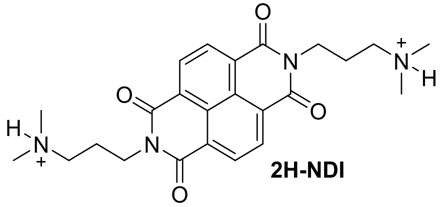 2H-NDI	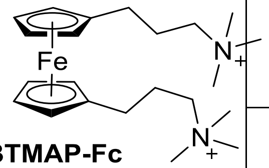 BTMAP-Fc	0.05 (0.1)	10-30	320	0.67	/	/	DSV	[5]
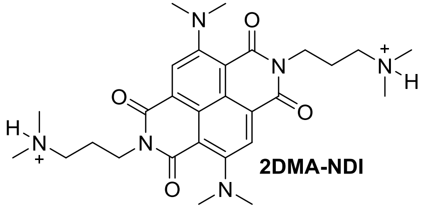 2DMA-NDI		0.05 (0.1)	10-30	320	0.65	/	/	DSV	[5]
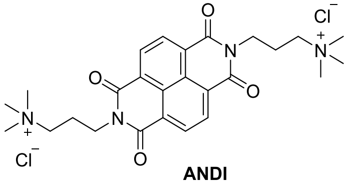 ANDI	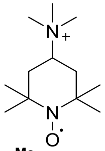 N^{Me}-TEMPO	0.25 (0.5)	40	1002	/	100	0	DSV	[6]
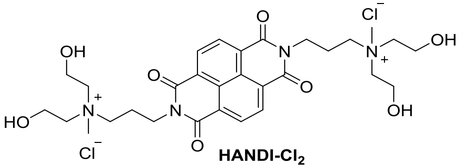 HANDI-Cl_2		0.25 (0.5)	40	3575	/	91.4	0.0024	DSV	[6]

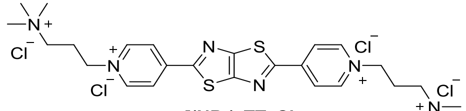 [(NPr)$_2$TTzCl$_4$]	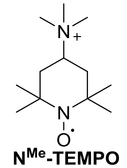 N^{Me}-TEMPO	0.1 (0.2)	40	300	1.44	90	0.03	/	[7]
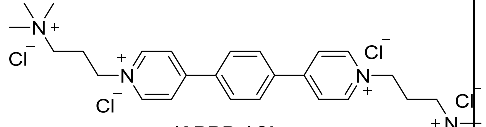 (APBPy)Cl$_4$	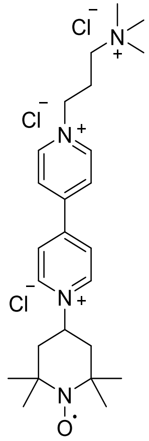 (TBABPy)Cl$_3$	0.5 (1.0)	80	100	1.73	96	0.04	/	[8]
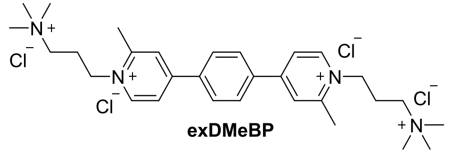 exDMeBP	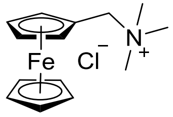 FcNCl	0.5 (1.0)	80	100	/	83	0.17	/	[9]
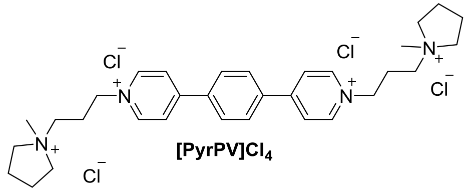 [PyrPV]Cl$_4$	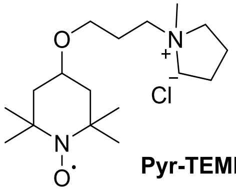 Pyr-TEMPO	0.25 (0.50)	40	1000	1.57	50	0.05	DSV	[10]

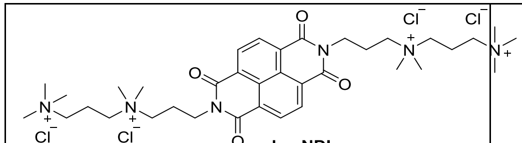 dex-NDI	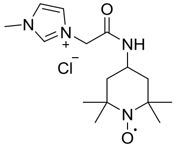 MiAcNH-TEMPO	1.2 (2.4)	60	100	1.27	100	0	DSVN	[11]
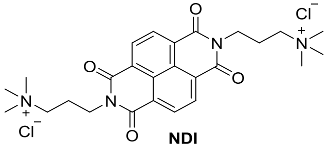 NDI		1.0 (2.0)	40	500	1.31	85.83	0.028	DSVN	[11]
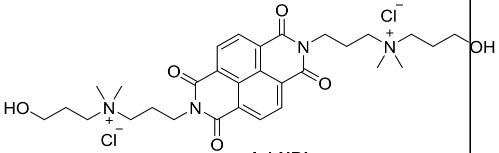 siol-NDI		1.0 (2.0)	40	500	1.29	87.79	0.024	DSVN	[11]
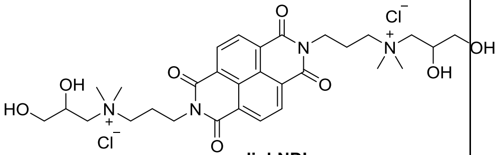 diol-NDI		1.0 (2.0)	40	500	1.30	80.58	0.039	DSVN	[11]
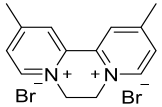 2,2′-bipyridylium	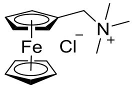 FcNCl	0.5 (0.5)	5	100	1.12	80	0.2	AMV	[12]
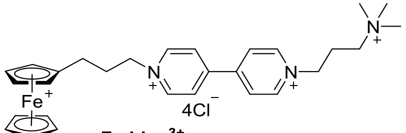 Fc-bipy^{3+}	Fc-bipy^{3+} 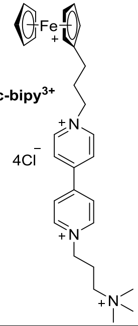	0.5 (0.5)	10	4000	0.7	75	0.0063	anion-exchange membrane	[13]

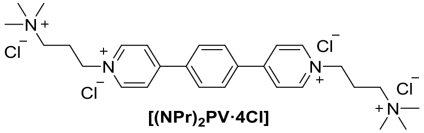 **[(NPr)$_2$PV·4Cl]**	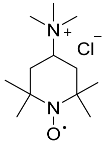 **N^{Me}-TEMPO**	0.1 (0.2)	20	300	1.71	82.8	0.057	AMV	[14]
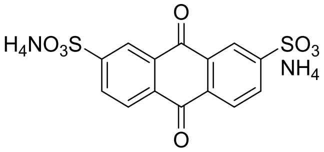 **AQDS(NH$_4$)$_2$**	**NH$_4$I**	0.75 (1.5)	60	300	0.865	100	0	Nafion 115	[15]

Reference

1. Liu X, Zhang H, Liu C *et al.* Commercializable naphthalene diimide anolytes for neutral aqueous organic redox flow batteries. *Angew Chem Int Ed* 2024; **63**: e202405427.
2. Liu X, Zhang X, Bao C *et al.* Arylene diimide derivatives as anolyte materials with two-electron storage for ultrastable neutral aqueous organic redox flow batteries. *CCS Chem* 2023; **5**: 2334-2347.
3. Singh V, Kwon S, Choi Y *et al.* Controlling π-π interactions of highly soluble naphthalene diimide derivatives for neutral pH aqueous redox flow batteries. *Adv Mater* 2023; **35**: e2210859.
4. Medabalmi V, Sundararajan M, Singh V *et al.* Naphthalene diimide as a two-electron anolyte for aqueous and neutral pH redox flow batteries. *J Mater Chem A* 2020; **8**: 11218-11223.
5. Wiberg C, Evenas L, Busch M *et al.* Naphthalene diimides (ndi) in highly stable pH-neutral aqueous organic redox flow batteries. *J Electroanal Chem* 2021; **896**: 115224.
6. Pan MG, Wang W, Wang HZ *et al.* High-voltage and durable pH-neutral aqueous redox flow batteries based on quaternary ammonium cations functionalized naphthalene diimide and nitroxyl radical systems. *J Power Sources* 2023; **580**: 233269.
7. Luo J, Hu B, Debruler C *et al.* A π-conjugation extended viologen as a two-electron storage anolyte for total organic aqueous redox flow batteries. *Angew Chem Int Ed* 2018; **57**: 231-235.
8. Hu SZ, Li TY, Huang MB *et al.* Phenylene-bridged bispyridinium with high capacity and stability for aqueous flow batteries. *Adv Mater* 2021; **33**: e2005839.
9. Tang G, Liu Y, Li Y *et al.* Designing robust two-electron storage extended bipyridinium anolytes for pH-neutral aqueous organic redox flow batteries. *JACS Au* 2022; **2**: 1214-1222.
10. Pan MG, Gao LZ, Liang JC *et al.* Reversible redox chemistry in pyrrolidinium-based TEMPO radical and extended viologen for high-voltage and long-life aqueous redox flow batteries. *Adv Energy Mater* 2022; **12**: 2103478.
11. Liu X, Zhang H, Liu C *et al.* Commercializable naphthalene diimide anolytes for neutral aqueous organic redox flow batteries. *Angew Chem Int Ed* 2024; **63**.
12. Huang J, Yang Z, Murugesan V *et al.* Spatially constrained organic diquat anolyte for stable aqueous flow batteries. *ACS Energy Lett* 2018; **3**: 2533-2538.
13. Zhu Y, Yang F, Niu Z *et al.* Enhanced cyclability of organic redox flow batteries enabled by an artificial bipolar molecule in neutral aqueous electrolyte. *J Power Sources* 2019; **417**: 83-89.
14. Pan M, Lu Y, Lu S *et al.* The dual role of bridging phenylene in an extended bipyridine system for high-voltage and stable two-electron storage in redox flow batteries. *ACS Appl Mater Interfaces* 2021; **13**: 44174-44183.
15. Hu B, Luo J, Hu M *et al.* A pH-neutral, metal-free aqueous organic redox flow battery employing an ammonium anthraquinone anolyte. *Angew Chem Int Ed* 2019; **131**: 16782-16789.
